# Need Support and Need Thwarting: A Meta-Analysis of Autonomy, Competence, and Relatedness Supportive and Thwarting Behaviors in Student Populations

**DOI:** 10.1177/01461672231225364

**Published:** 2024-01-30

**Authors:** Joshua L. Howard, Gavin R. Slemp, Xiao Wang

**Affiliations:** 1 Monash University, Victoria, Australia; 2 The University of Melbourne, Victoria, Australia; 3 Renmin University of China, Beijing, China

**Keywords:** autonomy support, thwarting behaviors, students, education, self-determination

## Abstract

In this meta-analysis, we review the nomological networks of six need-supportive and need-thwarting categories, as defined by self-determination theory (SDT), and as they apply to students in educational contexts. We conducted a synthesis of 8693 correlations from 637 samples (*N* = 388,912). A total of 72 covariates were examined, resulting in 183 meta-analytic effects reported. Results indicate that teachers and parents who experience psychological need satisfaction and well-being are seen as more supportive. Supportive teacher behaviors correlated positively with a range of desired student outcomes, including performance, engagement, and well-being. Thwarting behaviors tended to display the opposite pattern. Our results are consistent with the theoretical expectations of SDT, yet questions remain concerning the incremental validity of these constructs. We highlight the need for further research on (a) factors that cause teachers to provide support and (b) the specific behaviors within each category to distinguish these categories and increase practical utility.

Student experiences in educational institutions can have a substantial impact on how children develop and whether they persist with education (Grolnick & Lerner, 2023; Joussemet et al., 2008), making this a central topic of interest to many researchers and teachers. However, controlling forms of teaching—whereby teachers impose restraints on students’ thinking, feeling, or behaviors—are relatively common (Reeve, 2009), and educational institutions continue to maintain controlling structures and practices (Ryan & Weinstein, 2009) despite growing evidence highlighting the importance of more supportive environments (Guay, 2022). For these reasons, understanding how different teaching practices influence students across their schooling years is relevant for educational and developmental researchers, as well as school leaders, teachers, and policymakers. Self-determination theory (SDT) is one widely applied theory of motivation that informs much of this research (Ryan & Deci, 2017). From the SDT point of view, the objective of educational institutions is to enable students to flourish with respect to classroom functioning, engagement, social development, and well-being (Ryan et al., 2023). Key to achieving this is the provision of conditions that allow students to satisfy their needs for autonomy, competence, and relatedness, which has downstream benefits for learning and well-being.

The aim of this study is to synthesize the literature relating to need-supportive and need-thwarting educational environments. In particular, we examine six forms of supportive and thwarting behaviors across a broad array of covariates: autonomy support, competence support, relatedness support, as well as autonomy thwarting, competence thwarting, and relatedness thwarting. With the rapid growth of this literature and the specification of several new constructs and scales (Aelterman et al., 2019; Bartholomew, Ntoumanis, Ryan, Bosch, & Thøgersen-Ntoumani, 2011; Gilbert et al., 2021), questions may be asked concerning the relative contribution and functioning of these six variables. Therefore, we seek to answer five research questions: What types of supportive and thwarting behaviors are studied and how do they associate with one another? What theoretical antecedent factors, for teachers and students, are associated with supportive and thwarting contexts? What supporter-related outcomes are associated with supportive and thwarting behaviors? What student-focused outcomes are observed when teachers use different supportive or thwarting behaviors? Finally, to what degree does each form of need support and need thwarting contribute uniquely? We hope to identify the relative usefulness of these constructs, offer recommendations regarding their practical use and unique contribution, and highlight areas that require further research.

## Self-Determination Theory

SDT is now widely applied across domains of research. Central to the theory are the basic psychological needs for autonomy, competence, and relatedness, which, when satisfied, lead to optimal human functioning (Deci & Ryan, 2000; Vansteenkiste et al., 2020). More specifically, the satisfaction of these basic needs leads to more autonomous forms of motivation by prompting people to more deeply internalize the value of their behavior—a process known as internalization (Deci et al., 1994). This subsequently leads to positive student outcomes, including both performance and well-being. In support of this, a recent meta-analysis reviewed the student outcomes associated with each type of motivation specified within SDT and demonstrated that more autonomous forms of motivation consistently related positively to desirable outcomes (Howard et al., 2021). Specifically, autonomous forms of motivation were positively associated with positive affect, vitality, and enjoyment (most strongly related to intrinsic motivation), engagement and persistence (associated primarily with identified regulation), and both objective and student-reported grade point average (GPA). In a related meta-analysis of studies from physical education contexts, Vasconcellos and colleagues (2020) found that a range of affective, behavioral, and cognitive outcomes were positively associated with autonomous forms of motivation and basic psychological need satisfaction. Recent meta-analytic structural equation modeling provides further support for the theoretical connection of basic psychological need satisfaction to desirable forms of motivation and subsequent outcomes in educational settings (Bureau et al., 2022).

The satisfaction of basic psychological needs can arise from two main sources. First are internal characteristics, including causality orientations and life aspirations, which refer to relatively enduring motivational tendencies that predispose people toward feeling autonomous or controlled (Bradshaw et al., 2023; Hagger & Hamilton, 2021; Kasser & Ryan, 1996). The second main source comprises the immediate context surrounding the student. This study focuses on the latter of these and thereby examines the role that teachers and parents play in creating environments that nurture or obstruct students’ basic needs.

## Need-Supportive Behaviors

Within SDT, a range of behaviors have been studied that are thought to be particularly influential environmental precursors to basic need satisfaction. These generally fall into two opposing subclassifications: supportive behaviors and thwarting behaviors. Supportive behaviors encompass those that are thought to nurture students’ basic needs, whereas thwarting behaviors encompass those that are thought to obstruct students’ basic needs (detailed in more depth shortly). The most studied type of support is *autonomy support*, which refers to a cluster of behaviors that are intended to nurture experiences of autonomy in students, including, for example, providing meaningful choices to students, seeking out and acknowledging student perspectives, avoiding the use of external rewards or sanctions to motivate behavior, and offering meaningful rationales for behavior (Ahmadi et al., 2023; Gilbert et al., 2021). Whereas an autonomy-supportive teacher will not necessarily acquiesce to every demand of a child, they will make efforts to empower students to take ownership of their learning while also providing basic guidance to direct them along the way (Reeve & Cheon, 2021).

More recently, *competence support* and *relatedness support* have begun to be measured separately. Competence-supportive behavioral strategies include the provision of task-specific guidance and feedback, encouraging student goal setting, and ensuring optimal challenge (Ahmadi et al., 2023; Gilbert et al., 2021). Such behaviors are theoretically more likely to enhance student perceptions of competence, while perhaps being less central for the autonomy or relatedness needs. The term “structure” is often used synonymously with competence support (Aelterman et al., 2019). Similarly, relatedness-supportive strategies, such as demonstrating warmth and affection toward students, conveying unconditional positive regard, and taking steps to demonstrate empathy and understanding (Ahmadi et al., 2023; Gilbert et al., 2021), are behaviors likely to primarily satisfy students’ need for relatedness.

There is growing evidence for the positive effects of the three supportive behavior categories, with autonomy support being the most established. For example, evidence for autonomy support is robust with a meta-analysis by Okada (2023) demonstrating that it is positively correlated with academic achievement in higher education, whereas Vasquez and colleagues (2016) concluded that autonomy support is likely a minor but important contributor to student success at school more broadly. Bureau and colleagues (2022) concluded that autonomy support from teachers and parents uniquely predicts need satisfaction and subsequently motivation types, providing clear support for this central premise of SDT. Indeed, intervention studies have also supported the positive impact of autonomy-supportive teaching on student outcomes (Reeve & Cheon, 2021; Su & Reeve, 2011). Existing research therefore supports the importance of autonomy support in education. While research points toward the competence- and relatedness-supportive behavioral strategies being comparably effective (e.g., Vasconcellos et al., 2020), there is less available research on these behaviors.

## Need-Thwarting Behaviors

Whereas need-supportive behaviors cultivate students’ basic needs, need-thwarting behaviors actively frustrate basic needs (Ryan & Deci, 2017). *Autonomy-thwarting* behaviors, also known as controlling behaviors, have been studied for over a decade and represent the negatively valenced opposite of autonomy support (Bartholomew, Ntoumanis, Ryan, Bosch, & Thøgersen-Ntoumani, 2011; also see Skinner et al., 2005). For instance, autonomy-thwarting teaching entails tactics such as prescribing goals and applying pressure until students meet these goals, the use of commands, intimidation, and scolding (Reeve & Cheon, 2021), or using rewards and punishments to obtain compliance. These can be tangible rewards such as performance contingent prizes or punishments such as “writing lines” in which a statement is written repeatedly a specified number of times. Psychologically focused rewards and punishments can also be used including, for example, the use of conditional regard in which approval is only shown when students achieve performance or behavior goals (Reeve & Cheon, 2021).

Within SDT, autonomy-thwarting behaviors are categorized as distinct from autonomy-supportive behaviors, creating what is often referred to as a dual-process model including “bright” and “dark” pathways to motivation and associated outcomes (Vansteenkiste et al., 2020). This distinction is based on the observation that these two categories are consistently distinguished in measurement models, are generally correlated moderately negatively, and associate with outcomes differently. For instance, supportive behaviors generally associate more strongly with positive outcomes, while thwarting behaviors generally associate more strongly with undesirable outcomes (Bartholomew, Ntoumanis, Ryan, Bosch, & Thøgersen-Ntoumani, 2011; Reeve & Cheon, 2021). This model also implies that students can simultaneously experience support and thwarting from teachers, and therefore report high levels of both concurrently (Haerens et al., 2018). Given the increasing attention being paid to autonomy-thwarting contexts, as well as their relevance in practice, we include these behaviors in our meta-analysis.

Recent research has also specified competence thwarting and relatedness thwarting, which have been proposed as negatively valenced versions of competence support and relatedness support, respectively (Ahmadi et al., 2023). *Competence-thwarting* behaviors occur when socializing agents, such as teachers or parents, use behaviors that generally make people feel ineffective or lacking in capability, or serve as obstacles to growth (e.g., Gunnell et al., 2013). It encompasses behaviors such as criticizing characteristics of a person that they cannot change, providing vague or non-instructive feedback or criticism, or focusing heavily on competition and winning (Ahmadi et al., 2023; Gilbert et al., 2021). Chaos is a concept closely linked with competence thwarting (Aelterman et al., 2019), which generally involves the lack of structure, support, or feedback that ultimately leaves students confused and impedes their growth. *Relatedness-thwarting* behaviors include when teachers or parents do not convey interest and care for the student. It generally involves behaviors such as remaining cold and unavailable to students, using harsh and intimidating language or tactics, and applying conditional positive regard (Ahmadi et al., 2023). Scales for these constructs are recent additions and, as such, have been applied more sparingly, although interest in them is growing.

## Overview and Research Questions

Together, these different strategies yield six categories of behaviors that influence students’ basic needs: autonomy support, competence support, relatedness support, autonomy thwarting, competence thwarting, and relatedness thwarting (Skinner et al., 2005). According to SDT, each of these categories is composed of specific and unique behaviors, and each should be associated with covariates differently. In this study, we examine in detail not only the student-focused correlates of autonomy support but also other need-supportive and need-thwarting behaviors. In addition, rather than focusing solely on the theoretically positioned outcomes of supportive behaviors, we also seek to examine factors that may predispose teachers or parents to create or foster need-supportive (or thwarting) educational environments.

We used the SDT causal sequence to categorize covariates as antecedents or outcomes (see Deci et al., 2017; Ng et al., 2012; Ryan & Deci, 2017 for overviews). This framework suggests that supportive or thwarting behaviors and contexts influence student need satisfaction or frustration as the most proximal outcomes, which, in turn, influences student motivation, which subsequently influences more distal outcomes (e.g., well-being, performance, or behavior). Factors that precede this process, including more enduring student-related characteristics that do not tend to change much (e.g., demographic factors, personality traits), were considered antecedents to the provision/perception of supportive or thwarting behaviors. Similarly, we also include as antecedents the internal teacher or parent characteristics that we expect are likely to precede instances of need support or thwarting, including their own need satisfaction, well-being, personality, or demographic characteristics. As noted in the discussion, the direction of causality implied here is not always guaranteed, and indeed due to the correlational nature of this analysis, cannot be inferred from the results.

In conducting our meta-analysis, we make several contributions. First, we examine the literature beyond autonomy support and also include competence- and relatedness-supportive behaviors, which have been receiving increasing empirical attention in recent years (e.g., Gilbert et al., 2021). Second, given the growing interest in thwarting behaviors (Bartholomew, Ntoumanis, Ryan, Bosch, & Thøgersen-Ntoumani, 2011), we also seek to capture autonomy-, competence-, and relatedness-thwarting behaviors to examine the relative impact these have on the educational context. As such, we provide a novel comparison of supportive and thwarting approaches in educational contexts and examine the degree to which their distinctions hold up across a range of covariates. To better understand these central variables, we aim to answer five research questions.

**Research Question 1:** How are need-supportive and need-thwarting behaviors correlated?

Our first question concerns the relationships between the categories of supportive and thwarting behavior categories and the degree to which they covary. To do this, we first examine the correlations between these variables. We then also examine the constituent behaviors unique to each category of support/thwarting and examine how these individual behaviors correlate.

**Research Question 2:** What theoretical antecedent factors from students and teachers/parents correlate with need-supportive and need-thwarting behaviors?

We examine factors that are associated with teachers and parents providing supportive educational contexts to students, and what factors may predispose students to viewing a context as supportive. Based on existing work (Gillison et al., 2019; Grolnick et al., 2002; Ntoumanis et al., 2021; Reeve, 2012), we position student individual differences such as causality orientations, personality, as well as demographics (age and socio-economic status) as antecedents of support/thwarting perceptions. From teachers and parents, we examine the degree to which their well-being, need satisfaction, and level of education correlate with their provision of need-supportive environments.

**Research Question 3:** How are need-supportive and need-thwarting behaviors correlated with teacher and parent outcomes?

We also examine what outcomes for the teachers and parents are correlated with the provision of supportive behaviors. Specifically, we examine how supportive behaviors associate with the perceived relationship quality with their student and their perceived teaching quality.

**Research Question 3:** How are need-supportive and need-thwarting behaviors associated with student outcomes?

We examine the student-focused outcomes that have been associated with various types of support. We break this down into four main categories representing learning outcomes (GPA, educational performance, and learning strategy use), motivational outcomes (motivational beliefs and self-efficacy), engagement outcomes (persistence, prosocial, and proactive behaviors, misconduct, and intentions), and well-being outcomes (emotional well-being, eudemonic well-being, depression, anxiety, and physical health).

**Research Question 5:** What is the unique contribution of each dimension of need-supportive and need-thwarting behaviors?

The final research question is informed by all previous results, as well as relative weights analysis (RWA), and seeks to assess the extent to which each of the six central categories is empirically distinct. To be distinct, behavior categories should not be correlated excessively highly and should have unique associations across a range of different covariates. Failing to meet these empirical requirements may indicate either the constructs are redundant, or that they need to be conceptualized and measured more precisely. After presenting a range of meta-analytic findings to inform each of these research questions, we discuss potential directions for future research.

## Method

### Literature Search

Due to the sheer size of the literature and the number of studies involved, we conducted our search in two stages. The initial search, executed by the second author, was part of a larger project examining supportive behaviors across all domains of research (including sport, workplaces, parenting, health, physical education, as well as education; Slemp et al., in press). This search took place on November 27, 2021, with subsequent coding taking approximately 8 months to complete. As this search strategy was specifically designed to capture records on the supportive behaviors, it was likely we were missing studies on need-thwarting behaviors. Thus, to capture this research, the first and third authors ran an additional search that took place on December 10, 2022. This study was not pre-registered. All data, analysis code, and supplementary results are available in the online supplementary files (https://osf.io/6aqfw/?view_only=dd2d64fcb3c2490e9770413c92515a2f).

**Figure 1.**
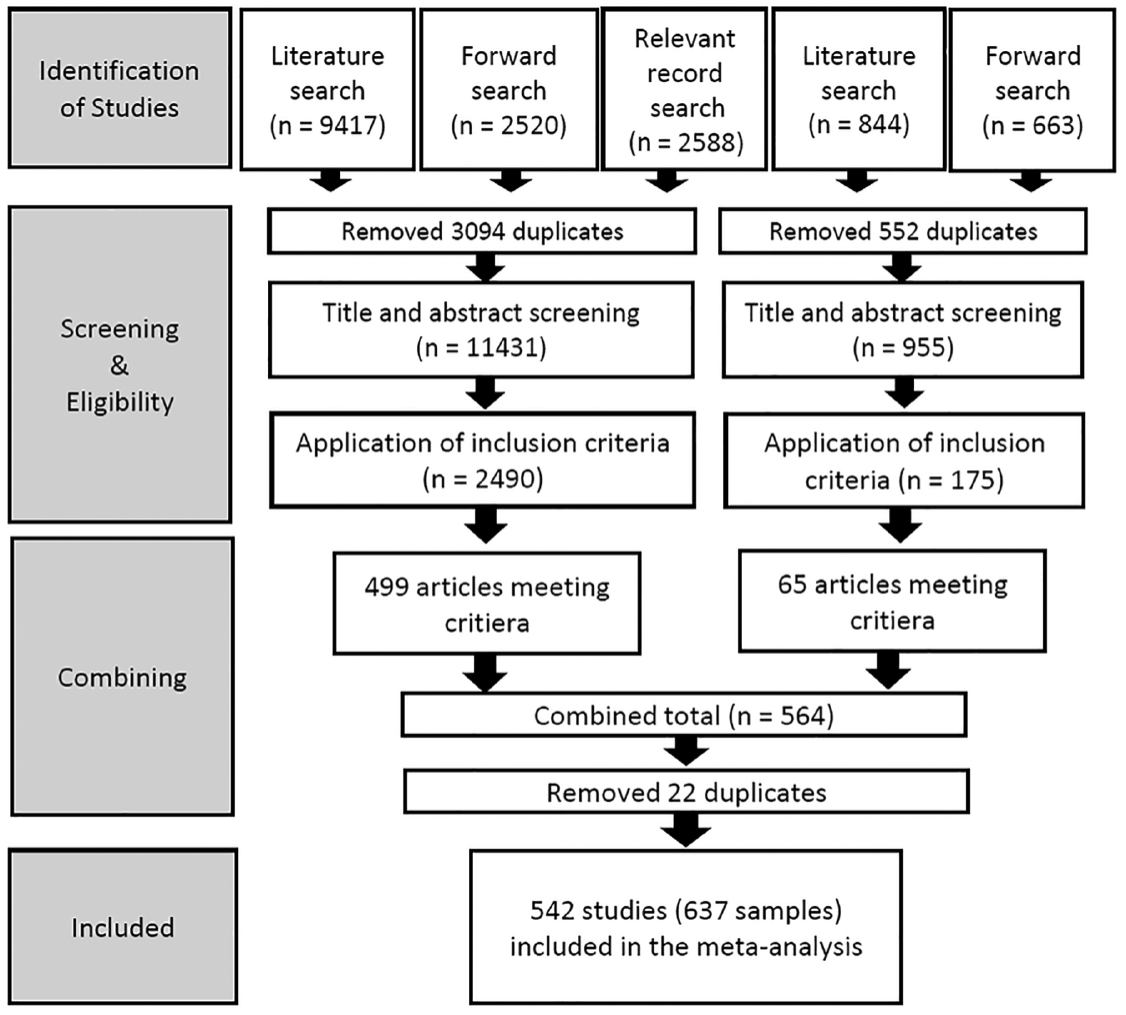
Depiction of the search procedure.

Our search strategy for both supportive and thwarting behaviors was coordinated and involved three near-identical approaches (see Figure 1). First, we conducted searches of seven electronic databases for relevant records: PsycINFO; MEDLINE; SPORTDiscus; CINAHL; Web of Science; Educational Resources Information Center (ERIC); and Scopus. These databases were selected on the basis that they provided broad coverage of social science research. Our search terms were selected to capture a variety of behaviors to support or thwart basic psychological needs. For supportive behaviors, we used the following terms: support* for autonom* OR needs support* OR autonom*-support* OR *competence-support”* OR support* for competence OR relatedness-support* OR support* for relatedness OR self determin*. For the thwarting behaviors, we used the following equivalent string: autonom* thwart* OR autonom* control* OR competence* thwart* OR relatedness* thwart*. While alternative language is sometimes used in the SDT literature to describe supportive or thwarting behaviors (e.g., structure, involvement, and chaos), because these terms are also commonly used to describe very general phenomena across a wide variety of literatures and different subfields, they created unnecessary noise in our search and were thus omitted. Instead, we argue that these papers were captured by using SDT-specific terms with which they tend to co-occur when used in an SDT context. Similarly, they were also generally captured by searching for autonomy support which does not have a comparable synonym, given the different supportive or thwarting behaviors are very typically studied *in addition to* autonomy support. Using this procedure, these searches yielded 9,417 records for support and 844 records for thwarting (total *n* = 10,261 records).

Our next strategy was to use Web of Science to prospectively search for papers citing validated key measures of support/thwarting of basic psychological needs across different contexts (e.g., Aelterman et al., 2019). This process identified a further 2,520 records relating to supportive behaviors, and another 663 relating to thwarting behaviors (total *n* = 3,183 records).

Our final approach was to examine other related sources for relevant records we may have missed, including reference lists from Cochrane library sources, and reference lists of key SDT-related books, literature reviews, empirical papers, and book chapters (e.g., Howard et al., 2017; Mossman et al., 2022; Slemp et al., 2018). The process led to the identification of a further 2,588 relevant records for screening.

### Inclusion Criteria and Coding

Three inclusion criteria were applied when assessing studies: (a) samples must be focused upon student populations in educational contexts, (b) students must have ranged from primary school to university, and (c) at least one zero-order correlation between two types of need support/thwarting, or between a need-supporting/thwarting behavior and a covariate needed to be reported. All authors participated in the coding of studies. A coding sheet was designed to capture all relevant study information (study metadata including full reference and publication status), effect size information (*r*, *n*, reliability scores for each variable, information regarding who was rated, who did the rating for each variable, and time lag between measures), and demographic information (age, gender of participants, and nationality in which the sample was recruited). Each variable was coded precisely as recorded in the primary article before being grouped into more general categories for meta-analysis by the third and first authors (see Table S1 in the supplementary materials). The process of categorizing variables was closely guided by the methods of previous meta-analyses (i.e., Howard et al., 2021; Van den Broeck et al., 2021; Vasconcellos et al., 2020). To further improve precision and reflect the nature of the literature accurately, more specific categories were specified as distinct whenever a suitable number of studies (>3 across at least two types of need support or thwarting) were available to inform them. To establish interrater agreement in coding, a subset of 35% of the initial studies were recoded. An accuracy check revealed 96.39% agreement across all coding decisions. Disagreements were resolved by discussion. Due to this relatively high rate of agreement, the remainder of the included studies were coded by a single coder.

Covariates coded fewer than five times and could not reasonably be combined into a larger group were deemed unsuitable and thus excluded. Any uncertainties were resolved via discussion between the third and first authors, which resulted in the nomological network of covariates examined below. Basic psychological need satisfaction and frustration, as well as motivation types defined by SDT, were coded and analyzed but are not presented in the main manuscript as they have been reviewed more comprehensively elsewhere (Slemp et al., in press), but are presented in the supplementary materials (see Supplemental Tables S3 and S4).

The final database contained 8693 effect sizes from 637 samples (542 studies) and 388,912 participants. A total of 72 covariates were examined, with 183 main meta-analytic effects being estimated and reported. The mean sample size of the included studies was 610 participants. The mean age of the samples was 16.3 and on average 53.26% of the participants were female. Regarding contexts, 42.5% of samples were from classrooms, 33.5% were from educational experiences outside of classrooms (i.e., with parents), and 24% were from physical education contexts. Of the studies, 463 (73%) were cross-sectional in design, 129 (20%) were longitudinal, 31 (5%) were experimental in design, while the remaining 15 (2%) were dairy studies. Data were collected from 56 different national contexts (see Supplemental Table S2), with 118 samples (19.3%) from the United States alone. China (*n* = 60, 9.8%), Spain (*n* = 53, 8.6%), Belgium (*n* = 51, 8.3%), and Canada (*n* = 46, 7.5%) were the next most often sampled countries.

### Data Analysis

All meta-analytic calculations were carried out in the Psychmeta package (Dahlke & Wiernik, 2019) for R software version 4.3.0 (R Core Team, 2023). A random effects model was used throughout. In many instances, studies reported multiple effects that needed to be analyzed together, breaching the assumption of independence inherent in meta-analysis. In such cases, the “composite variable” function was used in the psychmeta package to combine these non-independent estimates into a single composite variable, thus ensuring the independent sample assumption and therefore preventing inflation of sample sizes and estimate precision. When scale reliability data were not coded, an average alpha coefficient was calculated for that variable, and this average score was imputed into missing cells (see Supplemental Table S5 for all calculated alpha coefficients). Outliers and influential studies were examined via the “sensitivity” command in psychmeta. One highly influential study was identified (i.e., Wang et al., 2021). Due to the extremely large sample size (*n* = 513,295), correlations from this study are weighted much more highly in the meta-analyses. Based on the sensitivity analyses and the substantial impact this study had on results, it was decided to remove this study from the meta-analysis.

We report sample-size weighted correlations (*r*), 95% confidence intervals associated with the estimated effect, and the sample size on which the estimate is based (where *k* is the number of independent samples analyzed and *n* is the total number of participants from those studies). Effect sizes are interpreted in line with the benchmarks proposed by Gignac and Szodorai (2016) in which a correlation of .10 is considered small, .20 is typical, and .30 is relatively large.

Homogeneity of estimated effects was examined via Tau (t), Tau-squared (t^2^), the 80% credibility intervals, as well as the I^2^ statistic. Tau and Tau^2^ are descriptive statistics indicating the amount of variance present in estimates. The 80% credibility interval depicts the spread of underlying effects, indicating that 80% of true effects will fall within this range (Schmidt & Hunter, 2014). The I^2^ statistic indicates what proportion of the observed variance is likely to be explained by moderator variables, as opposed to study-level artifacts such as sampling error and chance (Higgins et al., 2003; Higgins & Thompson, 2002). An I^2^ score above 75% indicates substantial heterogeneity and the presence of moderator variables, while 50% is considered moderate, and 25% indicates low heterogeneity within the estimated effect. Together these statistics indicate the heterogeneity of the underlying effects (i.e., between-studies variance).

Publication bias was investigated via the Eggers regression test and examination of funnel plots. Eggers regression test results can be found in Supplemental Tables S6 to S9. Funnel plots for each estimated effect are presented in the online OSF repository. Publication bias was relatively infrequent with Eggers regression test indicating 32 out of 175 estimated effects returning significant results.

RWA was conducted to test the differential unique effects associated with each type of need-supportive and need-thwarting behavior (Tonidandel & LeBreton, 2015). This analysis identifies the amount of variance in a predicted outcome that can be attributed uniquely to each predictor variable while accounting for multicollinearity. Multicollinearity occurs in regression-based analyses when the variance explained in an outcome is misattributed between predictors due to high correlations. As such, this type of analysis is increasingly necessary when predictor variables (i.e., need-supportive and need-thwarting variables) are highly correlated. This analysis was conducted from correlation tables of meta-analytically derived results between support and thwarting variables as well as various covariates. As our goal was to compare the incremental contribution of each type of supporting and thwarting behavior, we did this for each covariate that had more than three types of support/thwarting behaviors associated with it in our meta-analyses. Output from this analysis provides an estimate of the variance associated with each predictor after accounting for multicollinearity, as well as the proportion (depicted as a percentage) of the total explained variance. These analyses were conducted in the R software program.

## Results

### Correlations Between Support and Thwarting Behaviors

The first set of analyses examined the associations between each category of need-support and need-thwarting behaviors. Results of these analyses are summarized in Table 1. The correlations between autonomy, competence, and relatedness support were positive and very strong in magnitude (*r* = .64-.68). Likewise, correlations between three categories of need thwarting were also positive and strong (*r* = .56-.66). On average, need-supportive variables correlated –.32 with need-thwarting variables (range –.21 to –.47).

**Table 1.** Correlation Table for Supportive and Thwarting Categories.

Types of support/thwarting	Autonomy support	Competence support	Relatedness support	Autonomy thwarting	Competence thwarting	Relatedness thwarting	Need support	Need thwarting
Autonomy support	—	124	72	197	26	26	16	13
Competence support	.64	—	52	29	22	19	11	8
Relatedness support	.68	.66	—	15	11	13	9	5
Autonomy thwarting	–.27	–.21	–.27	—	9	16	4	3
Competence thwarting	–.29	–.31	–.37	.56	—	7	2	2
Relatedness thwarting	–.33	–.38	–.47	.56	.66	—	2	2
Need support	.53	.53	.53	–.36	–.61	–.63	—	7
Need thwarting	–.35	–.51	–.55	.73	.86	.83	–.61	—

*Note.* Meta-analytic correlation estimates below the diagonal, number of studies (*k*) above the diagonal.

We were also able to estimate correlations between perceptions of autonomy support and a range of specific behaviors falling under the supporting and thwarting behavior categories. Results are presented in Table 2. The strongest individual correlation with general autonomy support was warmth (a specific behavior that falls under relatedness support) at .57 (*k* = 14), followed by participatory behaviors (a key facet of autonomy support) at .53. The range of competence-supporting behaviors (i.e., structure, monitoring, clarifying, directive support, and expectations) were positively and moderately strongly correlated with autonomy support with a mean correlation of .33 (range .24-.39). Autonomy-, competence-, and relatedness-thwarting behaviors (control, coercion, helicopter parenting, chaos, conditional regard, intimidation, and cold) were typically negatively related to autonomy support (*r_M_* = –.22). It is interesting to note that chaos (i.e., competence thwarting) and helicopter parenting (autonomy thwarting) were not significantly related to autonomy support.

**Table 2.** Correlations Between Specific Behaviors and Support Composites.

Support behaviors	*k*	*n*	*r*	95% Confidence interval		80% Credibility interval		*t* ^2^	*t*	I^2^
				Lower	Higher	Lower	Higher			
Autonomy support										
Participative/rationales	4	2,033	.53	0.33	0.74	0.33	0.74	0.02	0.12	93.72
Structure	67	29,187	.36	0.29	0.42	0.00	0.71	0.08	0.28	97.72
Monitoring	9	15,190	.32	0.27	0.36	0.23	0.40	<0.01	0.06	87.94
Clarifying / feedback	5	8,588	.24	–0.10	0.58	–0.17	0.66	0.07	0.27	99.32
Directive support / guiding	7	15,500	.36	0.22	0.50	0.14	0.57	0.02	0.15	98.51
Expectations	6	13,338	.39	0.31	0.47	0.28	0.50	0.01	0.07	94.42
Involvement	42	24,423	.44	0.38	0.49	0.22	0.66	0.03	0.17	96.18
Warmth	14	7,274	.57	0.49	0.65	0.39	0.75	0.02	0.13	95.54
Control	162	81,111	–.27	–0.30	–0.23	–0.56	0.02	0.05	0.22	96.68
Coercion	4	916	–.25	–0.47	–0.04	–0.45	–0.06	0.01	0.12	78.72
Helicopter parenting	8	1,936	–.05	–0.29	0.20	–0.45	0.36	0.08	0.29	95.21
Chaos	15	5,806	–.07	–0.19	0.04	–0.34	0.19	0.04	0.20	93.96
Conditional regard	14	7,965	–.29	–0.39	–0.18	–0.52	–0.05	0.03	0.17	95.24
Intimidation	5	4,451	–.36	–0.42	–0.30	–0.42	–0.30	<0.01	0.04	63.62
Cold/abandoning	9	3,509	–.22	–0.33	–0.10	–0.41	–0.02	0.02	0.14	88.92
Competence support										
Participative/rationales	5	2,697	.45	0.32	0.57	0.30	0.59	0.01	0.10	88.66
Structure	30	17,844	.36	0.25	0.47	–0.02	0.75	0.09	0.29	98.18
Involvement	26	15,655	.38	0.29	0.47	0.10	0.67	0.05	0.22	97.55
Control	16	6,457	–.12	–0.21	–0.02	–0.35	0.11	0.03	0.17	92.45
Chaos	14	47,63	–.20	–0.30	–0.10	–0.43	0.03	0.03	0.17	91.27
Warmth	7	3,045	.61	0.45	0.77	0.37	0.85	0.03	0.17	96.80
Cold/abandoning	9	5,217	–.29	–0.37	–0.20	–0.43	–0.15	0.01	0.10	87.65

Similar analyses were conducted with the competence support composite factor and all possible individual behaviors. The number of samples available for these analyses was significantly fewer, given competence support is a more recent addition to the literature and has received less research attention. Warmth was again the strongest correlate (*r* = .61, *k* = 7), followed by participatory behaviors (*r* = .45, *k* = 5). Cold/abandoning and controlling behaviors were negatively related. In line with theoretical expectations (Aelterman et al., 2019), chaos was significantly negatively associated with competence support (*r* = –.20, *k* = 14).

### Antecedents of Support

#### Individual Differences

Our second research question examined antecedent factors to students perceiving support, as well as antecedent factors for teachers and parents providing support (Table 3). For students, we were able to examine student causality orientations, personality, age, and socio-economic status for their correlations with perceived need-supportive behaviors. Data were not available for some need-supportive behaviors and were universally absent for need-thwarting behaviors. Student autonomous causality orientation correlated positively with both perceptions of autonomy support (*r* = .28, *k* = 12) and competence support (*r* = .36, *k* = 6), whereas controlled causality orientation was not significantly related to perceptions of autonomy support. All personality traits were significantly correlated with autonomy support, with agreeableness (*r* = .23), conscientiousness (*r* = .27), extraversion (*r* = .17), and openness to experience (*r* = .15) all positively correlated with autonomy support and neuroticism significantly negatively correlated (*r* = –.17). These findings are in line with research from SDT in other contexts (La Guardia & Ryan, 2007; Sheldon et al., 1997).

**Table 3.** Associations With Student Demographic and Individual Difference Variables.

Covariates	*k*	*n*	*r*	95% confidence interval		80% credibility interval		*t* ^2^	*t*	I^2^
				Lower	Higher	Lower	Higher			
Autonomous causality orientation										
Autonomy support	12	1,772	.28	0.17	0.39	0.07	0.49	0.02	0.15	79.62
Competence support	6	1,047	.36	0.19	0.53	0.15	0.58	0.02	0.14	82.66
Controlled causality orientation										
Autonomy support	11	1,477	–.09	–0.25	0.07	–0.39	0.21	0.05	0.22	86.68
Competence support	5	707	–.09	–0.29	0.11	–0.30	0.12	0.02	0.14	72.28
Agreeableness										
Autonomy support	7	6,396	.23	0.14	0.31	0.10	0.35	0.01	0.08	87.91
Conscientiousness										
Autonomy support	6	5,826	.27	0.20	0.33	0.18	0.35	<0.01	0.06	78.47
Extraversion										
Autonomy support	7	5,928	.17	0.12	0.22	0.10	0.24	<0.01	0.05	64.83
Neuroticism										
Autonomy support	7	6,183	–.17	–0.26	–0.08	–0.30	–0.04	0.01	0.09	88.11
Openness										
Autonomy support	6	5,826	.15	0.04	0.25	0.00	0.29	0.01	0.10	90.79
Age										
Autonomy support	65	33,544	–.04	–0.08	–0.01	–0.22	0.13	0.02	0.13	90.35
Competence support	6	4,197	–.05	–0.12	0.01	–0.12	0.02	<0.01	0.05	61.09
Relatedness support	8	3,556	.00	–0.09	0.10	–0.14	0.15	0.01	0.10	82.91
Socio-economic status										
Autonomy support	30	18,084	.04	0.01	0.07	–0.09	0.16	0.01	0.09	88.87
Competence support	4	2,545	.08	–0.05	0.20	–0.03	0.19	<0.01	0.07	90.87
Relatedness support	6	3,720	.12	–0.01	0.25	–0.05	0.29	0.01	0.11	94.47

#### Age and SES

Student age was found to correlate negatively with autonomy support, which was significant but very small in magnitude (*r* = –.04) indicating that as students grow older, they tend to perceive or receive less autonomy support (Gillet et al., 2012; Martinek et al., 2016; Matte-Gagné et al., 2013). Competence and relatedness support were not significantly correlated with age, potentially due to more limited power to detect the small effect (*k* = 6 and 8, respectively). Socio-economic status observed a very weak yet significant positive association with autonomy support (*r* = .04), suggesting that autonomy support is more likely to occur for students higher in SES. Competence support and relatedness support had similar effect sizes (*r* = .08 and .12, respectively) though were not significant.

#### Supporter Characteristics

We next examined factors considered predictors of whether teachers and parents provide autonomy support to students. As displayed in Table 4, these results were relatively sparse. Parents who themselves reported greater levels of psychological need satisfaction were also perceived to provide greater autonomy support (*r* = .37, *k* = 14). Likewise, parental need frustration and ill-being were negatively correlated with the perceived provision of autonomy support (*r* = –.19 and –.14, respectively). The correlation between teacher need satisfaction and autonomy support was also positive though not significant (*r* = .15, *k* = 4). These results indicate that the well-being and psychological flourishing of people providing the support is an important covariate of autonomy support provision. The education level attained by parents was also positively associated with autonomy support provision (*r* = .12, *k* = 25), indicating that more highly educated parents may be more likely to provide an autonomy-supportive educational environment.

**Table 4.** Associations With Predictors of Support Provision.

Covariates	*k*	*n*	*r*	95% Confidence interval		80% Credibility interval		*t* ^2^	*t*	I^2^
				Lower	Higher	Lower	Higher			
Teacher need satisfaction										
Autonomy support	4	2,400	.15	–0.03	0.34	–0.02	0.33	0.01	0.11	88.01
Parent need satisfaction										
Autonomy support	14	2,513	.37	0.24	0.50	0.08	0.65	0.04	0.21	91.43
Parent need frustration										
Autonomy support	8	1,409	–.19	–0.29	–0.09	–0.32	–0.06	0.01	0.09	61.65
Parent ill-being										
Autonomy support	8	3,895	–.14	–0.22	–0.05	–0.26	–0.01	0.01	0.09	78.93
Parent education level										
Autonomy support	25	10,869	.12	0.06	0.18	–0.06	0.30	0.02	0.14	89.14

### Outcomes for Teachers and Parents

We then investigated the correlation between autonomy support and perceptions of relationship quality among teacher–student, peer–student, and parent–student dyads, as well as the correlation between teacher autonomy support and student ratings of teaching quality (Table 5). Autonomy support was strongly and positively associated with teacher–student relationship quality (*r* = .52, *k* = 6), and to a lesser extent with parent–student (*r* = .36) and peer–student (*r* = .36) relationship quality, suggesting that autonomy support goes hand in hand with better perceived quality of relationships in education contexts. Somewhat suspiciously, the positive and significant correlation between teaching quality and autonomy support is of the same magnitude (*r* = .49, *k* = 11) as the correlation between teacher relationship quality and autonomy support. This indicates that autonomy-supportive teachers are rated as being of higher quality but may also suggest that perceptions of teacher quality are conflated with perceptions of relationship quality. Student-reported academic satisfaction was also positively and strongly related to autonomy support (*r* = .47, *k* = 16).

**Table 5.** Associations With Relationship Outcomes.

Covariates	*k*	*n*	*r*	95% Confidence interval		80% Credibility interval		*t* ^2^	*t*	I^2^
				Lower	Higher	Lower	Higher			
Positive relationship with teachers										
Autonomy support	6	4,806	.53	0.34	0.71	0.27	0.78	0.03	0.17	97.86
Positive relationship with parents										
Autonomy support	19	6,614	.36	0.29	0.43	0.18	0.54	0.02	0.14	89.47
Positive relationship with peers										
Autonomy support	11	8,400	.36	0.22	0.50	0.08	0.65	0.04	0.21	97.75
Teaching quality										
Autonomy support	11	20,905	.49	0.39	0.59	0.29	0.70	0.02	0.15	98.64
Academic satisfaction										
Autonomy support	16	7,179	.47	0.37	0.56	0.23	0.70	0.03	0.17	95.72

### Outcomes for Students

This area has attracted the most attention and is divided here into four subsequent parts focusing on learning outcomes, motivational outcomes, engagement-based behaviors, and student well-being. We begin by examining how supportive behaviors impact the learning outcomes of students (Table 6).

**Table 6.** Associations With Learning Outcomes.

Covariates	*k*	*n*	*r*	95% Confidence interval		80% Credibility interval		*t* ^2^	*t*	I^2^
				Lower	Higher	Lower	Higher			
Performance (general)										
Autonomy support	90	95,628	.27	0.24	0.30	0.07	0.46	0.02	0.15	96.49
Competence support	16	16,081	.05	–0.08	0.17	–0.27	0.36	0.06	0.24	98.24
Relatedness support	15	14,375	.17	0.09	0.26	–0.03	0.38	0.02	0.15	95.93
Autonomy thwarting	6	2,394	–.19	–0.28	–0.11	–0.29	–0.09	<0.01	0.07	66.33
Grade point average (GPA)										
Autonomy support	64	49,621	.11	0.07	0.14	–0.06	0.27	0.02	0.13	92.64
Competence support	12	10,717	.03	–0.03	0.08	–0.08	0.13	0.01	0.08	83.64
Relatedness support	13	10,318	.01	–0.04	0.07	–0.11	0.14	0.01	0.09	86.59
Autonomy thwarting	9	6,435	–.26	–0.44	–0.08	–0.58	0.07	0.05	0.23	97.78
Cognitive skills										
Autonomy support	22	7,304	.28	0.21	0.35	0.09	0.47	0.02	0.15	89.24
Creativity										
Autonomy support	4	950	.19	–0.09	0.47	–0.08	0.46	0.03	0.16	87.07

#### Learning Outcomes

**Figure 2.**
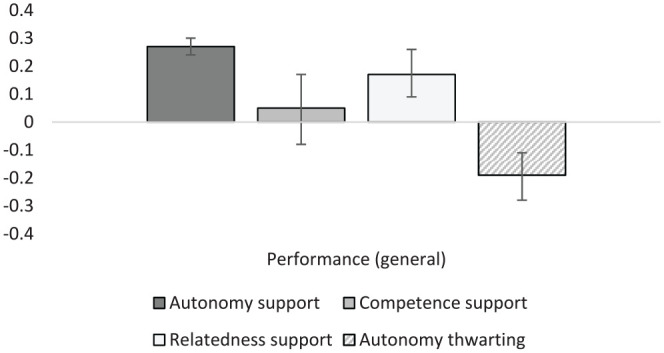
Graphical representation of performance (general) results.

**Figure 3.**
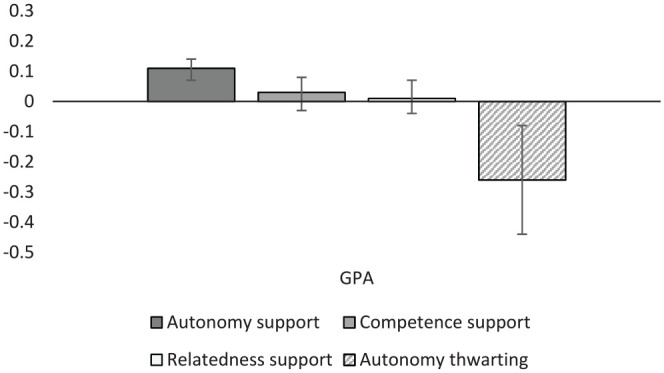
Graphical representation of grade point average (GPA) results.

Autonomy support was positively correlated with both general academic performance (*r* = .27, *k* = 90) and with the more specific outcome of GPA (*r* = .11, *k* = 64; Figures 2 and 3). Autonomy thwarting displayed the opposite pattern with significant negative correlations with general performance (*r* = –.19, *k* = 6) and GPA (*r* = –.26, *k* = 9), with results for autonomy thwarting slightly stronger when relating to GPA. Interestingly, competence support was unrelated to either measure of student performance (*r* = .04 and .02) despite reasonable sample sizes (*k* = 16 and 12, respectively). It appears that competence support, as often operationalized as “structure” in SDT studies, can have either positive or negative associations with student learning and performance, and on average is unlikely to be associated with these outcomes. Relatedness satisfaction displayed similar results with a very small and non-significant association with GPA (*r* = .01, *k* = 13), though a stronger and significant positive association with general performance (*r* = .17, *k* = 15). Autonomy support was also associated positively and significantly with cognitive skills (*r* = .28, *k* = 22), and creativity (*r* = .19, *k* = 4), suggesting that students who experience more autonomy support are more likely to be creative and stronger in a broad variety of specific cognitive skills (i.e., problem-solving tasks, Stroop tasks, and executive function tests).

When it comes to learning strategies students can employ (Table 7), it was found that autonomy support was positively correlated with the use of various learning strategies (*r* = .34, *k* = 12). More specifically, both autonomy support and competence support were significantly associated with deep learning practices (*r* = .33 and .28, respectively), whereas autonomy support was unrelated to surface learning strategies. Metacognitive learning strategies were positively associated with autonomy support (*r* = .40, *k* = 10) and negatively related to autonomy thwarting (*r* = –.42, *k* = 5). Both time management and goal setting/planning were positively correlated with autonomy support (*r* = .35 and .34, respectively). Results suggest that students who perceive more autonomy support are more likely to take ownership of their own learning and educational experience.

**Table 7.** Associations With Learning Strategies.

Covariates	*k*	*n*	*r*	95% Confidence interval		80% Credibility interval		*t* ^2^	*t*	I^2^
				Lower	Higher	Lower	Higher			
Learning strategy use										
Autonomy support	12	4,801	.34	0.27	0.41	0.21	0.48	0.01	0.10	83.90
Deep learning										
Autonomy support	16	10,833	.33	0.20	0.47	–0.01	0.68	0.07	0.26	98.24
Competence support	3	1,739	.29	0.03	0.54	0.11	0.47	0.01	0.10	86.21
Surface learning										
Autonomy support	5	2,371	.17	–0.04	0.37	–0.08	0.41	0.03	0.16	92.78
Metacognition										
Autonomy support	10	6,489	.40	0.33	0.46	0.29	0.50	0.01	0.08	84.95
Autonomy thwarting	5	5,754	–.42	–0.56	–0.28	–0.59	–0.25	0.01	0.11	95.45
Time management										
Autonomy support	10	3,971	.35	0.30	0.41	0.27	0.43	<0.01	0.06	64.71
Goal setting/planning										
Autonomy support	10	5,266	.34	0.25	0.43	0.18	0.50	0.01	0.12	90.03

#### Motivational Beliefs and Goal Orientation

When examining motivational beliefs of students (Table 8), it was found that autonomy support correlated positively with self-regulation (*r* = .16), whereas competence support, relatedness support, and autonomy thwarting were unrelated to the outcome. A similar finding was observed concerning the degree of personal control students reported within their educational context with autonomy support correlating positively (*r* = .31, *k* = 40), while relatedness support and autonomy thwarting were not significantly correlated (*k* = 6 and 8, respectively). Self-efficacy was strongly associated with autonomy support (*r* = .41, *k* = 42), competence support (*r* = .41, *k* = 7), and relatedness support (*r* = .59, *k* = 6), and unrelated to autonomy thwarting. Autonomy support also correlated positively with the value students attached to their educational experiences (*r* = .43, *k* = 28).

**Table 8.** Associations With Motivational Beliefs.

Covariates	*k*	*n*	*r*	95% Confidence interval		80% Credibility interval		*t* ^2^	*t*	I^2^
				Lower	Higher	Lower	Higher			
Self-regulation										
Autonomy support	26	13,163	.16	0.10	0.22	–0.03	0.36	0.02	0.15	92.01
Competence support	4	2,261	.23	–0.12	0.59	–0.13	0.60	0.05	0.22	96.87
Relatedness support	6	2,500	.11	–0.05	0.27	–0.10	0.33	0.02	0.14	89.82
Autonomy thwarting	8	4,954	–.16	–0.37	0.05	–0.52	0.20	0.06	0.25	97.65
Student perception of control										
Autonomy support	40	14,821	.31	0.25	0.36	0.10	0.51	0.03	0.16	91.95
Relatedness support	4	7,378	.15	–0.01	0.31	–0.01	0.31	0.01	0.10	94.78
Autonomy thwarting	4	1,482	.02	–0.14	0.19	–0.13	0.17	0.01	0.09	75.26
Self-efficacy										
Autonomy support	42	25,312	.41	0.36	0.45	0.21	0.60	0.02	0.15	95.23
Competence support	7	4,499	.41	0.28	0.53	0.21	0.60	0.02	0.13	94.21
Relatedness support	6	6,822	.59	0.38	0.81	0.29	0.89	0.04	0.20	99.11
Autonomy thwarting	4	1,881	.00	–0.31	0.31	–0.31	0.31	0.04	0.19	94.32
Valuation of education										
Autonomy support	28	12,309	.43	0.38	0.49	0.25	0.61	0.02	0.14	92.51
General performance goal orientation										
Autonomy support	7	2,458	.06	0.00	0.13	0.00	0.13	<0.01	0.04	41.15
Performance approach										
Autonomy support	14	4,960	.14	0.04	0.25	–0.09	0.38	0.03	0.17	91.53
Performance avoidance										
Autonomy support	12	4,252	–.03	–0.14	0.09	–0.26	0.21	0.03	0.17	91.14
General mastery goal orientation										
Autonomy support	16	13,199	.24	0.16	0.32	0.04	0.44	0.02	0.15	95.34
Mastery approach										
Autonomy support	15	6,211	.32	0.25	0.40	0.15	0.50	0.02	0.13	89.78
Mastery avoidance										
Autonomy support	8	2,675	–.03	–0.32	0.26	–0.51	0.46	0.12	0.34	97.48

Data were available on the correlations between performance and mastery orientation with autonomy support, but none of the remaining forms of support. Results indicate that on the whole, autonomy support was more strongly associated with mastery goals than performance goals (*r* = .23, *k* = 16 compared with *r* = .06, *k* = 7). Digging deeper into goal orientation, it is also evident that autonomy support correlated more strongly with approach goal orientations than avoidance goal orientations, with a significant positive association with performance approach (*r* = .14, *k* = 14) and a non-significant association with performance avoidance. Likewise, autonomy support correlated with mastery approach (*r* = .32, *k* = 15) while being unrelated to mastery avoidance goal orientation. In line with theory, it appears autonomy support is related to mastery over performance goal orientations, and also with approach rather than avoidance goal orientations.

#### Engagement

**Figure 4.**
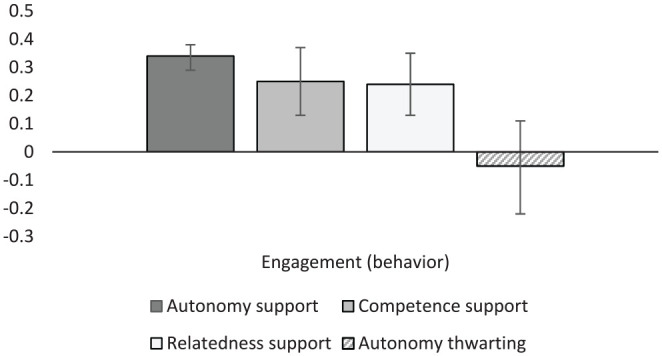
Graphical representation of engagement (behavior) results.

**Figure 5.**
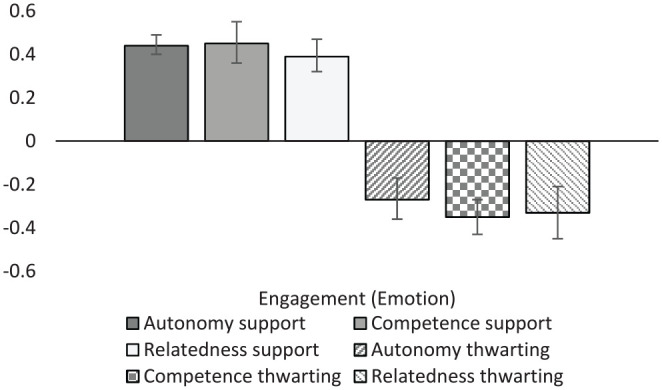
Graphical representation of engagement (emotional) results.

Student engagement was examined via several composite variables. Engagement and disengagement were broken down into emotional, behavioral, cognitive, and state engagement constructs (Table 9; Figures 4 and 5). Several specific engagement behaviors (proactive, prosocial, and persistence behaviors), misconduct behaviors, and coping strategies were also extracted from the literature and analyzed individually (see Supplemental Table S1). General (dis)engagement variables were also created to capture forms of engagement not captured with the more specific categories (i.e., when the reported outcome was “engagement”).

**Table 9.** Associations With Engagement Outcomes.

Covariates	*k*	*n*	*r*	95% Confidence interval		80% Credibility interval		*t* ^2^	*t*	I^2^
				Lower	Higher	Lower	Higher			
Disengagement (general)										
Autonomy support	5	3,720	–.38	–0.55	–0.21	–0.59	–0.18	0.02	0.13	94.84
Disengagement (emotional)										
Autonomy support	12	5,731	–.33	–0.41	–0.25	–0.49	–0.17	0.01	0.12	89.17
Disengagement (behavior)										
Autonomy support	6	4,109	–.26	–0.35	–0.17	–0.37	–0.15	0.01	0.07	80.75
Engagement (general)										
Autonomy support	53	34,417	.37	0.30	0.44	0.06	0.68	0.06	0.24	98.05
Competence support	8	2,121	.44	0.24	0.63	0.12	0.75	0.05	0.22	95.22
Relatedness support	7	9,856	.33	0.21	0.46	0.15	0.52	0.02	0.13	96.79
Engagement (behavior)										
Autonomy support	57	33,227	.34	0.29	0.38	0.12	0.56	0.03	0.17	95.53
Competence support	17	12,554	.25	0.13	0.37	–0.06	0.55	0.05	0.23	97.73
Relatedness support	10	8,290	.24	0.13	0.35	0.04	0.44	0.02	0.15	95.22
Autonomy thwarting	6	5,245	–.05	–0.22	0.11	–0.28	0.17	0.02	0.15	95.34
Engagement (emotional)										
Autonomy support	61	26,842	.44	0.40	0.49	0.22	0.67	0.03	0.17	95.25
Competence support	14	9,513	.45	0.36	0.55	0.24	0.66	0.02	0.16	96.28
Relatedness support	14	7,193	.39	0.32	0.47	0.22	0.56	0.02	0.12	91.73
Autonomy thwarting	5	3,654	–.27	–0.36	–0.17	–0.37	–0.17	<0.01	0.07	78.27
Competence thwarting	3	2,950	–.35	–0.43	–0.27	–0.38	–0.32	<0.01	0.02	27.29
Relatedness thwarting	4	4,244	–.33	–0.45	–0.21	–0.44	–0.21	<0.01	0.07	86.38
Engagement (cognitive)										
Autonomy support	25	15,594	.39	0.31	0.47	0.15	0.63	0.03	0.18	96.58
Competence support	6	4,567	.36	0.16	0.55	0.09	0.63	0.03	0.18	97.12
Engagement (state)										
Autonomy support	37	12,486	.43	0.39	0.47	0.28	0.58	0.01	0.11	87.02
Engagement (time)										
Autonomy support	7	4,147	–.05	–0.12	0.03	–0.15	0.05	<0.01	0.07	73.47
Intention to act										
Autonomy support	31	13,609	.35	0.29	0.40	0.16	0.54	0.02	0.14	92.12
Proactivity										
Autonomy support	18	9,290	.44	0.35	0.53	0.20	0.68	0.03	0.18	96.10
Persistence										
Autonomy support	14	10,424	.25	0.20	0.30	0.13	0.37	0.01	0.09	86.18
Competence support	7	6,667	.35	0.31	0.38	0.32	0.38	<0.01	0.02	38.66
Relatedness support	6	2,449	.26	0.13	0.40	0.09	0.44	0.01	0.12	86.80
Prosocial behaviors										
Autonomy support	24	12,018	.23	0.17	0.28	0.05	0.40	0.02	0.13	90.33

Disengagement in all its forms was negatively associated with autonomy support (*r* = –.26 to –.38). Unfortunately, there was not enough data to examine correlations between disengagement and other forms of support and thwarting. General engagement was positively related to autonomy support (*r* = .37), competence support (*r* = .44), and relatedness support (*r* = .33). Interestingly, data were available for three separate types of thwarting behaviors with autonomy thwarting (*r* = –.27, *k* = 5), competence thwarting (*r* = –.35, *k* = 3), and relatedness thwarting (*r* = –.33, *k* = 4) each correlating significantly and negatively with general engagement. Emotional engagement correlated positively and strongly with supportive behaviors, ranging from .39 to .45. Supportive behaviors were significantly, but less strongly associated with behavioral engagement (*r_M_* = .27). It is also worth noting that autonomy thwarting was unrelated to behavioral engagement, potentially indicating that while thwarting contexts may diminish emotional engagement, they may not diminish behavioral engagement. Both autonomy support and competence support were positively correlated with cognitive engagement (*r* = .39 and .35, respectively). Likewise, engagement state (primarily characterized by vigor, dedication, and absorption; Schaufeli et al., 2002) was positively associated with autonomy support (*r* = .43, *k* = 37), as was students reported intention to engage in learning behaviors (*r* = .34, *k* = 31).

Autonomy support was positively correlated with proactive (*r* = .44), prosocial (*r* = .23), and persistence (*r* = .25) behaviors. Competence support was a significantly stronger correlate of persistence (*r* = .35) and displayed very little variance in effects. Relatedness support was a more modest correlate of persistence (*r* = .26). Autonomy support did not significantly correlate with the amount of time students reported spending on school-related activities.

**Figure 6.**
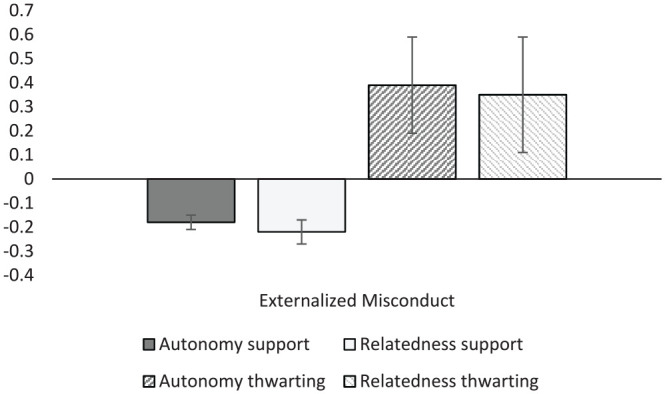
Graphical representation of externalized misconduct results.

Misconduct and coping variables were examined next (Table 10; Figure 6). General externalized misconduct was negatively associated with both autonomy and relatedness support (*r* = –.18 and –.22, respectively) and more strongly and positively correlated with autonomy thwarting and relatedness-thwarting contexts (*r* = .39 and .35). Autonomy support correlated negatively with school dropout intentions (*r* = –.21, *k* = 7), student absenteeism (*r* = –.15, *k* = 7), as well as procrastination (*r* = –.22, *k* = 7). General negative coping strategies were also negatively related to autonomy support (*r* = -.24) and positively related to autonomy thwarting (*r* = .43).

**Table 10.** Associations With Misconduct and Coping Outcomes.

Covariates	*k*	*n*	*r*	95% Confidence interval		80% Credibility interval		*t* ^2^	*t*	I^2^
				Lower	Higher	Lower	Higher			
Externalized misconduct										
Autonomy support	47	22,190	–.18	–0.21	–0.15	–0.31	–0.05	0.01	0.10	83.35
Relatedness support	6	3,136	–.22	–0.27	–0.17	–0.24	–0.20	<0.01	0.01	10.35
Autonomy thwarting	7	4,620	.39	0.19	0.59	0.09	0.70	0.05	0.21	97.69
Relatedness thwarting	4	3,416	.35	0.11	0.59	0.11	0.60	0.02	0.15	96.10
Procrastination										
Autonomy support	7	4,028	–.22	–0.32	–0.13	–0.36	–0.09	0.01	0.09	84.74
Absenteeism										
Autonomy support	7	2,241	–.15	–0.21	–0.08	–0.20	–0.09	<0.01	0.04	35.10
Dropout										
Autonomy support	7	5,595	–.21	–0.33	–0.08	–0.39	–0.02	0.02	0.13	93.66
Negative coping strategies										
Autonomy support	10	3,443	–.24	–0.29	–0.19	–0.32	–0.16	<0.01	0.06	54.62
Autonomy thwarting	4	2,212	.43	0.26	0.61	0.26	0.60	0.01	0.10	89.87
Positive coping strategies										
Autonomy support	9	3,116	.20	0.13	0.27	0.10	0.31	0.01	0.08	67.98
Emotional regulation										
Autonomy support	10	3,822	.21	0.10	0.31	0.02	0.39	0.02	0.13	87.72
Interpersonal skills										
Autonomy support	14	4,890	.40	0.32	0.48	0.23	0.57	0.02	0.13	88.68
Identity development										
Autonomy support	5	3,160	.23	0.03	0.43	–0.01	0.47	0.02	0.16	94.43

In contrast to these findings, emotional regulation and positive coping strategies correlated positively with autonomy support (*r* = .21 and .20). Students who reported receiving greater autonomy support also reported stronger identity development (*r* = .23, *k* = 5) as well as more developed interpersonal skills (*r* = .40, *k* = 14). These results may indicate that students in autonomy-supportive environments develop and are permitted to use social skills more effectively to regulate their emotions, partially avoiding the need for misconduct.

#### Well-Being

**Figure 7.**
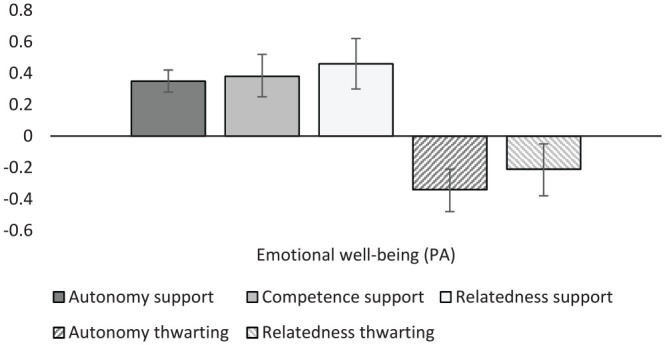
Graphical representation of well-being (PA) results. Note. PA = positive affect.

**Figure 8.**
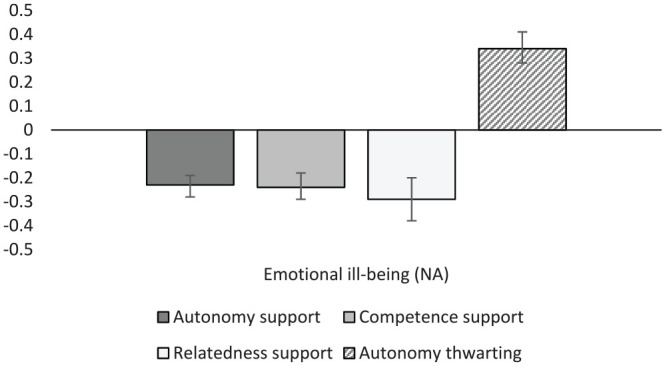
Graphical representation of ill-being (NA) results. *Note.* NA = negative affect.

The final meta-analytic results to be presented are those for student well-being and health (Table 11; Figures 7 and 8). Autonomy support and relatedness support were significantly correlated with general student well-being (*r* = .38 and .26, respectively), while competence support was not. All three were associated with eudemonic well-being (i.e., life satisfaction and meaningfulness of life; *r* = .38, .36, and .42) and emotional well-being (*r* = .35, .38, and .46). Autonomy thwarting demonstrated significant negative associations with emotional well-being (*r* = –.34), while relatedness thwarting was found to have a similar though weaker association (*r* = –.21). As one would expect, general ill-being showed the opposite pattern of effects, displaying moderate negative correlations with autonomy support (*r* = –.14) and relatedness support (*r* = –.21), and positive associations with autonomy thwarting (*r* = .28) and relatedness thwarting (*r* = .22). Likewise, emotional ill-being was negatively correlated with autonomy support, competence support, and relatedness support (*r* = –.23, –.24, and –.29, respectively), while autonomy thwarting was significantly and positively correlated with emotional ill-being (*r* = .34).

**Table 11.** Associations With Well-Being and Health Outcomes.

Covariates	*k*	*n*	*r*	95% Confidence interval		80% Credibility interval		*t* ^2^	*t*	I^2^
				Lower	Higher	Lower	Higher			
Well-being (general)										
Autonomy support	47	22,000	.38	0.34	0.42	0.19	0.57	0.02	0.15	93.11
Competence support	4	2,348	.21	–0.17	0.59	–0.18	0.60	0.06	0.24	97.30
Relatedness support	9	10,725	.26	0.20	0.33	0.15	0.38	0.01	0.08	90.02
Emotional well-being (PA)										
Autonomy support	40	18,669	.35	0.28	0.42	0.08	0.62	0.04	0.21	96.20
Competence support	4	1,606	.38	0.25	0.52	0.27	0.50	0.00	0.07	73.18
Relatedness support	5	1,667	.46	0.30	0.62	0.27	0.65	0.02	0.12	89.16
Autonomy thwarting	9	4,085	–.34	–0.48	–0.21	–0.58	–0.11	0.03	0.17	94.10
Relatedness thwarting	6	3,840	–.21	–0.38	–0.05	–0.44	0.02	0.02	0.15	93.84
Eudemonic well-being										
Autonomy support	30	15,168	.38	0.33	0.43	0.22	0.55	0.02	0.13	91.73
Competence support	4	1,243	.36	0.28	0.44	0.34	0.39	<0.01	0.01	7.77
Relatedness support	6	1,676	.42	0.27	0.58	0.22	0.63	0.02	0.14	88.62
Self-esteem										
Autonomy support	42	24,415	.22	0.18	0.26	0.06	0.38	0.02	0.12	90.58
Competence support	5	8,158	.11	–0.06	0.27	–0.09	0.30	0.02	0.13	96.52
Relatedness support	8	8,915	.17	0.05	0.28	–0.02	0.35	0.02	0.13	95.29
General ill-being										
Autonomy support	30	15,955	–.14	–0.21	–0.07	–0.37	0.09	0.03	0.17	94.24
Relatedness support	8	79,33	–.21	–0.26	–0.16	–0.28	–0.14	<0.01	0.05	70.97
Autonomy thwarting	4	2,061	.28	0.06	0.51	0.06	0.51	0.02	0.14	91.93
Relatedness thwarting	5	3,728	.22	0.02	0.41	–0.02	0.45	0.02	0.15	93.86
Emotional ill-being (NA)										
Autonomy support	61	28,422	–.23	–0.28	–0.19	–0.45	–0.02	0.03	0.16	93.31
Competence support	6	1,997	–.24	–0.29	–0.18	–0.26	–0.21	<0.01	0.02	8.77
Relatedness support	11	4,680	–.29	–0.38	–0.20	–0.46	–0.13	0.01	0.12	88.10
Autonomy thwarting	10	6,285	.34	0.28	0.41	0.23	0.46	0.01	0.09	85.36
Anxiety										
Autonomy support	26	10,567	–.20	–0.25	–0.15	–0.35	–0.04	0.01	0.12	85.95
Competence support	5	1,638	–.13	–0.28	0.02	–0.29	0.04	0.01	0.11	79.64
Relatedness support	6	1,252	–.26	–0.36	–0.15	–0.37	–0.15	0.01	0.08	58.04
Depression										
Autonomy support	37	22,014	–.21	–0.25	–0.17	–0.37	–0.05	0.01	0.12	90.36
Relatedness support	7	7,061	–.26	–0.31	–0.20	–0.33	–0.18	<0.01	0.05	75.21
Physical activity intention										
Autonomy support	26	13,129	.30	0.24	0.35	0.14	0.45	0.01	0.12	89.85
Competence support	4	2,047	.33	0.22	0.44	0.24	0.43	0.00	0.06	67.56
Autonomy thwarting	5	2,370	–.21	–0.52	0.11	–0.58	0.17	0.06	0.25	96.91
Healthy lifestyle										
Autonomy support	40	16,294	.22	0.19	0.26	0.09	0.36	0.01	0.10	83.12
Autonomy thwarting	7	3,032	–.11	–0.30	0.08	–0.40	0.18	0.04	0.20	94.80
General health										
Autonomy support	19	14,169	.15	0.12	0.19	0.06	0.24	<0.01	0.07	78.74

*Note.* PA = positive affect; NA = negative affect.

When looking at anxiety and depression as specific instances of ill-being it was noticed that autonomy support (*r* = –.20 and –.21 for anxiety and depression, respectively) and relatedness support (*r* = –.26 and –.26) were both significantly correlated to the outcomes, though relatedness support estimates contained less heterogeneity. Autonomy support and relatedness support were also significantly correlated with self-esteem, whereas competence support was not. Very little difference was observed between estimated effects associated with autonomy and relatedness categories in relation to well-being variables. It is also worth noting that in relation to well-being outcomes, competence support has been studied much less frequently than other forms of support.

Autonomy support was significantly and positively associated with general health (*r* = .15, *k* = 19). Students reporting higher autonomy support also reported a healthier lifestyle (including better eating and exercise habits; *r* = .22, *k* = 40), whereas autonomy thwarting was unrelated to this outcome. This same pattern was noticed when examining the intention to participate in physical activity in which autonomy and competence support were both positively associated with the outcome (*r* = .30 and .33, respectively) and autonomy thwarting was not significantly correlated.

#### Relative Weights Analysis

RWA was conducted for eight outcomes, with each being predicted by at least four types of support/thwarting behaviors (Table 12). Results show that self-regulation was most closely associated with competence support, whereas self-efficacy was primarily a function of relatedness support. Engagement behaviors were primarily associated with perceived autonomy support, and surprisingly, GPA was estimated to be associated primarily with autonomy thwarting (a negative association). Emotional engagement and emotional well-being were both equally predicted by a range of supporting and thwarting behaviors. Emotional ill-being and misconduct were primarily associated with autonomy thwarting and relationship thwarting respectively, aligning with the dual pathway hypothesis of SDT.

**Table 12.** Relative Weights Analysis of Available Outcomes.

Outcome	*R* ^2^	Autonomy support		Competence support		Relatedness support		Autonomy thwarting		Competence thwarting		Relatedness thwarting	
		β	%	β	%	β	%	β	%	β	%	β	%
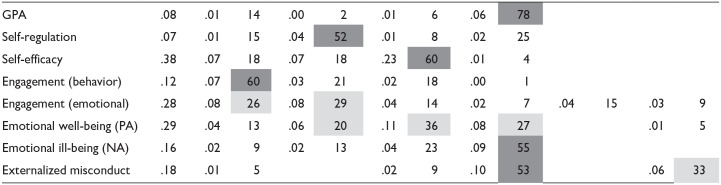													

*Note.* Most notable results are shaded in gray. GPA = grade point average; PA = positive affect; NA = negative affect.

## Discussion

We sought to review need-supportive and need-thwarting student experiences from the perspective of SDT. Data derived from 638 samples were analyzed and 72 covariates of supportive and thwarting behaviors were examined. We addressed five research questions relating to the (a) interconnection between support and thwarting behaviors, (b) antecedents of support, (c) implications for the people providing the support, (d) implications for the students being supported, and (e) the relative contribution of each type of need-supportive and need-thwarting behaviors.

Beginning with the simple correlations between support and thwarting variables, we noticed significant overlap. The three support variables were highly correlated (mean *r* = .66), and likewise, the three thwarting variables were similarly correlated (mean *r* = .59). This is not entirely unexpected by SDT, since socializing agents who support (or thwart) one need, are also more likely enact similar tendencies toward other needs (Deci et al., 2017). It is also argued within SDT that satisfaction of one psychological need can facilitate the satisfaction of other needs as well (Ryan & Deci, 2017), meaning the mechanisms through which need-support and need-thwarting impact outcomes are interconnected. This will result in support and thwarting categories that appear to relate to covariates in similar ways, despite theoretical differences. However, if support and thwarting categories correlate consistently and strongly, and effectively achieve the same results in promoting all basic needs, the distinction between them becomes questionable, the constructs potentially redundant, and separate scale unnecessary. As such, the nature of this overlap needs to be considered closely.

To begin exploring which specific behaviors may have cross-correlations, we provided preliminary analyses connecting the more specific supporting and thwarting behaviors with the broader constructs of autonomy support and competence support. There were significant positive correlations observed between all specific supportive behaviors and the broader constructs of autonomy and competence support, and likewise, almost all specific thwarting behaviors correlated negatively with both autonomy and competence support. However, a few specific behaviors displayed more differentiated results. Specifically, chaos (*r* = –.07) and helicopter parenting (*r* = –.05) both observed non-significant associations with autonomy support, whereas chaos correlated significantly and negatively with competence support (*r* = –.20), indicating that a chaotic environment appears largely orthogonal to autonomy support, but detrimental to competence support. These findings are theoretically justified (Aelterman et al., 2019) and appear to represent behaviors with well-differentiated functions. The warmth behavior, on the contrary, was the strongest correlate of both autonomy support (*r* = .57) and competence support (*r* = .61) composites, indicating a significant degree of covariation. This may indicate a confound that could be further explored and better defined in measures.

### Autonomy Support

When examining autonomy support and its covariates, it was consistently noted to have positive associations with a range of desirable student outcomes. Students who reported higher levels of autonomy support appear to attain better results in their academic studies and apply a broader range of learning strategies. Supported students also reported greater engagement in class, including more prosocial and proactive behaviors, greater persistence with school tasks, and higher levels of emotional, cognitive, and behavioral engagement. They also experience greater well-being, both in terms of positive emotions, life satisfaction, and more eudemonic aspects of well-being, such as meaningfulness in life, and lower levels of disengagement. Supported students also tended to possess more extroverted, open, agreeable, conscientious, and less neurotic personality traits (La Guardia & Ryan, 2007; Sheldon et al., 1997). In the relative weight analysis, autonomy support was also predictive of reduced student ill-being and misconduct. As expected by SDT, autonomy support appears to be universally desirable.

### Competence and Relatedness Support

Competence and relatedness support were generally associated with a similar pattern of consistently desirable results for students. Our meta-analysis allowed for direct comparisons between competence support and autonomy support across 21 variables and found that effect sizes with covariates were indistinguishable across at least 14 of these. Only three covariates had clearly distinguishable effect sizes. Competence support emerged as a stronger correlate of persistence. However, the key difference was that, interestingly, competence support was not associated with performance (either GPA or general educational performance). Given the variance associated with this finding, it appears that structure can at times be detrimental and at other times beneficial to student performance in school. It may be that structure needs to be accompanied by autonomy support to be helpful (Jang et al., 2010), or alternately other moderating factors may be at play. However, because competence support has been examined relatively few times across different variables, more precise inquiry was not possible. RWA indicated that competence support was particularly important in predicting one of the eight outcomes, specifically self-regulation. As such, the results indicate that competence support provides only minor incremental validity, at least with some covariates.

A similar though more differentiated pattern is noted when examining relatedness support and autonomy support with 11 of the 20 comparisons returning near-identical correlational coefficients. Relatedness support was a stronger positive correlate with self-efficacy and was also a stronger negative correlate of general ill-being. Relatedness support also appears to be a stronger negative correlate of externalized misconduct. Relatedness support was, however, a weaker associate of perceived student control, and performance (general performance and GPA), with the correlation between relatedness support and GPA being non-significant despite a reasonable sample size and narrow confidence intervals. RWA further indicated that relatedness support is particularly important in relation to student self-efficacy, and potentially emotional well-being. These results indicate that relatedness support also shares substantial overlap with autonomy support, yet may have a more unique role to play when considering outcomes such as ill-being and misbehavior.

### Need Thwarting

Need thwarting is considered distinct from need support and not merely its inverse (Bartholomew, Ntoumanis, Ryan, Bosch, & Thøgersen-Ntoumani, 2011; Bartholomew, Ntoumanis, Ryan, & Thøgersen-Ntoumani, 2011). It is also proposed to capture variance in undesirable outcomes to a greater extent than need support (e.g., Jang et al., 2016). This is termed the dual-path model in SDT (Vansteenkiste et al., 2020) and results from this meta-analysis can help shed light on this recent theoretical advancement in the literature. Results not only partially support this theory but also partially support the idea of it being a polar opposite of need support. For instance, the observed relationship from autonomy support and autonomy thwarting to several covariates were identical, though oppositely valenced, including emotional engagement, use of metacognitive strategies, and both general performance and GPA. In these instances, the correlations indicate opposing rather than differentiated factors. However, in other instances, autonomy support and thwarting demonstrated different correlations with outcomes (i.e., with engagement behaviors, self-efficacy, perceived control, and self-regulation). These findings support the logic of the dual-path model. RWA indicates clearer evidence for the dual pathway model with the two negatively valenced outcomes (emotional ill-being and externalized misconduct) predicted primarily by autonomy-thwarting behaviors. Accordingly, the results indicate that while autonomy thwarting may indeed capture some unique variance particularly relating to negatively valenced covariates, scales measuring it continue to correlate highly and should endeavor to distinguish themselves from supportive counterparts to the greatest extent possible.

### Implications for Supporters

Finally, there is a relative lack of research on predictors of supportive and thwarting teaching, and likewise on the implications for support providers. Research has shown that teachers and parents who themselves experience satisfaction for basic psychological needs (and personal well-being) are more likely to support the basic psychological needs of followers (Aelterman et al., 2014; Reeve & Cheon, 2021), with the calculated correlation in this study being of moderate to strong magnitude. The education level attained by teachers and parents also positively related to support provision. Likewise, further research might examine what benefits teachers and other supporters receive from support provision (if any). Results presented here indicate that providing support is likely to build stronger relationships between the agents providing the support and students receiving support and that need-supportive teachers are likely to be rated as more effective teachers. Other experimental research has also indicated positive motivational and well-being benefits to teachers (Cheon et al., 2014). However, many other potential antecedents, such as parent demographics and personality, student minority background, or cognitive abilities remain relatively under-explored. Given the comprehensive knowledge the field has developed on the student outcomes of support, the next major question for the field may be how best to encourage teachers and parents to provide support to students (e.g., Cheon et al., 2014, 2020), and consideration of the outcomes for support providers may be a necessary consideration in this discussion.

### Limitations and Directions for Future Research

First, we must acknowledge the correlational nature of the data included in this meta-analysis. While the positioning of variables as either antecedents or outcomes is consistent with theory and existing non-correlational research (e.g., Gillison et al., 2019; Ntoumanis et al., 2021), our estimates are not indicative of causal relationships. It could be that variables typically modeled as outcomes of need-supportive behaviors could also play a role as antecedents to those behaviors. Indeed, much of the SDT literature assumes a unidirectional process in which educator behaviors influence student outcomes. However, many phenomena are likely to involve simultaneous influence processes in which causality runs both ways, creating a possible simultaneity bias and, in turn, endogeneity (Güntner et al., 2020). As an example, our meta-analysis showed that need-supportive behaviors are generally positively associated with student achievement. While it is typically assumed that supportive behaviors will precede performance, it is also likely that students’ academic competence will prompt particular styles of behavior in their teachers or parents (e.g., Grolnick et al., 2002; Reeve, 2012). Future work examining the direction of causality, and potential reciprocal effects will be important for both theory development and practice.

We were unable to estimate correlations between different specific behaviors within the broader support and thwarting categories (e.g., correlations between chaos, warmth, involvement, and feedback). This may indicate a weakness of our search procedure in which we excluded terms such as “structure” “involvement” or “chaos.” Alternately, it may be because research typically relies upon broader and encompassing variables such as “autonomy support.” More research into the specific behaviors that comprise support and thwarting in educational contexts would be highly useful (see Ahmadi et al., 2023; Teixeira et al., 2020 for excellent examples). This recommendation aligns with that of Mageau and Joussemet (2023) who highlight the lack of specificity and consistency of autonomy-supportive behaviors being measured. Such a taxonomy will be practically useful in guiding how to enact supportive behaviors (Teixeira et al., 2020), and to the extent that more refined behaviors are built into measures, will also help distinguish the support/thwarting categories further in empirical research.

With our focus on thwarting behaviors and other less researched forms of support, the sample sizes were often not large enough to conduct meaningful moderation analyses. Particularly interesting would be a comparison of support/thwarting from teachers and parents as we might expect teachers to be more proximal, and therefore stronger influencers on student outcomes (Bureau et al., 2022). As such, the reported estimates contain a degree of heterogeneity (I^2^) that may be explained by various moderating factors including nationality, the scales used, the school subject being studied, or individual differences of participants. Further moderation analyses may be insightful when relevant research questions arise.

Looking outside of SDT, we offer two broader directions for research. The first concerns the similarity between the taxonomy of supporting behaviors developed in SDT and neighboring fields of research. For example, we might look at leadership research from the management discipline. Both of these fields are interested in how a teacher/leader can influence a group of students/employees to achieve performance goals while maintaining well-being. In this respect, teachers and organizational leaders are functionally similar. Research in management support this claim with taxonomies of leadership behaviors aligning closely with SDT with a focus on task-focused (competence-supporting) behaviors and relationship-focused (relatedness-supporting) behaviors (e.g., Yukl et al., 2002), and more recently recognizing the importance of empowering employees (i.e., providing them with autonomy). Other areas of educational research should also be considered as extensive research has been conducted on the student-teacher relationship (Endedijk et al., 2022), attachment (García-Rodríguez et al., 2022), and deliverance of feedback (Hattie & Timperley, 2007), for example. It is promising to see SDT scholars beginning to bridge the gap (Aelterman et al., 2019; Van den Broeck & Slemp, 2023); however, a deeper consideration of these fields may prove mutually beneficial as knowledge and expertise are shared more freely.

A final direction is to take this accumulated knowledge into the domain of public policy. This field has a very strong base of evidence to support the importance of autonomy-supportive behaviors, including a well-developed line of intervention research (Su & Reeve, 2011; also see Ntoumanis et al., 2021). This application has had significant benefits to the schools and teachers that are subject to the intervention, and autonomy-supportive practices are now more widely taught and practiced. However, larger institutional issues remain. For example, Ryan and Weinstein (2009) wrote convincingly about the controlling pressures forced upon schools, teachers, and subsequently students, by high-stakes testing. This remains an issue and may even be increasing as schools continue to compete for prestige and funding. Issues such as this point to the importance of institutional change in the form of educational policy reform. This is no small task but remains important nonetheless.

## Supplemental Material

sj-docx-1-psp-10.1177_01461672231225364 – Supplemental material for Need Support and Need Thwarting: A Meta-Analysis of Autonomy, Competence, and Relatedness Supportive and Thwarting Behaviors in Student Populations Supplemental material, sj-docx-1-psp-10.1177_01461672231225364 for Need Support and Need Thwarting: A Meta-Analysis of Autonomy, Competence, and Relatedness Supportive and Thwarting Behaviors in Student Populations by Joshua L. Howard, Gavin R. Slemp and Xiao Wang in Personality and Social Psychology Bulletin
