# Supplementary material for: Need Support and Need Thwarting: A Meta-Analysis of Autonomy, Competence, and Relatedness Supportive and Thwarting Behaviors in Student Populations
Source: Pers Soc Psychol Bull. 2024 Jan 30;51(9):1552–73. doi: 10.1177/01461672231225364 (PMC12276404; doi:10.1177/01461672231225364)
Supplement: sj-docx-1-psp-10.1177_01461672231225364 – Supplemental material for Need Support and Need Thwarting: A Meta-Analysis of Autonomy, Competence, and Relatedness Supportive and Thwarting Behaviors in Student Populations [file sj-docx-1-psp-10.1177_01461672231225364.docx]

**Online Supplemental Materials for:**

Need Support and Need Thwarting: A Meta-Analysis of Autonomy, Competence, and Relatedness Supportive and Thwarting Behaviors in Student Populations

**Authors’ note:**

We developed these materials to provide additional technical information and to keep the main manuscript from becoming needlessly long. These online appendices are freely available via OSF and linked to the manuscript.

We would also be happy to have some of these materials brought back into the main manuscript or included as published appendices if you deem it useful.

Table S1. Record of Variable Categorization.

| **Broad category** | **Category analyzed in meta-analysis** | **Specific variables as measured in primary studies** |
| --- | --- | --- |
| Learning outcomes | Performance (general) | Performance, knowledge, School-related functioning, personal accomplishment, achievement, perceived learning, evaluation, goal progress, learning gains, completion, scholarship attainment, language skills, reading skills, reading scores, reading comprehension, science skills, homework quality, ability in various school subjects |
|  | GPA | GPA (self-reported and objectively rated), grades for a school year, semester, or term |
|  | Cognitive skills | This category includes achievement on a range of specific cognitive tests. Includes: Stroop test, number task, spatial location task, Bayley mental development index, Woodcock-Johnson applied problems, cognitive skill and executive functioning tests |
|  | Creativity | Creative thinking, creativity, flexibility, originality |
|  |  |  |
| Learning strategies | Learning strategy use | Learning strategy use, peer learning, learning strategies, optimal learning (composite) |
|  | deep learning | deep-level learning strategies, deep approach, deep learning, critical thinking, elaboration |
|  | surface learning | surface learning strategies, surface approaches, memorization, rehearsal |
|  | metacognition | metacognitive strategy use, metacognitions |
|  | time management | Time management, planning time, time and environment use |
|  | goal setting / planning | planning goal planning, career planning, lack of planning (reverse coded) |
|  |  |  |
| Motivational beliefs | Self-regulation | Self-regulation (cognitive and behavioral), effort regulation, delay of gratification, rule compliance, self-control, impulse control |
|  | student perception of control | perceived behavioral control, maladaptive control beliefs (reverse scored), control of learning beliefs |
|  | self-efficacy | self-efficacy, academic self-efficacy, improved expectancy, lack of efficacy (reverse coded) |
|  | valuation of education | Value beliefs, task value, value of specific classes (e.g. physical education or math), school value, attainment importance, social utility value, personal utility value, |
|  | General performance goal orientation | Grade orientation, performance orientation |
|  | performance approach | performance-approach goals |
|  | performance avoidance | performance-avoidance goals |
|  | General master goal orientation | Mastery orientation, mastery goals, homework mastery goals, |
|  | mastery approach | mastery approach goals |
|  | mastery avoidance | mastery-avoidance goals |
|  |  |  |
| Engagement | Disengagement (General) | Disengagement |
|  | Disengagement (emotional) | Disaffection, boredom, mind wandering |
|  | Disengagement (behavioral) | behavioral disengagement, agentic disengagement, passive bystanding |
|  | Engagement (general) | Engagement (self, teacher, and observer reported), willingness to communicate, learning engagement |
|  | Engagement (behavior) | Academic engagement, effort, compliance, homework effort, involvement in class |
|  | Engagement (emotional) | engagement - emotional, enjoyment, interest, interest in reading, interest-focused engagement |
|  | Engagement (cognitive) | engagement - cognitive, concentration, |
|  | Engagement (state) | Vigor, dedication, absorption, flow, vitality, harmonious passion, flourishing |
|  | Engagement (time) | Hours spent studying, practice time, self-study time |
|  | Intention to act |  |
|  | Proactivity | Agentic engagement, proactivity, participation, curiosity, participation & preparation |
|  | Persistence | Persistence intentions, persistence |
|  | Prosocial behaviors | Prosocial behavior, prosocial engagement, responsible behavior, other-oriented helping, ability to support, community behavior |
|  |  |  |
| Misconduct and coping | Externalized misconduct | Externalizing problems, problem behavior, misconduct, classroom violence, aggression, antisocial behavior, bullying, pro-bullying attitudes, oppositional defiance, acting-out |
|  | Procrastination | Procrastination, decisional procrastination, lack of functionality |
|  | Absenteeism | Absences, absenteeism, classes missed, skipping school |
|  | Drop out | Dropout, intentions to dropout, dropout risk, intention to persist at school (reverse coded) |
|  | Negative coping strategies | Escape, avoidance, self-handicapping, fear of failure, attention-seeking, reactance, rumination |
|  | Positive coping strategies | Social adjustment, personal-emotional adjustment, resist peer pressure, positive coping strategy |
|  | Emotional regulation | Emotion regulation, emotional integration, difficulties in emotion regulation (reverse coded), anger control, emotion suppression, |
|  | Interpersonal skills | Social skills, teamwork, communication skills, social cooperation, leadership, emotional skills |
|  | Identity development | Self-exploration, vocational exploration, commitment making, exploration in depth |
|  |  |  |
| Wellbeing | Wellbeing (general) | Wellbeing, general well-being, social wellbeing, psychological wellbeing, |
|  | Emotional wellbeing | Positive affect, positive emotions, hope, joy, |
|  | Eudemonic wellbeing | Life satisfaction, purpose in life, meaning in life, self-actualization |
|  | self-esteem | Self-esteem, self-concept, self-worth |
|  | General illbeing | Illbeing, internalizing symptoms, internalizing problems |
|  | Emotional illbeing | Negative affect, stress, cynicism, emotional exhaustion, loneliness, burnout, psychological distress |
|  | Anxiety | Anxiety |
|  | Depression | Depression, dysphoria |
|  | Physical activity intention | Physical activity intention, exercise intentions |
|  | Healthy lifestyle | Physical activity behavior, sport injury prevention behavior, average steps/day, sports practice, dietary self-care, healthy food consumption |
|  | General health | Physical health, physical condition, health-related quality of life, general health, frequency of flu (reverse coded), duration of illness (reverse coded) |
|  |  |  |
| Relationships | With teacher | Student-teacher relationship, rapport with teacher, trust in teacher, communication with teacher, conflictual relationship with teacher (reverse coded) |
|  | With parents | Mother-child relationship, experienced intimacy with parent, family conflict (revere coded), reciprocal filial piety |
|  | with peers | friendship - harmony, peer preference, classroom community, peer identification, number of friendships |
|  | Teaching quality | Satisfaction of teaching style, course evaluations, teacher effectiveness, instructor rating |
|  | academic satisfaction | School satisfaction, course satisfaction, happiness with math, school alienation (reverse coded) |
|  |  |  |
| Predictors of support provision | Teacher need satisfaction | Need satisfaction (SDT) |
|  | Parent need satisfaction | Need satisfaction (SDT) |
|  | Parent need frustration | Need frustration (SDT) |
|  | Parent ill-being | negative affect, stress, anxiety, depression, |
|  | Parent education level | Education level of mother, education level of father, parental education, highest parent education level |

Table S2. List of Countries from which Data were Sampled.

| Country | Count |  | Country | Count |
| --- | --- | --- | --- | --- |
| Angola | 1 |  | Japan | 3 |
| Argentina | 1 |  | Jordan | 1 |
| Australia | 12 |  | Lithuania | 4 |
| Austria | 8 |  | Malaysia | 3 |
| Barbados | 1 |  | Mauritius | 1 |
| Bedouin | 1 |  | Mexico | 1 |
| Belgium | 51 |  | Netherlands | 13 |
| Brazil | 1 |  | Norway | 8 |
| Canada | 46 |  | NZ | 1 |
| Chile | 1 |  | Pakistan | 1 |
| China | 60 |  | Peru | 2 |
| Colombia | 1 |  | Philippines | 3 |
| Croatia | 2 |  | Poland | 1 |
| Cyprus | 1 |  | Portugal | 5 |
| Dominican Republic | 3 |  | Russia | 2 |
| Estonia | 13 |  | Saudi Arabia | 1 |
| Finland | 10 |  | Singapore | 8 |
| France | 11 |  | South Korea | 18 |
| Georgia | 1 |  | Spain | 53 |
| Germany | 17 |  | Switzerland | 2 |
| Ghana | 2 |  | Taiwan | 6 |
| Greece | 15 |  | Tibet | 1 |
| Hungary | 3 |  | Turkey | 10 |
| Indonesia | 1 |  | Uganda | 1 |
| Iran | 7 |  | UK | 25 |
| Israel | 17 |  | US | 118 |
| Italy | 23 |  | Vietnam | 1 |
|  |  |  | Heterogeneous | 10 |

Table S3. Correlations between Support/Thwarting Behaviors and Basic Psychological Need Satisfaction/Thwarting

| Outcome | |  |  |  |  | 95% CI | | 80% Cred Int. | |  |  |  |
| --- | --- | --- | --- | --- | --- | --- | --- | --- | --- | --- | --- | --- |
|  | Supportive behaviors | k | n | *r* | ρ | Lower | Higher | Lower | Higher | t^2^ | t | I^2^ |
| Autonomy satisfaction | |  |  |  |  |  |  |  |  |  |  |  |
|  | Autonomy support | 124 | 106804 | .54 | .65 | .51 | .57 | .30 | .78 | .03 | .19 | 98.34 |
|  | Competence support | 30 | 18176 | .36 | .42 | .27 | .46 | .03 | .70 | .07 | .26 | 98.11 |
|  | Relatedness support | 26 | 16617 | .36 | .43 | .29 | .44 | .14 | .59 | .03 | .17 | 96.22 |
|  | Autonomy Thwarting | 10 | 5145 | -.25 | -.30 | -.37 | -.14 | -.47 | -.04 | .02 | .15 | 93.28 |
|  | Competence thwarting | 6 | 2776 | -.22 | -.28 | -.45 | .01 | -.54 | .09 | .05 | .21 | 95.87 |
|  | Relatedness thwarting | 8 | 4482 | -.27 | -.34 | -.41 | -.13 | -.50 | -.04 | .03 | .16 | 94.61 |
| Competence Satisfaction | |  |  |  |  |  |  |  |  |  |  |  |
|  | Autonomy support | 157 | 128577 | .40 | .48 | .37 | .42 | .19 | .60 | .03 | .16 | 96.70 |
|  | Competence support | 35 | 21128 | .35 | .41 | .26 | .44 | .01 | .69 | .07 | .26 | 98.18 |
|  | Relatedness support | 28 | 17466 | .33 | .38 | .27 | .39 | .12 | .53 | .02 | .15 | 94.94 |
|  | Autonomy Thwarting | 12 | 6236 | -.17 | -.20 | -.24 | -.09 | -.32 | -.02 | .01 | .11 | 86.98 |
|  | Competence thwarting | 6 | 2776 | -.20 | -.25 | -.36 | -.05 | -.41 | .01 | .02 | .14 | 90.93 |
|  | Relatedness thwarting | 8 | 4482 | -.24 | -.29 | -.36 | -.11 | -.43 | -.04 | .02 | .14 | 92.46 |
| Relatedness satisfaction | |  |  |  |  |  |  |  |  |  |  |  |
|  | Autonomy support | 123 | 621059 | .16 | .20 | .14 | .18 | .02 | .30 | .01 | .11 | 98.43 |
|  | Competence support | 32 | 531687 | .14 | .18 | .12 | .16 | .06 | .21 | .00 | .06 | 98.27 |
|  | Relatedness support | 30 | 537302 | .15 | .19 | .11 | .18 | .04 | .26 | .01 | .08 | 99.24 |
|  | Autonomy Thwarting | 11 | 5561 | -.14 | -.17 | -.25 | -.04 | -.34 | .05 | .02 | .14 | 91.59 |
|  | Competence thwarting | 6 | 2776 | -.24 | -.30 | -.43 | -.05 | -.49 | .01 | .03 | .17 | 93.87 |
|  | Relatedness thwarting | 9 | 5776 | -.24 | -.29 | -.38 | -.09 | -.50 | .02 | .03 | .18 | 96.06 |
| Autonomy Frustration | |  |  |  |  |  |  |  |  |  |  |  |
|  | Autonomy support | 26 | 10988 | -.26 | -.33 | -.32 | -.20 | -.45 | -0.07 | .02 | .14 | 90.65 |
|  | Competence support | 10 | 7188 | -.27 | -.31 | -.40 | -.15 | -.51 | -.04 | .03 | .17 | 95.95 |
|  | Relatedness support | 8 | 4795 | -.19 | -.21 | -.32 | -.07 | -.39 | .00 | .02 | .14 | 92.67 |
|  | Autonomy Thwarting | 12 | 7763 | .42 | .49 | .33 | .51 | .23 | .60 | .02 | .14 | 97.15 |
|  | Competence thwarting | 4 | 1891 | .27 | .35 | .19 | .36 | .22 | .33 | .00 | .03 | 36.25 |
|  | Relatedness thwarting | 7 | 3315 | .30 | .41 | .22 | .39 | .18 | .42 | .01 | .08 | 80.12 |
| Competence frustration | |  |  |  |  |  |  |  |  |  |  |  |
|  | Autonomy support | 17 | 8029 | -.16 | -.20 | -.20 | -.11 | -.26 | -.06 | .01 | .08 | 73.61 |
|  | Competence support | 10 | 7188 | -.34 | -.38 | -.45 | -.22 | -.56 | -.11 | .03 | .16 | 95.93 |
|  | Relatedness support | 8 | 4795 | -.20 | -.21 | -.28 | -.11 | -.33 | -.07 | .01 | .09 | 84.93 |
|  | Autonomy Thwarting | 10 | 6425 | .38 | .43 | .30 | .46 | .23 | .53 | .01 | .11 | 94.69 |
|  | Competence thwarting | 4 | 1891 | .35 | .44 | .26 | .43 | .29 | .40 | .00 | .03 | 39.34 |
|  | Relatedness thwarting | 7 | 3315 | .31 | .39 | .22 | .39 | .19 | .43 | .01 | .08 | 79.69 |
| Relatedness frustration | |  |  |  |  |  |  |  |  |  |  |  |
|  | Autonomy support | 19 | 8704 | -.18 | -.22 | -.23 | -.12 | -.32 | -.03 | .01 | .11 | 84.80 |
|  | Competence support | 9 | 5123 | -.23 | -.25 | -.36 | -.11 | -.45 | -.01 | .02 | .16 | 94.04 |
|  | Relatedness support | 9 | 6089 | -.29 | -.32 | -.41 | -.17 | -.50 | -.08 | .02 | .15 | 94.81 |
|  | Autonomy Thwarting | 9 | 5307 | .37 | .44 | .27 | .47 | .19 | .54 | .02 | .13 | 90.91 |
|  | Competence thwarting | 4 | 1891 | .34 | .43 | .11 | .57 | .12 | .56 | .02 | .14 | 91.83 |
|  | Relatedness thwarting | 8 | 4609 | .38 | .47 | .28 | .48 | .23 | .54 | .01 | .11 | 90.43 |

Note. Ρ is a reliability corrected estimate of the effect size.

Table S4. Correlations between Support/Thwarting Behaviors and Motivation Types as defined by Self-Determination Theory

| Outcome | |  |  |  |  | 95% CI | | 80% Cred Int. | |  |  |  |
| --- | --- | --- | --- | --- | --- | --- | --- | --- | --- | --- | --- | --- |
|  | Supportive behaviors | k | n | *r* | ρ | Lower | Higher | Lower | Higher | t^2^ | t | I^2^ |
| Intrinsic motivation | |  |  |  |  |  |  |  |  |  |  |  |
|  | Autonomy support | 109 | 109947 | .38 | .41 | .34 | .41 | .15 | .60 | .03 | .17 | 97.60 |
|  | Competence support | 16 | 13502 | .32 | .37 | .21 | .43 | .05 | .59 | .04 | .20 | 97.73 |
|  | Relatedness support | 15 | 13222 | .32 | .38 | .24 | .39 | .14 | .49 | .02 | .13 | 94.90 |
|  | Autonomy Thwarting | 8 | 14918 | .03 | .04 | -.07 | .13 | -.13 | .19 | .01 | .11 | 96.09 |
| Identified regulation | |  |  |  |  |  |  |  |  |  |  |  |
|  | Autonomy support | 65 | 73961 | .41 | .45 | .37 | .45 | .22 | .59 | .02 | .14 | 97.10 |
|  | Competence support | 14 | 9880 | .29 | .34 | .19 | .39 | .06 | .51 | .03 | .17 | 95.92 |
|  | Relatedness support | 11 | 10127 | .30 | .37 | .22 | .39 | .13 | .48 | .02 | .13 | 94.85 |
|  | Autonomy Thwarting | 6 | 14248 | .07 | .08 | -.01 | .16 | -.05 | .19 | .01 | .08 | 93.75 |
| Introjected regulation | |  |  |  |  |  |  |  |  |  |  |  |
|  | Autonomy support | 55 | 66646 | .08 | .10 | .06 | .11 | -.03 | .20 | .01 | .09 | 90.44 |
|  | Competence support | 9 | 5108 | .14 | .18 | .05 | .23 | -.01 | .29 | .01 | .11 | 87.12 |
|  | Relatedness support | 10 | 5732 | .15 | .20 | .07 | .23 | .00 | .30 | .01 | .11 | 87.72 |
|  | Autonomy Thwarting | 6 | 14248 | .12 | .15 | .07 | .18 | .06 | .19 | .00 | .05 | 84.43 |
| External regulation | |  |  |  |  |  |  |  |  |  |  |  |
|  | Autonomy support | 70 | 73375 | -.04 | -.05 | -.08 | -.01 | -.24 | .15 | .02 | .15 | 95.81 |
|  | Competence support | 14 | 6335 | .04 | .05 | -.07 | .15 | -.20 | .28 | .03 | .18 | 93.64 |
|  | Relatedness support | 12 | 6197 | .00 | .00 | -.13 | .14 | -.27 | .28 | .04 | .20 | 95.52 |
|  | Autonomy Thwarting | 7 | 14503 | .18 | .22 | .11 | .25 | .07 | .29 | .01 | .07 | 92.42 |
| Amotivation | |  |  |  |  |  |  |  |  |  |  |  |
|  | Autonomy support | 49 | 53245 | -.28 | -.32 | -.32 | -.24 | -.45 | -.10 | .02 | .13 | 95.79 |
|  | Competence support | 15 | 8507 | -.15 | -.18 | -.22 | -.07 | -.32 | .02 | .02 | .13 | 90.58 |
|  | Relatedness support | 14 | 8366 | -.18 | -.22 | -.26 | -.10 | -.36 | .00 | .02 | .13 | 91.57 |
|  | Autonomy Thwarting | 12 | 6417 | .37 | .40 | .28 | .46 | .18 | .56 | .02 | .14 | 93.13 |
| Autonomous motivation | |  |  |  |  |  |  |  |  |  |  |  |
|  | Autonomy support | 148 | 77671 | .39 | .44 | .36 | .41 | .20 | .58 | .02 | .15 | 94.20 |
|  | Competence support | 23 | 17057 | .32 | .38 | .25 | .40 | .09 | .55 | .03 | .17 | 96.56 |
|  | Relatedness support | 16 | 13019 | .31 | .37 | .22 | .41 | .09 | .54 | .03 | .17 | 96.63 |
|  | Autonomy Thwarting | 12 | 5192 | -.10 | -.10 | -.22 | .02 | -.35 | .15 | .03 | .18 | 93.76 |
| Controlled motivation | |  |  |  |  |  |  |  |  |  |  |  |
|  | Autonomy support | 106 | 83124 | .01 | .01 | -.02 | .04 | -.18 | .20 | .02 | .14 | 94.25 |
|  | Competence support | 23 | 11998 | .07 | .08 | -.01 | .15 | -.17 | .30 | .03 | .18 | 94.39 |
|  | Relatedness support | 17 | 10024 | .08 | .10 | -.04 | .20 | -.23 | .40 | .05 | .23 | 97.02 |
|  | Autonomy Thwarting | 11 | 6038 | .30 | .34 | .22 | .38 | .14 | .46 | .01 | .12 | 90.30 |
| RAI |  |  |  |  |  |  |  |  |  |  |  |  |
|  | Autonomy support | 83 | 86271 | .39 | .45 | .37 | .42 | .23 | .55 | .02 | .12 | 95.66 |
|  | Competence support | 5 | 1530 | .25 | .28 | -.02 | .53 | -.08 | .58 | .05 | .21 | 94.10 |
|  | Relatedness support | 5 | 1437 | .33 | .37 | .18 | .48 | .17 | .50 | .01 | .11 | 80.46 |
|  | Autonomy Thwarting | 10 | 19393 | -.07 | -.08 | -.32 | .19 | -.56 | .42 | .13 | .35 | 99.59 |

Note. ρ is a reliability corrected estimate of the effect size. Autonomous motivation is a composite variable typically calculated by combining intrinsic motivation and identified regulation. Controlled motivation is a composite created typically by combining external and introjected regulations.

Table S5. Calculated Average Alpha Coefficient for Variables with Missing Reliability Information

| variable | average alpha | variable | average alpha | variable | average alpha |
| --- | --- | --- | --- | --- | --- |
| Autonomy support | 0.84 | Negative coping | 0.82 | Well-being_satisfaction_life | 0.82 |
| Competence support | 0.74 | Misconduct (all) | 0.82 | Well-being satisfaction_academic | 0.85 |
| Relatedness support | 0.72 | Prosocial behavior (parent) | 0.80 | Well-being emotional | 0.87 |
| Autonomy thwarting | 0.80 | Interpersonal skills | 0.88 | Well-being | 0.86 |
| Autonomy satisfaction | 0.77 | Cognitive skills | 0.75 | Self-esteem | 0.82 |
| Competence satisfaction | 0.79 | Academic performance | 0.92 | Physical health | 0.87 |
| Relatedness satisfaction | 0.83 | Performance | 0.90 | Negative affect | 0.83 |
| Autonomy frustration | 0.77 | Learning strategies | 0.88 | Ill-being | 0.82 |
| Competence frustration | 0.82 | Identity development | 0.79 | Depression | 0.84 |
| Relatedness frustration | 0.81 | Creativity | 0.83 | Anxiety | 0.85 |
| Global satisfaction | 0.85 | Self-regulation_emotional | 0.81 | Engagement | 0.85 |
| Global frustration | 0.82 | Self-regulation | 0.79 | Engagement_behavior | 0.79 |
| Amotivation | 0.79 | Prosocial behavior | 0.77 | Persistence | 0.80 |
| External regulation | 0.75 | Positive coping | 0.75 | Engagement_state | 0.84 |
| Introjection | 0.73 | Healthy lifestyle _PA/eating | 0.83 | Engagement_emotional | 0.84 |
| Identified | 0.80 | Psychical activity intention | 0.90 | Disengagement | 0.80 |
| Integrated | 0.84 | Intention | 0.89 | Self-efficacy | 0.83 |
| Intrinsic | 0.84 | Relationship quality | 0.83 | Valuation | 0.80 |
| Autonomous | 0.86 | Teacher motivation | 0.79 | Perceived control | 0.78 |
| Controlled | 0.80 | Teacher quality | 0.86 | Norm | 0.76 |
| RAI | 0.84 | Traits | 0.79 | Goal orientation mastery | 0.80 |
|  |  | Causality orientations | 0.76 | Attitude | 0.88 |

Table S6. Egger's Test Results for Autonomy Support

|  | Intercept | CI | *p* | Egger's z |
| --- | --- | --- | --- | --- |
| Basic psychological needs |  |  |  |  |
| Global frustration | -0.30 | (-0.4401,-0.1517) | 0.43 | 0.79 |
| Global satisfaction | 0.62 | (0.5224,0.7143) | 0.01 | -2.46* |
| Relatedness satisfaction | 0.44 | (0.3513,0.5275) | 0.39 | -0.87 |
| Competence satisfaction | 0.42 | (0.3601,0.4726) | < .01 | -2.95* |
| Autonomy satisfaction | 0.68 | (0.5863,0.7795) | < .01 | -3.50* |
| Autonomy frustration | -0.20 | (-0.3824,-0.0142) | 0.72 | -0.36 |
| Relatedness frustration | -0.20 | (-0.4000,-0.0062) | 0.60 | 0.53 |
| Competence frustration | -0.17 | (-0.2873,-0.0543) | 0.67 | 0.42 |
| Demographics and traits |  |  |  |  |
| Autonomous causality orientation | 0.29 | ( 0.0147, 0.5739) | 0.92 | -0.10 |
| Controlled causality orientation | -0.17 | ( -0.5311, 0.2005) | 0.59 | 0.54 |
| Agreeableness | 0.34 | ( 0.1121, 0.5624) | 0.22 | -1.22 |
| Conscientiousness | 0.35 | ( 0.2495, 0.4493) | 0.02 | -2.33* |
| Extraversion | 0.19 | ( 0.0853, 0.2873) | 0.75 | -0.31 |
| Neuroticism | -0.28 | ( -0.5057, -0.0581) | 0.13 | 1.51 |
| Openness | 0.23 | ( -0.0682, 0.5380) | 0.27 | -1.10 |
| Age | -0.06 | (-0.1366,0.0091) | 0.32 | 0.99 |
| SES | -0.04 | (-0.0992,0.0138) | < .01 | 4.30* |
| Parents and teacher |  |  |  |  |
| Parent need satisfaction | 0.26 | ( -0.2430, 0.7602) | 0.52 | 0.64 |
| Parent education | 0.14 | ( 0.0214, 0.2503) | 0.52 | -0.64 |
| Parent ill-being | -0.18 | ( -0.3576, -0.0002) | 1.00 | 0.00 |
| Parent need frustration | 0.09 | ( -0.2814, 0.4556) | 0.13 | -1.53 |
| Relationship quality |  |  |  |  |
| Relationship with parents | 0.39 | ( 0.2643, 0.5136) | 0.12 | -1.55 |
| Relationship with peers | 0.34 | ( 0.0670, 0.6172) | 0.46 | -0.74 |
| Relationship with teacher | 0.56 | ( 0.3120, 0.8039) | 0.05 | -1.92 |
| Teaching quality | 0.16 | ( -0.1328, 0.4487) | < .01 | 4.01* |
| Academic satisfaction | 0.68 | ( 0.2751, 1.0915) | 0.30 | -1.03 |
| Performance |  |  |  |  |
| Performance (general) | 0.18 | ( 0.1367, 0.2309) | 0.36 | -0.91 |
| Grade point average | 0.00 | ( -0.0722, 0.0717) | < .01 | 3.39* |
| Cognitive skills | 0.19 | ( 0.0563, 0.3312) | 0.52 | 0.64 |
| Learning strategy |  |  |  |  |
| Learning strategy use | 0.46 | ( 0.3179, 0.6048) | 0.11 | -1.61 |
| Deep learning | 0.47 | ( 0.1366, 0.8061) | 0.39 | -0.86 |
| Metacognition | 0.41 | ( 0.2419, 0.5708) | 0.81 | -0.24 |
| Time management | 0.37 | ( 0.1845, 0.5590) | 0.98 | -0.03 |
| Goal setting / planning | 0.23 | ( 0.0122, 0.4538) | 0.23 | 1.19 |
| Motivational beliefs |  |  |  |  |
| Self-regulation | 0.10 | ( -0.0329, 0.2257) | 0.16 | 1.39 |
| Perceived control | 0.21 | ( 0.0722, 0.3449) | 0.16 | 1.42 |
| Self-efficacy | 0.41 | ( 0.2479, 0.5624) | 0.58 | -0.56 |
| Valuation | 0.41 | ( 0.2035, 0.6112) | 0.90 | 0.13 |
| Attitude | 0.35 | ( 0.2253, 0.4689) | 0.74 | 0.33 |
| Performance goal orientation | 0.06 | ( -0.1213, 0.2466) | 0.94 | 0.08 |
| Performance approach | 0.26 | ( -0.0827, 0.6110) | 0.32 | -1.00 |
| Performance avoidance | -0.05 | ( -0.4888, 0.3867) | 0.97 | 0.03 |
| Mastery goal orientation | 0.21 | ( 0.0514, 0.3594) | 0.16 | 1.40 |
| Mastery approach | 0.13 | ( -0.1035, 0.3663) | 0.02 | 2.29* |
| Mastery avoidance | -0.24 | ( -0.9324, 0.4590) | 0.43 | 0.79 |
| Motivation |  |  |  |  |
| Autonomous motivation | 0.42 | ( 0.3625, 0.4694) | 0.13 | -1.53 |
| Controlled motivation | 0.05 | ( -0.0187, 0.1119) | 0.33 | -0.97 |
| External regulation | -0.03 | ( -0.1153, 0.0482) | 0.59 | -0.54 |
| Intrinsic motivation | 0.42 | ( 0.3568, 0.4900) | 0.27 | -1.11 |
| Identified regulation | 0.41 | ( 0.3351, 0.4763) | 0.55 | -0.60 |
| Integrated regulation | 0.30 | ( 0.0963, 0.4999) | 0.63 | 0.49 |
| Introjected regulation | 0.14 | ( 0.0791, 0.2090) | 0.29 | -1.06 |
| Amotivation | -0.21 | ( -0.2773, -0.1374) | 0.87 | 0.16 |
| RAI | 0.44 | ( 0.3620, 0.5169) | < .01 | -3.69* |
| Engagement |  |  |  |  |
| Disengagement (behavior) | -0.25 | (-0.4265,-0.0716) | 1.00 | 0.00 |
| Disengagement (emotional) | -0.45 | (-0.6782,-0.2258) | 0.10 | 1.64 |
| Engagement (general) | 0.42 | (0.3027,0.5313) | 0.64 | 0.47 |
| Engagement (behavior) | 0.35 | ( 0.2565, 0.4420) | 0.50 | -0.67 |
| Engagement (emotional) | 0.44 | ( 0.3011, 0.5706) | 0.99 | 0.01 |
| Cognitive engagement | 0.51 | (0.3173,0.7059) | 0.20 | -1.29 |
| State engagement | 0.61 | ( 0.4868, 0.7310) | < .01 | -3.78* |
| Intention to act | 0.29 | ( 0.1467, 0.4265) | 0.26 | 1.12 |
| Specific engagement |  |  |  |  |
| Engagement proactive | 0.39 | ( 0.1149, 0.6563) | 0.55 | 0.60 |
| Engagement (time) | 0.01 | ( -0.1258, 0.1526) | 0.71 | 0.38 |
| Persistence | 0.16 | ( 0.0146, 0.3075) | 0.32 | 1.00 |
| Prosocial behavior | 0.15 | ( 0.0827, 0.2241) | 0.14 | 1.48 |
| Negative behavior |  |  |  |  |
| Externalized misconduct | -0.15 | ( -0.2137, -0.0959) | 0.64 | -0.47 |
| Procrastination | 0.13 | ( -0.0933, 0.3580) | 0.01 | -2.61* |
| Absenteeism | -0.23 | ( -0.3213, -0.1355) | 0.04 | 2.10* |
| Drop out | -0.11 | ( -0.2442, 0.0210) | 0.01 | -2.63* |
| Negative coping strategies | -0.17 | ( -0.2800, -0.0621) | 0.47 | -0.72 |
| Positive behaviour |  |  |  |  |
| Emotional regulation | 0.13 | ( -0.0941, 0.3510) | 0.98 | 0.02 |
| Positive coping strategies | 0.31 | ( 0.1701, 0.4445) | 0.03 | -2.20* |
| Interpersonal skills | 0.50 | ( 0.3063, 0.6911) | 0.39 | -0.86 |
| Well-being |  |  |  |  |
| Well-being (general) | 0.39 | ( 0.2896, 0.4954) | 0.37 | -0.90 |
| Emotional well-being (PA) | 0.01 | ( 0.0392, 0.3409) | 0.19 | 2.73 |
| Eudaimonia well-being | 0.34 | ( 0.2138, 0.4656) | 0.37 | 0.89 |
| Self-esteem | 0.20 | ( 0.1299, 0.2693) | 0.08 | 1.77 |
| General ill-being | -0.08 | ( -0.1661, 0.0092) | 0.04 | -2.04* |
| Emotional ill-being (NA) | -0.22 | ( -0.3252, -0.1244) | 0.69 | 0.40 |
| Anxiety | -0.12 | ( -0.2374, -0.0087) | 0.03 | -2.21* |
| Depression | -0.23 | ( -0.3127, -0.1443) | 0.79 | -0.27 |
| Health |  |  |  |  |
| Physical activity intention | 0.27 | ( 0.0569, 0.4789) | 0.50 | 0.68 |
| Healthy lifestyle | 0.25 | ( 0.1552, 0.3440) | 0.21 | -1.25 |
| General health | 0.12 | ( 0.0625, 0.1741) | 0.05 | 1.96 |

Table S7. Egger's Test Results for Competence Support

|  | Intercept | CI | *p* | Egger's z |
| --- | --- | --- | --- | --- |
| Basic psychological needs |  |  |  |  |
| Global satisfaction | 1.01 | ( 0.2993, 1.7182) | 0.44 | -0.77 |
| Autonomy frustration | -0.39 | ( -0.6267, -0.1473) | 0.16 | 1.40 |
| Autonomy satisfaction | 0.51 | ( 0.3346, 0.6810) | 0.25 | -1.15 |
| Competence frustration | -0.49 | ( -0.6654, -0.3143) | 0.01 | 2.54* |
| Competence satisfaction | 0.48 | ( 0.3531, 0.6165) | 0.10 | -1.66 |
| Relatedness frustration | -0.15 | ( -0.4077, 0.1167) | 0.53 | -0.62 |
| Relatedness satisfaction | 0.34 | ( 0.1913, 0.4800) | 0.51 | 0.66 |
| Demographics and traits |  |  |  |  |
| Autonomous causality orientation | 0.39 | ( 0.1812, 0.6059) | 0.76 | -0.30 |
| Performance |  |  |  |  |
| Performance (general) | -0.03 | ( -0.1656, 0.1030) | 0.03 | 2.21* |
| Grade point average | -0.07 | ( -0.2238, 0.0888) | 0.05 | 1.98* |
| Motivational beliefs |  |  |  |  |
| Self-efficacy | 0.63 | ( 0.2870, 0.9773) | 0.04 | -2.05* |
| Motivation |  |  |  |  |
| Amotivation | -0.09 | ( -0.2182, 0.0412) | 0.05 | -1.93 |
| Autonomous motivation | 0.39 | ( 0.1982, 0.5830) | 0.58 | -0.56 |
| Controlled motivation | 0.18 | ( 0.0073, 0.3537) | 0.08 | -1.78 |
| External motivation | 0.03 | ( -0.1797, 0.2478) | 0.69 | -0.40 |
| Identified motivation | 0.33 | ( 0.1187, 0.5403) | 0.76 | 0.30 |
| Intrinsic motivation | 0.34 | ( 0.1299, 0.5420) | 0.88 | 0.15 |
| Introjected motivation | 0.22 | ( 0.0542, 0.3836) | 0.25 | -1.15 |
| Engagement |  |  |  |  |
| Engagement (general) | 0.42 | ( -0.0182, 0.8498) | 0.60 | 0.52 |
| Engagement (behavior) | 0.20 | ( -0.0012, 0.4023) | 0.53 | 0.63 |
| Engagement (emotional) | 0.27 | ( 0.0339, 0.5088) | 0.19 | 1.31 |
| Cognitive engagement | 0.55 | ( 0.0132, 1.0859) | 0.41 | -0.83 |
| Negative behavior |  |  |  |  |
| Externalized misconduct | -0.25 | ( -0.3366, -0.1611) | 0.26 | 1.12 |
| Well-being |  |  |  |  |
| Emotional ill-being (NA) | -0.07 | ( -0.1787, 0.0472) | 0.03 | -2.14* |

Table S8. Egger's Test Results for Relatedness Support

|  | Intercept | CI | *p* | Egger's z |
| --- | --- | --- | --- | --- |
| Basic psychological needs |  |  |  |  |
| Autonomy frustration | -0.24 | ( -0.4396, -0.0472) | 0.43 | 0.80 |
| Autonomy satisfaction | 0.41 | ( 0.1310, 0.6941) | 0.95 | -0.07 |
| Competence frustration | -0.35 | ( -0.4250, -0.2821) | < .01 | 4.68* |
| Competence satisfaction | 0.38 | ( 0.1937, 0.5716) | 0.76 | -0.30 |
| Relatedness frustration | -0.45 | ( -0.7468, -0.1623) | 0.16 | 1.40 |
| Relatedness satisfaction | 0.53 | ( 0.2769, 0.7747) | 0.76 | -0.30 |
| Demographics |  |  |  |  |
| Age | -0.06 | ( -0.3105, 0.1963) | 0.50 | 0.67 |
| SES | 0.03 | ( -0.0801, 0.1337) | 0.16 | 1.41 |
| Performance |  |  |  |  |
| Performance (general) | 0.16 | ( -0.0223, 0.3516) | 0.32 | -0.99 |
| Grade point average | -0.11 | ( -0.2198, -0.0034) | 0.01 | 2.52* |
| Motivational beliefs |  |  |  |  |
| Self-efficacy | 0.82 | ( 0.5473, 1.0939) | < .01 | -4.25* |
| Self-regulation | 0.36 | ( 0.1683, 0.5552) | < .01 | -3.32* |
| Motivation |  |  |  |  |
| Amotivation | -0.15 | ( -0.3134, 0.0053) | 0.55 | -0.60 |
| Autonomous motivation | 0.35 | ( 0.1278, 0.5685) | 0.86 | -0.18 |
| Controlled motivation | 0.19 | ( -0.0509, 0.4377) | 0.21 | -1.25 |
| External regulation | 0.03 | ( -0.1982, 0.2648) | 0.87 | -0.17 |
| Identified regulation | 0.34 | ( 0.1583, 0.5179) | 0.57 | 0.57 |
| Intrinsic motivation | 0.33 | ( 0.1520, 0.5178) | 0.60 | 0.53 |
| Introjected regulation | 0.27 | ( 0.1089, 0.4329) | 0.03 | -2.15* |
| Engagement |  |  |  |  |
| Engagement (general) | 0.23 | ( -0.4157, 0.8721) | 0.45 | 0.75 |
| Engagement (behavior) | 0.10 | ( -0.2414, 0.4511) | 0.39 | 0.87 |
| Engagement (emotional) | 0.21 | ( -0.0099, 0.4269) | 0.07 | 1.82 |
| Persistence | 0.28 | ( 0.0674, 0.4937) | 0.50 | -0.67 |
| Negative behaviour |  |  |  |  |
| Externalized misconduct | -0.18 | ( -0.3208, -0.0485) | 0.68 | -0.42 |
| Well-being |  |  |  |  |
| Well-being (general) | 0.19 | ( 0.0041, 0.3768) | 0.18 | 1.35 |
| Emotional well-being (PA) | 0.13 | ( -0.2307, 0.4809) | 0.04 | 2.09* |
| Eudaimonia well-being | 0.26 | ( 0.0337, 0.4899) | 0.35 | 0.93 |
| Self-esteem | 0.08 | ( -0.0991, 0.2654) | 0.18 | 1.35 |
| General ill-being | -0.03 | ( -0.1326, 0.0718) | < .01 | -3.07* |
| Emotional ill-being (NA) | -0.07 | ( -0.3151, 0.1707) | 0.08 | -1.75 |
| Anxiety | -0.18 | ( -0.3723, 0.0107) | 0.06 | -1.90 |
| Depression | -0.26 | ( -0.4354, -0.0917) | 0.92 | -0.10 |

Table S9. Egger's Test Results for Thwarting Behavior

|  | Intercept | CI | *p* | Egger's z |
| --- | --- | --- | --- | --- |
| **Autonomy thwarting** |  |  |  |  |
| Basic psychological needs |  |  |  |  |
| Global frustration | 0.62 | ( 0.4353, 0.8009) | 0.58 | -0.56 |
| Global satisfaction | -0.44 | ( -0.6692, -0.2186) | 0.20 | 1.28 |
| Autonomy frustration | 0.51 | ( 0.3136, 0.7148) | 0.18 | -1.35 |
| Competence frustration | 0.49 | ( 0.3456, 0.6386) | 0.03 | -2.22* |
| Relatedness frustration | 0.31 | ( 0.0760, 0.5377) | 0.74 | 0.33 |
| Autonomy satisfaction | 0.29 | ( -0.1317, 0.7037) | 0.01 | -2.63* |
| Competence satisfaction | 0.04 | ( -0.3111, 0.3830) | 0.24 | -1.18 |
| Relatedness satisfaction | 0.11 | ( -0.2980, 0.5223) | 0.22 | -1.23 |
| Performance |  |  |  |  |
| Performance (general) | -0.15 | ( -0.2522, -0.0446) | 0.81 | -0.24 |
| Grade point average | -0.28 | ( -0.7056, 0.1478) | 0.37 | 0.90 |
| Motivation |  |  |  |  |
| Amotivation | 0.36 | ( 0.2073, 0.5085) | 0.79 | -0.27 |
| Autonomous motivation | -0.08 | ( -0.2437, 0.0814) | 0.34 | -0.95 |
| Controlled motivation | 0.25 | ( 0.0806, 0.4204) | 0.85 | -0.18 |
| External motivation | 0.24 | ( 0.1015, 0.3721) | 0.74 | 0.33 |
| Identified regulation | -0.04 | ( -0.1801, 0.1002) | 0.81 | 0.24 |
| Index | -0.25 | ( -0.8223, 0.3193) | 0.45 | -0.76 |
| Intrinsic motivation | -0.07 | ( -0.2593, 0.1115) | 0.34 | -0.96 |
| Introjected regulation | 0.17 | ( 0.0376, 0.3004) | 0.85 | -0.19 |
| Engagement |  |  |  |  |
| Engagement (behavior) | -0.04 | ( -0.1936, 0.1137) | 0.68 | -0.42 |
| Negative behavior |  |  |  |  |
| Externalized misconduct | 0.35 | ( 0.0374, 0.6707) | 0.66 | -0.43 |
| Positive behavior |  |  |  |  |
| Healthy lifestyle (PA) | -0.29 | ( -0.5904, 0.0063) | 0.06 | 1.88 |
| Self-regulation | -0.42 | ( -1.0120, 0.1707) | 0.40 | 0.85 |
| Well-being |  |  |  |  |
| Emotional ill-being (NA) | 0.52 | ( 0.3688, 0.6620) | 0.02 | -2.42* |
| Emotional well-being (PA) | -0.51 | ( -0.7706, -0.2455) | 0.08 | 1.72 |
| **Relationship thwarting** |  |  |  |  |
| Autonomy frustration | 0.29 | ( -0.0065, 0.5912) | 0.92 | 0.10 |
| Competence frustration | 0.33 | ( 0.0527, 0.6139) | 0.84 | -0.20 |
| Relatedness frustration | 0.43 | ( 0.1176, 0.7421) | 0.84 | -0.20 |
| Autonomy satisfaction | -0.11 | ( -1.0061, 0.7824) | 0.70 | -0.38 |
| Competence satisfaction | -0.22 | ( -0.9749, 0.5384) | 0.96 | -0.06 |
| Relatedness satisfaction | 0.14 | ( -0.6701, 0.9540) | 0.28 | -1.09 |
| Emotional well-being (PA) | 0.11 | ( -0.0401, 0.2669) | < .01 | -4.03* |
| **Competence thwarting** |  |  |  |  |
| Autonomy satisfaction | 0.33 | ( -1.5256, 2.1946) | 0.54 | -0.62 |
| Competence satisfaction | 0.23 | ( -0.9808, 1.4365) | 0.47 | -0.73 |
| Relatedness satisfaction | -0.19 | ( -1.7867, 1.4111) | 0.94 | -0.08 |

**List of Studies Included in the Meta-Analysis**

Abos, A., Sevil, J., Sanz, M., Aibar, A., & Garcia-Gonzalez, L. (2016). Autonomy support in Physical Education as a means of preventing students' oppositional defiance. RICYDE-REVISTA INTERNACIONAL DE CIENCIAS DEL DEPORTE, 12(43), 65-78.

Abós, Á., Burgueño, R., García-González, L, & Sevil-Serrano, J. (2021). Influence of internally and externally controlling teaching behaviors on students’ motivational outcomes in Physical Education: Is there a gender difference?. Journal of Teaching in Physical Education. https://doi.org/10.1123/jtpe.2020-0316

Abula, K., Beckmann, J., He, Z., Cheong, C., Lu, F., & Gröpel, P. (2020). Autonomy support in physical education promotes autonomous motivation towards leisure-time physical activity: evidence from a sample of Chinese college students. Health promotion international, 35(1), e1-e10.

Adams, C., & Khojasteh, J. (2018). Igniting students’ inner determination: The role of a need-supportive climate. Journal of Educational Administration.

Adams, C. M., Olsen, J. J., & Ware, J. K. (2017). The school principal and student learning capacity. Educational Administration Quarterly, 53(4), 556-584.

Adie, J., & Wakefield, C. (2011). Perceptions of the teaching environment, engagement and burnout among university students on a sports-related degree programme in the UK. Journal of Hospitality, Leisure, Sports and Tourism Education (Pre-2012), 10(2), 74.

Aelterman, N., Vansteenkiste, M., Van den Berghe, L., De Meyer, J., & Haerens, L. (2014). Fostering a need-supportive teaching style: Intervention effects on physical education teachers’ beliefs and teaching behaviors. Journal of Sport and Exercise Psychology, 36(6), 595-609.

Aelterman, N., Vansteenkiste, M., Haerens, L., Soenens, B., Fontaine, J. R., & Reeve, J. (2019). Toward an integrative and fine-grained insight in motivating and demotivating teaching styles: The merits of a circumplex approach. Journal of Educational Psychology, 111(3), 497.

Aibar, A., Abós, Á., García-González, L., González-Cutre, D., & Sevil-Serrano, J. (2021). Understanding students’ novelty satisfaction in physical education: Associations with need-supportive teaching style and physical activity intention. European Physical Education Review, 1356336X21992791.

Akın, R. I., Breeman, L. D., Meeus, W., & Branje, S. (2020). Parent-adolescent relationship quality as a predictor of leaving home. Journal of adolescence, 79, 81-90.

Al‐Dhamit, Y., & Kreishan, L. (2016). Gifted students' intrinsic and extrinsic motivations and parental influence on their motivation: from the self‐determination theory perspective. Journal of Research in Special Educational Needs, 16(1), 13-23.

Alrabai, F. (2021). The Influence of Autonomy-Supportive Teaching on EFL Students' Classroom Autonomy: An Experimental Intervention. Frontiers in psychology, 3748.

Álvarez, O., Castillo, I., & Moreno-Pellicer, R. (2019). Transformational Parenting Style, Autonomy Support, and their Implications for Adolescent Athletes’ Burnout. Psychosocial Intervention, 28(2), 91-100.

Amorose, A. J., Anderson-Butcher, D., Newman, T. J., Fraina, M., & Iachini, A. (2016). High school athletes’ self-determined motivation: The independent and interactive effects of coach, father, and mother autonomy support. Psychology of Sport and Exercise, 26, 1-8.

Amoura, C., Berjot, S., Gillet, N., Caruana, S., & Finez, L. (2015). Effects of autonomy-supportive and controlling styles on situational self-determined motivation: Some unexpected results of the commitment procedure. Psychological reports, 116(1), 33-59.

Amoura, C., Berjot, S., Gillet, N., Caruana, S., Cohen, J., & Finez, L. (2015). Autonomy-supportive and controlling styles of teaching. Swiss Journal of Psychology.

Audet, É. C., Levine, S. L., Holding, A. C., Koestner, R., & Powers, T. A. (2021). A remarkable alliance: Sibling autonomy support and goal progress in emerging adulthood. Family Relations.

Aunola, K., Viljaranta, J., Lehtinen, E., & Nurmi, J. E. (2013). The role of maternal support of competence, autonomy and relatedness in children's interests and mastery orientation. Learning and Individual Differences, 25, 171-177.

Austin, S., Guay, F., Senécal, C., Fernet, C., & Nouwen, A. (2013). Longitudinal testing of a dietary self-care motivational model in adolescents with diabetes. Journal of Psychosomatic Research, 75(2), 153-159.

Bagøien, T. E., Halvari, H., & Nesheim, H. (2010). Self-determined motivation in physical education and its links to motivation for leisure-time physical activity, physical activity, and well-being in general. Perceptual and Motor Skills, 111(2), 407-432.

Baker, J. P., & Goodboy, A. K. (2019). The choice is yours: the effects of autonomy-supportive instruction on students’ learning and communication. Communication Education, 68(1), 80-102.

Barberis, N., Gugliandolo, M. C., Costa, S., & Liga, F. (2021). How parental autonomy support prevent from adolescents’ depression and low self-esteem: a mediational model with trait Emotional Intelligence. Mediterranean Journal of Clinical Psychology, 9(1).

Barkoukis, V., Chatzisarantis, N., & Hagger, M. S. (2020). Effects of a School-Based Intervention on Motivation for Out-of-School Physical Activity Participation. Research Quarterly for Exercise and Sport, 1-15.

Barni, D., Russo, C., Zagrean, I., Di Fabio, M., & Danioni, F. (2020). Adolescents’ internalization of moral values: the role of paternal and maternal promotion of volitional functioning. Journal of Family Studies, 1-13.

Bartholomew, K. J., Ntoumanis, N., Mouratidis, A., Katartzi, E., Thøgersen-Ntoumani, C., & Vlachopoulos, S. (2018). Beware of your teaching style: A school-year long investigation of controlling teaching and student motivational experiences. Learning and Instruction, 53, 50-63.

Behzadnia, B., Adachi, P. J., Deci, E. L., & Mohammadzadeh, H. (2018). Associations between students' perceptions of physical education teachers' interpersonal styles and students' wellness, knowledge, performance, and intentions to persist at physical activity: A self-determination theory approach. Psychology of Sport and Exercise, 39, 10-19.

Behzadnia, B. (2020). The relations between students’ causality orientations and teachers’ interpersonal behaviors with students’ basic need satisfaction and frustration, intention to physical activity, and well-being. Physical Education and Sport Pedagogy, 1-20.

Behzadnia, B., Rezaei, F., & Salehi, M. (2022). A need-supportive teaching approach among students with intellectual disability in physical education. Psychology of Sport and Exercise, 60, 102156.

Behzadnia, B., Alizadeh, E., Haerens, L., & Aghdasi, M. T. (2022). Changes in students’ goal pursuits and motivational regulations toward healthy behaviors during the pandemic: A Self-Determination Theory perspective. Psychology of Sport and Exercise, 59, 102131.

Behzadnia, B. (2021). The relations between students’ causality orientations and teachers’ interpersonal behaviors with students’ basic need satisfaction and frustration, intention to physical activity, and well-being. Physical Education and Sport Pedagogy, 26(6), 613-632.

Beiswenger, K. L., & Grolnick, W. S. (2010). Interpersonal and intrapersonal factors associated with autonomous motivation in adolescents’ after-school activities. The Journal of Early Adolescence, 30(3), 369-394.

Benatov, J., Brunstein-Klomek, A., & Chen-Gal, S. (2021). Suicide Behavior Among Vocational High School Students: The Role of School-Related Factors. School Mental Health, 1-12.

Benita, M., & Matos, L. (2021). Internalization of mastery goals: The differential effect of teachers’ autonomy support and control. Frontiers in Psychology, 11, 599303.

Benlahcene, A., Awang-Hashim, R., Kaur, A., & Wan-Din, W. Z. (2021). Perceived autonomy support and agentic engagement among Malaysian undergraduates: the mediatory role of personal best goals. Journal of Further and Higher Education, 1-13.

Benlahcene, A., Awang-Hashim, R., & Kaur, A. (2020). Personal Best Goals: Do They Mediate the Relationship between Teacher Autonomy Support and Student Engagement?. Malaysian Journal of Learning and Instruction, 17(1), 25-49.

Bennett, M., Ng-Knight, T., & Hayes, B. (2017). Autonomy-supportive teaching and its antecedents: differences between teachers and teaching assistants and the predictive role of perceived competence. European Journal of Psychology of Education, 32(4), 643-667.

Berger, J. L., & Girardet, C. (2021). Vocational teachers’ classroom management style: The role of motivation to teach and sense of responsibility. European Journal of Teacher Education, 44(2), 200-216.

Beyens, I., & Valkenburg, P. M. (2019). Parental Media Mediation in Adolescence: A Comparative Study of Parent and Adolescent Reports. Journal of Broadcasting & Electronic Media, 63(4), 716-736.

Bindman, S. W., Pomerantz, E. M., & Roisman, G. I. (2015). Do children’s executive functions account for associations between early autonomy-supportive parenting and achievement through high school?. Journal of Educational Psychology, 107(3), 756.

Black, A. E., & Deci, E. L. (2000). The effects of instructors' autonomy support and students' autonomous motivation on learning organic chemistry: A self‐determination theory perspective. Science education, 84(6), 740-756.

Boggiano, A. K. (1998). Maladaptive achievement patterns: A test of a diathesis–stress analysis of helplessness. Journal of Personality and Social Psychology, 74(6), 1681.

Bonem, E. M., Fedesco, H. N., & Zissimopoulos, A. N. (2019). What you do is less important than how you do it: the effects of learning environment on student outcomes. Learning Environments Research, 1-18.

Bonneville-Roussy, A., Vallerand, R. J., & Bouffard, T. (2013). The roles of autonomy support and harmonious and obsessive passions in educational persistence. Learning and Individual Differences, 24, 22-31.

Brambilla, M., Assor, A., Manzi, C., & Regalia, C. (2015). Autonomous versus controlled religiosity: Family and group antecedents. The International Journal for the Psychology of Religion, 25(3), 193-210.

Brandišauskienė, A., Česnavičienė, J., Bruzgelevičienė, R., & Nedzinskaitė-Mačiūnienė, R. (2021). Connections between teachers’ motivational behaviour and school student engagement. Electronic journal of research in educational psychology. Almeria: University of Almeria, 2021, vol. 19, no. 53.

Brenning, K., Soenens, B., Braet, C., & Bal, S. (2012). The role of parenting and mother-adolescent attachment in the intergenerational similarity of internalizing symptoms. Journal of Youth and Adolescence, 41(6), 802-816.

Bureau, J. S., & Mageau, G. A. (2014). Parental autonomy support and honesty: The mediating role of identification with the honesty value and perceived costs and benefits of honesty. Journal of Adolescence, 37(3), 225-236.

Burgueño, R., Macarro-Moreno, J., & Medina-Casaubón, J. (2020). Psychometry of the multidimensional perceived autonomy support scale in physical education with spanish secondary school students. Sage Open, 10(1), 2158244019901253.

Burgueño, R., Abós, Á., García-González, L., Tilga, H., & Sevil-Serrano, J. (2021). Evaluating the psychometric properties of a scale to measure perceived external and internal faces of controlling teaching among students in physical education. International Journal of Environmental Research and Public Health, 18(1), 298.

Burgueño, R., & Medina-Casaubón, J. (2021). Validity and reliability of the interpersonal behaviors questionnaire in physical education with Spanish secondary school students. Perceptual and Motor Skills, 128(1), 522-545.

Burgueño, R., García-González, L., Abós, Á., & Sevil-Serrano, J. (2022). Students’ motivational experiences across profiles of perceived need-supportive and need-thwarting teaching behaviors in physical education. Physical Education and Sport Pedagogy, 1-15.

Bush, A. M. (2006). What comes between classroom community and academic emotions: Testing a self-determination model of motivation in the college classroom (Doctoral dissertation).

Buzzai, C., Sorrenti, L., Orecchio, S., Marino, D., & Filippello, P. (2020). The relationship between contextual and dispositional variables, well-being and hopelessness in school context. Frontiers in Psychology, 11, 533815.

Buzzai, C., Filippello, P., Costa, S., Amato, V., & Sorrenti, L. (2021). Problematic internet use and academic achievement: A focus on interpersonal behaviours and academic engagement. Social Psychology of Education, 24, 95-118.

Need-supportive and need-thwarting interpersonal behaviors by teachers and classmates in adolescence: The mediating role of basic psychological needs on school alienation and academic achievement

Caleon, I. S., Tan, J. P. L., Wui, M. G. L., Leen, C. C., & King, R. B. (2016). Academically at-risk adolescents in Singapore: The importance of teacher support in promoting academic engagement. In The psychology of Asian learners (pp. 519-539). Springer, Singapore.

Cappetta, R., & Paolino, C. (2015). Is It Always Worth Waiting? The Effect of Autonomy‐supportive Teaching on Short‐term and Long‐term Learning Outcomes. British Journal of Management, 26(1), 93-108.

Carbonneau, N., Carbonneau, E., Cantin, M., & Gagnon-Girouard, M. P. (2015). Examining women's perceptions of their mother's and romantic partner's interpersonal styles for a better understanding of their eating regulation and intuitive eating. Appetite, 92, 156-166.

Carr, V. M., Francis, A. P., & Wieth, M. B. (2021). The Relationship Between Helicopter Parenting and Fear of Negative Evaluation in College Students. Journal of Child and Family Studies, 30(8), 1910-1919.

Carreira, J. M., Ozaki, K., & Maeda, T. (2013). Motivational model of English learning among elementary school students in Japan. System, 41(3), 706-719.

Caruso, A., Grolnick, W., Rabner, J., & Lebel, A. (2019). Parenting, self-regulation, and treatment adherence in pediatric chronic headache: A self-determination theory perspective. Journal of Health Psychology, 1359105319884596.

Chan, D. K. C., Yang, S. X., Mullan, B., Du, X., Zhang, X., Chatzisarantis, N. L., & Hagger, M. S. (2015). Preventing the spread of H1N1 influenza infection during a pandemic: autonomy-supportive advice versus controlling instruction. Journal of behavioral medicine, 38(3), 416-426.

Chatzisarantis, N. L., & Hagger, M. S. (2009). Effects of an intervention based on self-determination theory on self-reported leisure-time physical activity participation. Psychology and Health, 24(1), 29-48.

Chatzisarantis, N. L., Hagger, M. S., Wang, C. J., & Thøgersen-Ntoumani, C. (2009). The effects of social identity and perceived autonomy support on health behaviour within the theory of planned behaviour. Current Psychology, 28(1), 55-68.

Chatzisarantis, N. L., Ada, E. N., Ahmadi, M., Caltabiano, N., Wang, D., Thogersen-Ntoumani, C., & Hagger, M. S. (2019). Differential effects of perceptions of equal, favourable and unfavourable autonomy support on educational and well-being outcomes. Contemporary Educational Psychology, 58, 33-43.

Chen, K. C., & Jang, S. J. (2010). Motivation in online learning: Testing a model of self-determination theory. Computers in Human Behavior, 26(4), 741-752.

Chen, C., Zhang, T., Gu, X., Lee, J., Ren, S., & Wang, H. (2020). Understanding Adolescents’ Need Support, Need Satisfaction, and Health-Related Outcomes: A Self-Determination Health Behavior Perspective. International Journal of Environmental Research and Public Health, 17(1), 104.

Chen, P., Zhang, J., Li, H., & Fu, M. (2021). Relationships between Parenting Behaviors and Adolescents’ Creativity in China: The Mediating Role of Autonomous Motivation. The Journal of Psychology, 155(5), 457-472.

Chen, P., Zhang, J., Xu, N., Zhang, K., & Xiao, L. (2021). The relationship between need for cognition and adolescents’ creative self-efficacy: The mediating roles of perceived parenting behaviors and perceived teacher support. Current Psychology, 1-14.

Cheon, S. H., Reeve, J., & Ntoumanis, N. (2018). A needs-supportive intervention to help PE teachers enhance students' prosocial behavior and diminish antisocial behavior. Psychology of Sport and Exercise, 35, 74-88.

Chen, Y., Li, R., & Liu, X. (2019). The relationships among parental psychological control/autonomy support, self‐trouble, and internalizing problems across adolescent genders. Scandinavian journal of psychology, 60(6), 539-547.

Chen, C., Elliot, A. J., & Sheldon, K. M. (2019). Psychological need support as a predictor of intrinsic and external motivation: the mediational role of achievement goals. Educational Psychology, 39(8), 1090-1113.

Cheung, C. S. S., Pomerantz, E. M., & Dong, W. (2013). Does adolescents' disclosure to their parents matter for their academic adjustment?. Child Development, 84(2), 693-710.

Chew, E., & Wang, J. (2010). Perceptions of parental autonomy support and control, and aspirations of student athletes in Singapore. In Sport science and studies in Asia: Issues, reflections and emergent solutions (pp. 231-248).

Chirkov, V. I., & Ryan, R. M. (2001). Parent and teacher autonomy-support in Russian and US adolescents: Common effects on well-being and academic motivation. Journal of cross-cultural psychology, 32(5), 618-635.

Choe, S. Y., & Read, S. J. (2019). Perceived parental psychological control has indirect effects on aggression via need satisfaction and motivation for revenge. Journal of social and personal relationships, 36(9), 2857-2879.

Christiana, R. W., Davis, M., Wilson, M. G., McCarty, F. A., & Green, G. T. (2014). Factors related to rural young adolescents' participation in outdoor, noncompetitive physical activity. Research quarterly for exercise and sport, 85(4), 509-518.

Chu, T. L., Zhang, T., & Cheung, H. Y. (2019). The roles of need-supportive social environments in university physical education courses. International Journal of Sport and Exercise Psychology, 17(3), 212-231.

Ciani, K. D., Summers, J. J., Easter, M. A., & Sheldon, K. M. (2008). Collaborative learning and positive experiences: does letting students choose their own groups matter?. Educational Psychology, 28(6), 627-641.

Ciani, K. D., Middleton, M. J., Summers, J. J., & Sheldon, K. M. (2010). Buffering against performance classroom goal structures: The importance of autonomy support and classroom community. Contemporary educational psychology, 35(1), 88-99.

Ciani, K. D., Sheldon, K. M., Hilpert, J. C., & Easter, M. A. (2011). Antecedents and trajectories of achievement goals: A self‐determination theory perspective. British Journal of Educational Psychology, 81(2), 223-243.

Cimon-Paquet, C., Bernier, A., Matte-Gagné, C., & Mageau, G. A. (2020). Early maternal autonomy support and mathematical achievement trajectories during elementary school. Learning and Individual Differences, 79, 101855.

Clark, K. E., & Ladd, G. W. (2000). Connectedness and autonomy support in parent–child relationships: Links to children's socioemotional orientation and peer relationships. Developmental psychology, 36(4), 485.

Codina, N., Valenzuela, R., Pestana, J. V., & Gonzalez-Conde, J. (2018). Relations between student procrastination and teaching styles: Autonomy-supportive and controlling. Frontiers in psychology, 9, 809.

Codina, N., Castillo, I., Pestana, J. V., & Balaguer, I. (2020). Preventing procrastination behaviours: Teaching styles and competence in university students. Sustainability, 12(6), 2448.

Collie, R. J., Granziera, H., & Martin, A. J. (2019). Teachers' motivational approach: Links with students’ basic psychological need frustration, maladaptive engagement, and academic outcomes. Teaching and Teacher Education, 86, 102872.

Charlot Colomès, A. A., Duchesne, S., & Boisclair Châteauvert, G. (2021). Autonomy support and school adjustment: The mediating role of basic psychological needs. International Journal of School & Educational Psychology, 1-19.

Cordeiro, P. M. G., Paixao, M. P., Lens, W., Lacante, M., & Luyckx, K. (2018). Parenting styles, identity development, and adjustment in career transitions: The mediating role of psychological needs. Journal of Career Development, 45(1), 83-97.

Corwyn, R. F., & Bradley, R. H. (2016). Fathers' autonomy support and social competence of sons and daughters. Merrill-Palmer Quarterly, 62(4), 359-387.

Costa, S., Soenens, B., Gugliandolo, M. C., Cuzzocrea, F., & Larcan, R. (2015). The mediating role of experiences of need satisfaction in associations between parental psychological control and internalizing problems: A study among Italian college students. Journal of Child and Family Studies, 24(4), 1106-1116.

Costa, S., Cuzzocrea, F., Gugliandolo, M. C., & Larcan, R. (2016). Associations between parental psychological control and autonomy support, and psychological outcomes in adolescents: The mediating role of need satisfaction and need frustration. Child Indicators Research, 9(4), 1059-1076.

Costa, S., Gugliandolo, M. C., Barberis, N., Cuzzocrea, F., & Liga, F. (2019). Antecedents and consequences of parental psychological control and autonomy support: The role of psychological basic needs. Journal of Social and Personal Relationships, 36(4), 1168-1189.

Costa, S., Sireno, S., Larcan, R., & Cuzzocrea, F. (2019). The six dimensions of parenting and adolescent psychological adjustment: The mediating role of psychological needs. Scandinavian journal of psychology, 60(2), 128-137.

Costa, S., Barberis, N., Gugliandolo, M. C., Larcan, R., & Cuzzocrea, F. (2018). The intergenerational transmission of trait emotional intelligence: The mediating role of parental autonomy support and psychological control. Journal of Adolescence, 68, 105-116.

Cronin, L. D., Allen, J., Mulvenna, C., & Russell, P. (2018). An investigation of the relationships between the teaching climate, students’ perceived life skills development and well-being within physical education. Physical Education and Sport Pedagogy, 23(2), 181-196.

Cronin, L., Marchant, D., Allen, J., Mulvenna, C., Cullen, D., Williams, G., & Ellison, P. (2019). Students’ perceptions of autonomy-supportive versus controlling teaching and basic need satisfaction versus frustration in relation to life skills development in PE. Psychology of Sport and Exercise, 44, 79-89.

Cronin, L., Marchant, D., Johnson, L., Huntley, E., Kosteli, M. C., Varga, J., & Ellison, P. (2020). Life skills development in physical education: A self-determination theory-based investigation across the school term. Psychology of Sport and Exercise, 101711.

Cui, G., Yao, M., & Zhang, X. (2017). The dampening effects of perceived teacher enthusiasm on class-related boredom: The mediating role of perceived autonomy support and task value. Frontiers in psychology, 8, 400.

De Loof, H., Struyf, A., Boeve-de Pauw, J., & Van Petegem, P. (2019). Teachers’ motivating style and students’ motivation and engagement in STEM: The relationship between three key educational concepts. Research in Science Education, 1-19.

De Meyer, J., Soenens, B., Aelterman, N., De Bourdeaudhuij, I., & Haerens, L. (2016). The different faces of controlling teaching: implications of a distinction between externally and internally controlling teaching for students’ motivation in physical education. Physical Education and Sport Pedagogy, 21(6), 632-652.

Delahaij, R., Theunissen, N. C., & Six, C. (2014). The influence of autonomy support on self-regulatory processes and attrition in the Royal Dutch Navy. Learning and Individual Differences, 30, 177-181.

GÖNÜL, T. D., İBRAHIM, C. H., Levent, I. E., & Zekihan, H. (2018). EXAMINATION OF FRIEND RELATED AUTONOMY IN THE PARTICIPATION OF PHYSICAL EDUCATION TEACHER CANDIDATES IN THE EXERCISE. Ovidius University Annals, Series Physical Education and Sport/Science, Movement and Health, 18(2 S1), 452-458.

Demir, M., Burton, S., & Dunbar, N. (2019). Professor–student rapport and perceived autonomy support as predictors of course and student outcomes. Teaching of Psychology, 46(1), 22-33.

Denault, A. S., & Guay, F. (2017). Motivation towards extracurricular activities and motivation at school: A test of the generalization effect hypothesis. Journal of Adolescence, 54, 94-103.

Depestele, L., Soenens, B., Lemmens, G. M., Dierckx, E., Schoevaerts, K., & Claes, L. (2017). Parental autonomy-support and psychological control in eating disorder patients with and without binge-eating/purging behavior and non-suicidal self-injury. Journal of Social and Clinical Psychology, 36(2), 126-141.

Dettweiler, U., Lauterbach, G., Becker, C., & Simon, P. (2017). A bayesian mixed-methods analysis of basic psychological needs satisfaction through outdoor learning and its influence on motivational behavior in science class. Frontiers in psychology, 8, 2235.

Deventer, J., Humberg, S., Lüdtke, O., Nagy, G., Retelsdorf, J., & Wagner, J. (2019). Testing Competing Hypotheses on the Interplay of Importance and Support of the Basic Psychological Needs at Work and Personality Development with Response Surface Analysis. Collabra: Psychology, 5(1).

Dieleman, L. M., Soenens, B., Prinzie, P., De Clercq, L., Ortibus, E., & De Pauw, S. S. (2021). Daily parenting of children with cerebral palsy: The role of daily child behavior, parents’ daily psychological needs, and mindful parenting. Development and psychopathology, 33(1), 184-200.

Dieleman, L. M., Soenens, B., Vansteenkiste, M., Prinzie, P., Laporte, N., & De Pauw, S. S. (2019). Daily sources of autonomy-supportive and controlling parenting in mothers of children with ASD: The role of child behavior and mothers’ psychological needs. Journal of autism and developmental disorders, 49(2), 509-526.

Dincer, A., Yeşilyurt, S., Noels, K. A., & Vargas Lascano, D. I. (2019). Self-determination and classroom engagement of EFL learners: A mixed-methods study of the self-system model of motivational development. SAGE Open, 9(2), 2158244019853913.

Diseth, Å., Danielsen, A. G., & Samdal, O. (2012). A path analysis of basic need support, self-efficacy, achievement goals, life satisfaction and academic achievement level among secondary school students. Educational Psychology, 32(3), 335-354.

Diseth, Å., Breidablik, H. J., & Meland, E. (2018). Longitudinal relations between perceived autonomy support and basic need satisfaction in two student cohorts. Educational Psychology, 38(1), 99-115.

Domen, J., Hornstra, L., Weijers, D., van der Veen, I., & Peetsma, T. (2020). Differentiated need support by teachers: Student‐specific provision of autonomy and structure and relations with student motivation. British Journal of Educational Psychology, 90(2), 403-423.

Downie, M., Chua, S. N., Koestner, R., Barrios, M. F., Rip, B., & M'Birkou, S. (2007). The relations of parental autonomy support to cultural internalization and well-being of immigrants and sojourners. Cultural Diversity and Ethnic Minority Psychology, 13(3), 241.

Duarte-Félix, H., Zamarripa, J., Baños, R., de la Cruz-Ortega, M., & Delgado-Herrada, M. (2020). Psychometric Properties of the Interpersonal Styles Questionnaire for Physical Education in a Mexican Sample. International Journal of Environmental Research and Public Health, 17(18), 6636.

Dubnjakovic, A. (2018). Antecedents and consequences of autonomous information seeking motivation. Library & Information Science Research, 40(1), 9-17.

Duchatelet, D., & Donche, V. (2019). Fostering self-efficacy and self-regulation in higher education: a matter of autonomy support or academic motivation?. Higher Education Research & Development, 38(4), 733-747.

Duineveld, J. J., Parker, P. D., Ryan, R. M., Ciarrochi, J., & Salmela-Aro, K. (2017). The link between perceived maternal and paternal autonomy support and adolescent well-being across three major educational transitions. Developmental psychology, 53(10), 1978.

Dupont, S., Galand, B., Nils, F., & Hospel, V. (2014). Social context, self-perceptions and student engagement: A SEM investigation of the self-system model of motivational development (SSMMD).

Eakman, A. M., Kinney, A. R., Schierl, M. L., & Henry, K. L. (2019). Academic performance in student service members/veterans: Effects of instructor autonomy support, academic self-efficacy and academic problems. Educational Psychology, 39(8), 1005-1026.

Egeli, N. A., Rogers, W. T., Rinaldi, C. M., & Cui, Y. (2015). Exploring the factor structure of the revised-parent as a social context questionnaire. Parenting, 15(4), 269-287.

Ekatushabe, M., Kwarikunda, D., Muwonge, C. M., Ssenyonga, J., & Schiefele, U. (2021). Relations between perceived teacher’s autonomy support, cognitive appraisals and boredom in physics learning among lower secondary school students. International Journal of STEM Education, 8(1), 1-15.

Emeljanovas, A., Mieziene, B., Putriute, V., Sinkariova, L., Tilindiene, I., & Trinkuniene, L. (2020). The Relationship Between Objectively Measured Class Physical Activity and Teachers' Autonomy Supportive and Controlling Behaviors. Revista de Psicología del Deporte, 29.

Emery, A. A. (2016). Applying Self-Determination Theory to Further Our Understanding of Non-Suicidal Self-Injury. McGill University (Canada).

Escriva-Boulley, G., Tessier, D., Ntoumanis, N., & Sarrazin, P. (2018). Need-supportive professional development in elementary school physical education: Effects of a cluster-randomized control trial on teachers’ motivating style and student physical activity. Sport, Exercise, and Performance Psychology, 7(2), 218.

Escriva-Boulley, G., Descas, E. G., Aelterman, N., Vansteenkiste, M., Van Doren, N., Lentillon-Kaestner, V., & Haerens, L. (2021). Adopting the Situation in School Questionnaire to Examine Physical Education Teachers’ Motivating and Demotivating Styles Using a Circumplex Approach.

Escriva-Boulley, G., Guillet-Descas, E., Aelterman, N., Vansteenkiste, M., Van Doren, N., Lentillon-Kaestner, V., & Haerens, L. (2021). Adopting the situation in school questionnaire to examine physical education teachers’ motivating and demotivating styles using a circumplex approach. International Journal of Environmental Research and Public Health, 18(14), 7342.

Feng, L., & Lan, X. (2020). The moderating role of autonomy support profiles in the association between grit and externalizing problem behavior among family-bereaved adolescents. Frontiers in Psychology, 11.

Feng, X., Xie, K., Gong, S., Gao, L., & Cao, Y. (2019). Effects of parental autonomy support and teacher support on middle school students’ homework effort: homework autonomous motivation as mediator. Frontiers in psychology, 10, 612.

Ferguson, Y. L., Kasser, T., & Jahng, S. (2011). Differences in life satisfaction and school satisfaction among adolescents from three nations: The role of perceived autonomy support. Journal of research on Adolescence, 21(3), 649-661.

Ferrari, L., Manzi, C., Benet-Martinez, V., & Rosnati, R. (2019). Social and Family Factors Related to Intercountry Adoptees and Immigrants’ Bicultural Identity Integration. Journal of Cross-Cultural Psychology, 50(6), 789-805.

Fikkers, K. M., Piotrowski, J. T., & Valkenburg, P. M. (2017). A matter of style? Exploring the effects of parental mediation styles on early adolescents’ media violence exposure and aggression. Computers in Human Behavior, 70, 407-415.

Filippello, P., Larcan, R., Sorrenti, L., Buzzai, C., Orecchio, S., & Costa, S. (2017). The mediating role of maladaptive perfectionism in the association between psychological control and learned helplessness. Improving Schools, 20(2), 113-126.

Filippello, P., Harrington, N., Costa, S., Buzzai, C., & Sorrenti, L. (2018). Perceived parental psychological control and school learned helplessness: The role of frustration intolerance as a mediator factor. School Psychology International, 39(4), 360-377.

Filippello, P., Buzzai, C., Costa, S., & Sorrenti, L. (2019). School refusal and absenteeism: Perception of teacher behaviors, psychological basic needs, and academic achievement. Frontiers in Psychology, 10, 1471.

Filippello, P., Buzzai, C., Costa, S., Orecchio, S., & Sorrenti, L. (2020). Teaching style and academic achievement: The mediating role of learned helplessness and mastery orientation. Psychology in the Schools, 57(1), 5-16.

Fin, G., Moreno-Murcia, J. A., León, J., Baretta, E., & Nodari Júnior, R. J. (2019). Teachers' interpersonal style in physical education: Exploring patterns of students' self-determined motivation and enjoyment of physical activity in a longitudinal study. Frontiers in Psychology, 9(JAN). doi:10.3389/fpsyg.2018.02721

Fousiani, K., Van Petegem, S., Soenens, B., Vansteenkiste, M., & Chen, B. (2014). Does parental autonomy support relate to adolescent autonomy? An in-depth examination of a seemingly simple question. Journal of Adolescent Research, 29(3), 299-330.

Fousiani, K., Dimitropoulou, P., Michaelides, M. P., & Van Petegem, S. (2016). Perceived parenting and adolescent cyber-bullying: Examining the intervening role of autonomy and relatedness need satisfaction, empathic concern and recognition of humanness. Journal of child and family studies, 25(7), 2120-2129.

Franco, E., Coterón, J., Gómez, V., & Spray, C. M. (2021). A person-centred approach to understanding dark-side antecedents and students’ outcomes associated with physical education teachers’ motivation. Psychology of Sport and Exercise, 57, 102021.

Freer, E., & Evans, P. (2019). Choosing to study music in high school: Teacher support, psychological needs satisfaction, and elective music intentions. Psychology of Music, 47(6), 781-799.

Froiland, J. M., & Worrell, F. C. (2017). Parental autonomy support, community feeling and student expectations as contributors to later achievement among adolescents. Educational Psychology, 37(3), 261-271.

Furtak, E. M., & Kunter, M. (2012). Effects of autonomy-supportive teaching on student learning and motivation. The Journal of Experimental Education, 80(3), 284-316.

Gagne, M., Ryan, R. M., & Bargmann, K. (2003). Autonomy support and need satisfaction in the motivation and well-being of gymnasts. Journal of applied sport psychology, 15(4), 372-390.

Gagné, M. (2003). The role of autonomy support and autonomy orientation in prosocial behavior engagement. Motivation and emotion, 27(3), 199-223.

Gagnon, É., Ratelle, C. F., Guay, F., & Duchesne, S. (2019). Developmental trajectories of vocational exploration from adolescence to early adulthood: The role of parental need supporting behaviors. Journal of Vocational Behavior, 115, 103338.

Garn, A. C., McCaughtry, N., Martin, J., Shen, B., & Fahlman, M. (2012). A Basic Needs Theory investigation of adolescents' physical self-concept and global self-esteem. International Journal of Sport and Exercise Psychology, 10(4), 314-328. doi:10.1080/1612197X.2012.705521

Garn, A. C., Morin, A. J., & Lonsdale, C. (2019). Basic psychological need satisfaction toward learning: A longitudinal test of mediation using bifactor exploratory structural equation modeling. Journal of Educational Psychology, 111(2), 354.

George, S. V., & Richardson, P. W. (2019). Teachers’ goal orientations as predictors of their self-reported classroom behaviours: An achievement goal theoretical perspective. International Journal of Educational Research, 98, 345-355.

Gilbert, W., Bureau, J. S., Poellhuber, B., & Guay, F. (2021). Predicting college students' psychological distress through basic psychological need-relevant practices by teachers, peers, and the academic program. Motivation and Emotion, 1-20.

Gillet, N., Berjot, S., Vallerand, R. J., & Amoura, S. (2012). The role of autonomy support and motivation in the prediction of interest and dropout intentions in sport and education settings. Basic and Applied Social Psychology, 34(3), 278-286.

Gillet, N., Vallerand, R. J., & Lafrenière, M. A. K. (2012). Intrinsic and extrinsic school motivation as a function of age: The mediating role of autonomy support. Social Psychology of Education, 15(1), 77-95.

Girelli, L., Hagger, M., Mallia, L., & Lucidi, F. (2016). From perceived autonomy support to intentional behaviour: testing an integrated model in three healthy-eating behaviours. Appetite, 96, 280-292.

Goethals, E. R., Soenens, B., de Wit, M., Vansteenkiste, M., Laffel, L. M., Casteels, K., & Luyckx, K. (2019). “Let's talk about it” The role of parental communication in adolescents' motivation to adhere to treatment recommendations for type 1 diabetes. Pediatric diabetes, 20(7), 1025-1034.

Gong, X., & Wang, C. (2021). Interactive effects of parental psychological control and autonomy support on emerging adults’ emotion regulation and self-esteem. Current Psychology, 1-10.

Gonida, E. N., & Cortina, K. S. (2014). Parental involvement in homework: Relations with parent and student achievement‐related motivational beliefs and achievement. British Journal of Educational Psychology, 84(3), 376-396.

González, A., & Paoloni, P. V. (2015). Perceived autonomy-support, expectancy, value, metacognitive strategies and performance in chemistry: a structural equation model in undergraduates. Chemistry Education Research and Practice, 16(3), 640-653.

González-Cutre, D., Ferriz, R., Beltrán-Carrillo, V. J., Andrés-Fabra, J. A., Montero-Carretero, C., Cervelló, E., & Moreno-Murcia, J. A. (2014). Promotion of autonomy for participation in physical activity: A study based on the trans-contextual model of motivation. Educational Psychology, 34(3), 367-384.

González-Cutre, D., Sierra, A. C., Beltrán-Carrillo, V. J., Peláez-Pérez, M., & Cervelló, E. (2018). A school-based motivational intervention to promote physical activity from a self-determination theory perspective. The Journal of Educational Research, 111(3), 320-330.

González-Peño, A., Franco, E., & Coterón, J. (2021). Do Observed Teaching Behaviors Relate to Students’ Engagement in Physical Education?. International journal of environmental research and public health, 18(5), 2234.

Graça, J., Calheiros, M. M., & Barata, M. C. (2013). Authority in the classroom: adolescent autonomy, autonomy support, and teachers’ legitimacy. European journal of psychology of education, 28(3), 1065-1076.

Granero-Gallegos, A., Baena-Extremera, A., Sánchez-Fuentes, J. A., & Martínez-Molina, M. (2014). Motivational profiles of autonomy support, self-determination, satisfaction, importance of physical education and intention to partake in leisure time physical activity. Cuadernos de Psicología del Deporte, 14(2), 59-69.

Greene, B. A., Miller, R. B., Crowson, H. M., Duke, B. L., & Akey, K. L. (2004). Predicting high school students' cognitive engagement and achievement: Contributions of classroom perceptions and motivation. Contemporary educational psychology, 29(4), 462-482.

Greene, N. R., Jewell, D. E., Fuentes, J. D., & Smith, C. V. (2019). Basic need satisfaction in the parental relationship offsets millennials’ worries about the transition to college. The Journal of social psychology, 159(2), 125-137.

Griffin, B. W. (2016). Perceived autonomy support, intrinsic motivation, and student ratings of instruction. Studies in Educational Evaluation, 51, 116-125.

Griffith, S. F., & Grolnick, W. S. (2014). Parenting in Caribbean families: A look at parental control, structure, and autonomy support. Journal of Black Psychology, 40(2), 166-190.

Grolnick, W. S., Kurowski, C. O., Dunlap, K. G., & Hevey, C. (2000). Parental resources and the transition to junior high. Journal of Research on Adolescence, 10(4), 465-488.

Guay, F., Boggiano, A. K., & Vallerand, R. J. (2001). Autonomy support, intrinsic motivation, and perceived competence: Conceptual and empirical linkages. Personality and Social Psychology Bulletin, 27(6), 643-650.

Guay, F., Ratelle, C., Larose, S., Vallerand, R. J., & Vitaro, F. (2013). The number of autonomy-supportive relationships: Are more relationships better for motivation, perceived competence, and achievement?. Contemporary Educational Psychology, 38(4), 375-382.

Guay, F., Ratelle, C. F., Duchesne, S., & Dubois, P. (2018). Mothers’ and fathers’ autonomy-supportive and controlling behaviors: An analysis of interparental contributions. Parenting, 18(1), 45-65.

Gucciardi, D. F., Weixian, J. C., Gibson, W., Ntoumanis, N., & Ng, L. (2019). Motivational climate in the classroom: Factorial and convergent validity evidence of the Need-Supportive Behaviors Scale with health science students. European Journal of Psychological Assessment.

Gueta, B., & Berkovich, I. (2021). The effect of autonomy-supportive climate in a second chance programme for at-risk youth on dropout risk: the mediating role of adolescents’ sense of authenticity. European Journal of Psychology of Education, 1-16.

Guo, M., Wang, L., Day, J., & Chen, Y. (2021). The Relations of Parental Autonomy Support, Parental Control, and Filial Piety to Chinese Adolescents’ Academic Autonomous Motivation: A Mediation Model. Frontiers in Psychology, 12.

Gurland, S. T., & Evangelista, J. E. (2015). Teacher–student relationship quality as a function of children’s expectancies. Journal of social and personal relationships, 32(7), 879-904.

Gutiérrez, M., & Tomás, J. M. (2018). Motivational class climate, motivation and academic success in university students. Revista de Psicodidáctica (English ed.), 23(2), 94-101.

Gutiérrez, M., & Tomás, J. M. (2019). The role of perceived autonomy support in predicting university students’ academic success mediated by academic self-efficacy and school engagement. Educational Psychology, 39(6), 729-748.

Gutiérrez, M., Sancho, P., Galiana, L., & Tomás, J. M. (2018). Autonomy support, psychological needs satisfaction, school engagement and academic success: A mediation model. Universitas Psychologica, 17(5), 1-12.

Haerens, L., Aelterman, N., Van den Berghe, L., De Meyer, J., Soenens, B., & Vansteenkiste, M. (2013). Observing physical education teachers’ need-supportive interactions in classroom settings. Journal of Sport and Exercise Psychology, 35(1), 3-17.

Haerens, L., Aelterman, N., Vansteenkiste, M., Soenens, B., & Van Petegem, S. (2015). Do perceived autonomy-supportive and controlling teaching relate to physical education students' motivational experiences through unique pathways? Distinguishing between the bright and dark side of motivation. Psychology of sport and exercise, 16, 26-36.

Haerens, L., Vansteenkiste, M., De Meester, A., Delrue, J., Tallir, I., Vande Broek, G., ... & Aelterman, N. (2018). Different combinations of perceived autonomy support and control: Identifying the most optimal motivating style. Physical Education and Sport Pedagogy, 23(1), 16-36.

Hagger, M. S., & Hamilton, K. (2018). Motivational predictors of students' participation in out-of-school learning activities and academic attainment in science: An application of the trans-contextual model using Bayesian path analysis. Learning and Individual Differences, 67, 232-244.

Hagger, M. S., Culverhouse, T., Chatzisarantis, N. L. D., & Biddle, S. J. H. (2003). The Processes by Which Perceived Autonomy Support in Physical Education Promotes Leisure-Time Physical Activity Intentions and Behavior: A Trans-Contextual Model. Journal of Educational Psychology, 95(4), 784-795. doi:10.1037/0022-0663.95.4.794

Hagger, M. S., Chatzisarantis, N. L., Barkoukis, V., Wang, C. K. J., & Baranowski, J. (2005). Perceived autonomy support in physical education and leisure-time physical activity: a cross-cultural evaluation of the trans-contextual model. Journal of educational Psychology, 97(3), 376.

Hagger, M. S., Chatzisarantis, N. L. D., Hein, V., Pihu, M., Soós, I., & Karsai, I. (2007). The perceived autonomy support scale for exercise settings (PASSES): Development, validity, and cross-cultural invariance in young people. Psychology of Sport and Exercise, 8(5), 632-653. doi:10.1016/j.psychsport.2006.09.001

Hagger, M., Chatzisarantis, N. L. D., Hein, V., Soós, I., Karsai, I., Lintunen, T., & Leemans, S. (2009). Teacher, peer and Autonomy Support (parent) in physical education and leisure-time physical activity: A trans-contextual model of motivation in four nations. Psychology and Health, 24(6), 689-711. doi:10.1080/08870440801956192

Hagger, M. S., Sultan, S., Hardcastle, S. J., & Chatzisarantis, N. L. (2015). Perceived autonomy support and autonomous motivation toward mathematics activities in educational and out-of-school contexts is related to mathematics homework behavior and attainment. Contemporary Educational Psychology, 41, 111-123.

Hall, N., & Webb, D. (2014). Instructors’ support of student autonomy in an introductory physics course. Physical Review Special Topics-Physics Education Research, 10(2), 020116.

Hang, B. T. T., Kaur, A., & Nur, A. H. B. (2017). A Self-Determination Theory Based Motivational Model on Intentions to Drop out of Vocational Schools in Vietnam. Malaysian Journal of Learning and Instruction, 14(1), 1-21.

Hanna, K. M., Dashiff, C. J., Stump, T. E., & Weaver, M. T. (2013). Parent–adolescent dyads: association of parental autonomy support and parent–adolescent shared diabetes care responsibility. Child: care, health and development, 39(5), 695-702.

Hansen, D. M., Moore, E. W., & Jessop, N. (2018). Youth Program Adult Leader's Directive Assistance and Autonomy Support and Development of Adolescents’ Agency Capacity. Journal of Research on Adolescence, 28(2), 505-519.

Hardre, P. L., & Reeve, J. (2003). A motivational model of rural students' intentions to persist in, versus drop out of, high school. Journal of educational psychology, 95(2), 347.

Hardy, S. A., Padilla‐Walker, L. M., & Carlo, G. (2008). Parenting dimensions and adolescents' internalisation of moral values. Journal of Moral Education, 37(2), 205-223.

Hein, V., & Caune, A. (2014). Relationships between perceived teacher’s autonomy support, effort and physical self-esteem. Kinesiology: International journal of fundamental and applied kinesiology, 46(2), 218-226.

Hein, V., Emeljanovas, A., Ries, F., Valantine, I., Ekler, J. H., & López, P. G. (2018). The perception of the autonomy supportive behaviour as a predictor of perceived effort and physical self-esteem among school students from four nations. Montenegrin Journal of Sports Science and Medicine, 7(1), 21.

Hein, V., Koka, A., & Hagger, M. S. (2015). Relationships between perceived teachers' controlling behaviour, psychological need thwarting, anger and bullying behaviour in high-school students. Journal of adolescence, 42, 103-114.

Herrera, D., Matos, L., Gargurevich, R., Lira, B., & Valenzuela, R. (2021). Context Matters: Teaching Styles and Basic Psychological Needs Predicting Flourishing and Perfectionism in University Music Students. Frontiers in Psychology, 12, 438.

Hindman, A. H., & Morrison, F. J. (2012). Differential contributions of three parenting dimensions to preschool literacy and social skills in a middle-income sample. Merrill-Palmer Quarterly (1982-), 191-223.

Hope, N. H., & Chapman, A. L. (2019). Difficulties regulating emotions mediates the associations of parental psychological control and emotion invalidation with borderline personality features. Personality Disorders: Theory, Research, and Treatment, 10(3), 267.

Hornstra, L., Stroet, K., & Weijers, D. (2021). Profiles of teachers’ need-support: How do autonomy support, structure, and involvement cohere and predict motivation and learning outcomes?. Teaching and Teacher Education, 99, 103257.

Hornstra, L., Stroet, K., van Eijden, E., Goudsblom, J., & Roskamp, C. (2018). Teacher expectation effects on need-supportive teaching, student motivation, and engagement: a self-determination perspective. Educational Research and Evaluation, 24(3-5), 324-345.

Hornstra, L., Bakx, A., Mathijssen, S., & Denissen, J. J. (2020). Motivating gifted and non-gifted students in regular primary schools: A self-determination perspective. Learning and Individual Differences, 80, 101871.

How, Y. M., Whipp, P., Dimmock, J., & Jackson, B. (2013). The effects of choice on autonomous motivation, perceived autonomy support, and physical activity levels in high school physical education. Journal of teaching in physical education, 32(2), 131-148.

Hu, N., Yuan, M., Liu, J., Coplan, R. J., & Zhou, Y. (2021). Examining Reciprocal Links between Parental Autonomy-Support and Children’s Peer Preference in Mainland China. Children, 8(6), 508.

Huéscar Hernández, E., Moreno-Murcia, J. A., Ruíz González, L., & León González, J. (2019). Motivational Profiles of High School Physical Education Students: The Role of Controlling Teacher Behavior. International Journal of Environmental Research and Public Health, 16(10), 1714. doi:10.3390/ijerph16101714

Huéscar Hernández, E., Moreno-Murcia, J. A., Cid, L., Monteiro, D., & Rodrigues, F. (2020). Passion or perseverance? The effect of perceived autonomy support and grit on academic performance in college students. International Journal of Environmental Research and Public Health, 17(6), 2143.

Hughes, C., Lindberg, A., & Devine, R. T. (2018). Autonomy support in toddlerhood: Similarities and contrasts between mothers and fathers. Journal of Family Psychology, 32(7), 915.

Iglesias García, M. T., Maulana, R., Fernández García, C. M., & García Pérez, O. (2020). Teacher as social context (TASC) questionnaire in the Spanish setting: Teacher version. Psicología Educativa, 26.

Li, R., Yao, M., Chen, Y., & Liu, H. (2020). Parent Autonomy Support and Psychological Control, Dark Triad, and Subjective Well-Being of Chinese Adolescents: Synergy of Variable-and Person-Centered Approaches. The Journal of Early Adolescence, 40(7), 966-995.

Inguglia, C., Ingoglia, S., Liga, F., Coco, A. L., & Cricchio, M. G. L. (2015). Autonomy and relatedness in adolescence and emerging adulthood: Relationships with parental support and psychological distress. Journal of Adult Development, 22(1), 1-13.

Inguglia, C., Ingoglia, S., Liga, F., Coco, A. L., Cricchio, M. G. L., Musso, P., ... & Lim, H. J. (2016). Parenting dimensions and internalizing difficulties in Italian and US emerging adults: The intervening role of autonomy and relatedness. Journal of Child and Family Studies, 25(2), 419-431.

Jackson-Kersey, R., & Spray, C. (2016). The effect of perceived psychological need support on amotivation in physical education. European Physical Education Review, 22(1), 99-112.

Jang, H. R. (2019). Teachers' intrinsic vs. extrinsic instructional goals predict their classroom motivating styles. Learning and Instruction, 60, 286-300.

Jang, H., Reeve, J., & Deci, E. L. (2010). Engaging students in learning activities: It is not autonomy support or structure but autonomy support and structure. Journal of educational psychology, 102(3), 588.

Jang, H. R., Reeve, J., Cheon, S. H., & Song, Y. G. (2020). Dual processes to explain longitudinal gains in physical education students’ prosocial and antisocial behavior: Need satisfaction from autonomy support and need frustration from interpersonal control. Sport, Exercise, and Performance Psychology, 9(3), 471.

Jang, H., Reeve, J., Ryan, R. M., & Kim, A. (2009). Can self-determination theory explain what underlies the productive, satisfying learning experiences of collectivistically oriented Korean students?. Journal of educational Psychology, 101(3), 644.

Jang, H., Kim, E. J., & Reeve, J. (2012). Longitudinal test of self-determination theory's motivation mediation model in a naturally occurring classroom context. Journal of Educational psychology, 104(4), 1175.

Jang, H., Kim, E. J., & Reeve, J. (2016). Why students become more engaged or more disengaged during the semester: A self-determination theory dual-process model. Learning and Instruction, 43, 27-38.

Janssens, A., Van Den Noortgate, W., Goossens, L., Verschueren, K., Colpin, H., Claes, S., ... & Van Leeuwen, K. (2017). Adolescent externalizing behaviour, psychological control, and peer rejection: Transactional links and dopaminergic moderation. British journal of developmental psychology, 35(3), 420-438.

Jeno, L. M., Raaheim, A., Kristensen, S. M., Kristensen, K. D., Hole, T. N., Haugland, M. J., & Mæland, S. (2017). The relative effect of team-based learning on motivation and learning: a self-determination theory perspective. CBE—Life Sciences Education, 16(4), ar59.

Ji, X., Zheng, S., Cheng, C., Cheng, L., & Cronin, L. (2022). Development and psychometric evaluation of the Chinese version of the life skills scale for physical education. International Journal of Environmental Research and Public Health, 19(9), 5324.

Jiang, A. L., & Zhang, L. J. (2021). University teachers' teaching style and their students' agentic engagement in EFL learning in China: a self-determination theory and achievement goal theory integrated perspective. Frontiers in psychology, 12.

Jiang, Y. H., Yau, J., Bonner, P., & Chiang, L. (2011). The role of perceived parental autonomy support in academic achievement of Asian and Latin American adolescents.

Joussemet, M., Koestner, R., Lekes, N., & Landry, R. (2005). A longitudinal study of the relationship of maternal autonomy support to children's adjustment and achievement in school. Journal of personality, 73(5), 1215-1236.

Ju, S. G., Chen, X., Chen, L., Zhao, S., & Fegley, S. G. (2020). Relations of maternal power assertion and autonomy support with children’s adjustment in Korea. Journal of Family Psychology.

Jung, E., Hwang, W., Kim, S., Sin, H., Zhao, Z., Zhang, Y., & Park, J. H. (2020). Helicopter Parenting, Autonomy Support, and Student Wellbeing in the United States and South Korea. Journal of Child and Family Studies, 29(2), 358-373.

Jungert, T., & Koestner, R. (2015). Science adjustment, parental and teacher autonomy support and the cognitive orientation of science students. Educational Psychology, 35(3), 361-376.

Jungert, T., Piroddi, B., & Thornberg, R. (2016). Early adolescents' motivations to defend victims in school bullying and their perceptions of student–teacher relationships: A self-determination theory approach. Journal of adolescence, 53, 75-90.

Kalajas-Tilga, H., Koka, A., Hein, V., Tilga, H., & Raudsepp, L. (2020). Motivational processes in physical education and objectively measured physical activity among adolescents. Journal of Sport and Health Science, 9(5), 462-471.

Kaplan, H., & Assor, A. (2012). Enhancing autonomy-supportive I–Thou dialogue in schools: Conceptualization and socio-emotional effects of an intervention program. Social psychology of education, 15(2), 251-269.

Kaplan, H., & Madjar, N. (2015). Autonomous motivation and pro-environmental behaviours among Bedouin students in Israel: a self-determination theory perspective. Australian Journal of Environmental Education, 31(2), 223.

Kaplan, H., & Madjar, N. (2017). The motivational outcomes of psychological need support among pre-service teachers: Multicultural and self-determination theory perspectives. In Frontiers in Education (Vol. 2, p. 42). Frontiers.

Kaplan, H. (2018). Teachers’ autonomy support, autonomy suppression and conditional negative regard as predictors of optimal learning experience among high-achieving Bedouin students. Social Psychology of Education, 21(1), 223-255.

Kaplan, H. (2018). Teachers’ autonomy support, autonomy suppression and conditional negative regard as predictors of optimal learning experience among high-achieving Bedouin students. Social Psychology of Education, 21, 223-255

Karagiannidis, Y., Barkoukis, V., Gourgoulis, V., Kosta, G., & Antoniou, P. (2015). The role of motivation and metacognition on the development of cognitive and affective responses in physical education lessons: A self-determination approach. Motricidade, 11(1), 135-150.

Karbach, J., Gottschling, J., Spengler, M., Hegewald, K., & Spinath, F. M. (2013). Parental involvement and general cognitive ability as predictors of domain-specific academic achievement in early adolescence. Learning and Instruction, 23, 43-51.

Katz, I., Madjar, N., & Harari, A. (2015). Parental support and adolescent motivation for dieting: The self-determination theory perspective. The journal of Psychology, 149(5), 461-479.

Katz, I., Cohen, R., Green-Cohen, M., & Morsiano-davidpur, S. (2018). Parental support for adolescents' autonomy while making a first career decision. Learning and Individual Differences, 65, 12-19.

Katz, I., Lemish, D., Cohen, R., & Arden, A. (2019). When parents are inconsistent: Parenting style and adolescents' involvement in cyberbullying. Journal of adolescence, 74, 1-12.

Kenny, M. E., Walsh-Blair, L. Y., Blustein, D. L., Bempechat, J., & Seltzer, J. (2010). Achievement motivation among urban adolescents: Work hope, autonomy support, and achievement-related beliefs. Journal of Vocational Behavior, 77(2), 205-212.

Kiefer, S. M., & Pennington, S. (2017). ASSOCIATIONS OF TEACHER AUTONOMY SUPPORTAND STRUCTURE WITH YOUNG ADOLESCENTS’MOTIVATION, ENGAGEMENT, BELONGING, AND ACHIEVEMENT. Middle Grades Research Journal Issue: Volume 11# 1, 29.

Kikas, E., & Tang, X. (2019). Child-perceived teacher emotional support, its relations with teaching practices, and task persistence. European Journal of Psychology of Education, 34, 359-374.

Kındap-Tepe, Y., & Aktaş, V. (2019). The Mediating Role of Needs Satisfaction for Prosocial Behavior and Autonomy Support. Current Psychology, 1-13.

Kins, E., Beyers, W., Soenens, B., & Vansteenkiste, M. (2009). Patterns of home leaving and subjective well-being in emerging adulthood: The role of motivational processes and parental autonomy support. Developmental psychology, 45(5), 1416.

Knollmann, M., & Wild, E. (2007). Quality of parental support and students’ emotions during homework: Moderating effects of students’ motivational orientations. European journal of Psychology of Education, 22(1), 63.

Koçak, A., Mouratidis, A., Uçanok, Z., Selcuk, E., & Davies, P. T. (2020). Need Satisfaction as a Mediator of Associations between Interparental Relationship Dimensions and Autonomy Supportive Parenting: A Weekly Diary Study. Family Process.

Kocayörük, E., Altıntas, E., & İçbay, M. A. (2015). The perceived parental support, autonomous-self and well-being of adolescents: A cluster-analysis approach. Journal of Child and Family Studies, 24(6), 1819-1828.

Koka, A., Tilga, H., Hein, V., Kalajas-Tilga, H., & Raudsepp, L. (2021). A multidimensional approach to perceived teachers’ autonomy support and its relationship with intrinsic motivation of students in physical education. International Journal of Sport Psychology, 52(3), 266-286.

Koka, A., & Sildala, H. (2018). Gender differences in the relationships between perceived teachers’ controlling behaviors and amotivation in physical education. Journal of Teaching in Physical Education, 37(2), 197-208.

Koka, A., Tilga, H., Kalajas-Tilga, H., Hein, V., & Raudsepp, L. (2019). Perceived controlling behaviors of physical education teachers and objectively measured leisure-time physical activity in adolescents. International journal of environmental research and public health, 16(15), 2709.

Koka, A., Tilga, H., Kalajas-Tilga, H., Hein, V., & Raudsepp, L. (2020). Detrimental effect of perceived controlling behavior from physical education teachers on students’ leisure-time physical activity intentions and behavior: An application of the trans-contextual model. International Journal of Environmental Research and Public Health, 17(16), 5939.

Kouros, C. D., Pruitt, M. M., Ekas, N. V., Kiriaki, R., & Sunderland, M. (2017). Helicopter parenting, autonomy support, and college students’ mental health and well-being: The moderating role of sex and ethnicity. Journal of Child and Family Studies, 26(3), 939-949.

Kulakow, S. (2020). How autonomy support mediates the relationship between self-efficacy and approaches to learning. The Journal of Educational Research, 113(1), 13-25.

Kurdi, V., Archambault, I., Brière, F. N., & Turgeon, L. (2018). Need-supportive teaching practices and student-perceived need fulfillment in low socioeconomic status elementary schools: The moderating effect of anxiety and academic achievement. Learning and Individual Differences, 65, 218-229.

Lan, X., & Wang, W. (2020). Is early left-behind experience harmful to prosocial behavior of emerging adult? The role of parental autonomy support and mindfulness. Current Psychology, 1-14.

Lan, X., Zhang, L., & Radin, R. (2019). Shields for emotional well-being in Chinese adolescents who switch schools: the role of teacher autonomy support and grit. Frontiers in psychology, 10, 2384.

Lan, X., Ma, C., & Radin, R. (2019). Parental autonomy support and psychological well-being in tibetan and han emerging adults: a serial multiple mediation model. Frontiers in psychology, 10, 621.

Langdon, J., Webster, C., Hall, T., & Monsma, E. (2014). A self-determination theory perspective of student performance at the end of a volleyball unit in compulsory high school physical education. Sport Scientific & Practical Aspects, 11(1), 5-16. Retrieved from http://ez.library.latrobe.edu.au/login?url=http://search.ebscohost.com/login.aspx?direct=true&db=s3h&AN=103597686&site=ehost-live&scope=site

Langdon, J., Johnson, C., & Melton, B. (2017). Factors contributing to the uptake and maintenance of regular exercise behaviour in emerging adults. Health Education Journal, 76(2), 182-193.

Langdon, J. L., Schlote, R., Melton, B., & Tessier, D. (2017). Effectiveness of a need supportive teaching training program on the developmental change process of graduate teaching assistants' created motivational climate. Psychology of Sport and Exercise, 28, 11-23.

Larose, S. (2013). Trajectories of mentors’ perceived self-efficacy during an academic mentoring experience: What they look like and what are their personal and experimental correlates?. Mentoring & Tutoring: Partnership in Learning, 21(2), 150-174.

Lauermann, F., & Berger, J. L. (2021). Linking teacher self-efficacy and responsibility with teachers’ self-reported and student-reported motivating styles and student engagement. Learning and Instruction, 101441.

Laukkanen, A., Aunola, K., Korhonen, E., Barnett, L. M., & Sääkslahti, A. (2021). Construct validity and reliability of the physical activity parenting questionnaire for children (PAP-C). International Journal of Behavioral Nutrition and Physical Activity, 18(1), 1-12.

Laurin, J. C., & Joussemet, M. (2017). Parental autonomy-supportive practices and toddlers’ rule internalization: A prospective observational study. Motivation and Emotion, 41(5), 562-575.

Lavigne, G. L., Vallerand, R. J., & Miquelon, P. (2007). A motivational model of persistence in science education: A self-determination theory approach. European Journal of Psychology of Education, 22(3), 351.

Laxdal, A., Johannsson, E., & Giske, R. (2020). The role of perceived competence in determining teacher support in upper secondary school physical education. Physical Educator, 77(2), 384-403.

Lazarides, R., & Rubach, C. (2017). Instructional characteristics in mathematics classrooms: relationships to achievement goal orientation and student engagement. Mathematics Education Research Journal, 29(2), 201-217.

Lazarides, R., Rohowski, S., Ohlemann, S., & Ittel, A. (2016). The role of classroom characteristics for students’ motivation and career exploration. Educational Psychology, 36(5), 992-1008.

Lee, M., Cho, S., & Lee, S. M. (2019). A multilevel analysis of change in hatred of academic work during high school: focusing on the sociocultural background of Korea. Journal of Psychologists and Counsellors in Schools, 29(2), 166-176.

Lee, A. S., Standage, M., Hagger, M. S., & Chan, D. K. (2020). Predictors of in‐school and out‐of‐school sport injury prevention: A test of the trans‐contextual model. Scandinavian Journal of Medicine & Science in Sports.

Lee, A. S., Standage, M., Hagger, M. S., & Chan, D. K. (2021). Applying the trans‐contextual model to promote sport injury prevention behaviors among secondary school students. Scandinavian journal of medicine & science in sports, 31(9), 1840-1852.

Leflot, G., Onghena, P., & Colpin, H. (2010). Teacher–child interactions: relations with children's self‐concept in second grade. Infant and child development, 19(4), 385-405.

Legrain, P., Gillet, N., Gernigon, C., & Lafreniere, M.-A. (2015). Integration of Information and Communication Technology and Pupils' Motivation in a Physical Education Setting. Journal of Teaching in Physical Education, 34(3), 384-401.

Leisterer, S., & Gramlich, L. (2021). Having a Positive Relationship to Physical Activity: Basic Psychological Need Satisfaction and Age as Predictors for Students’ Enjoyment in Physical Education. Sports, 9(7), 90.

Lekes, N., Gingras, I., Philippe, F. L., Koestner, R., & Fang, J. (2010). Parental autonomy-support, intrinsic life goals, and well-being among adolescents in China and North America. Journal of youth and adolescence, 39(8), 858-869.

Leo, F. M., Pulido, J. J., Sánchez-Oliva, D., López-Gajardo, M. A., & Mouratidis, A. (2022). See the forest by looking at the trees: Physical education teachers’ interpersonal style profiles and students’ engagement. European Physical Education Review, 28(3), 720-738.

Leo, F. M., López-Gajardo, M. A., Rodríguez-González, P., Pulido, J. J., & Fernández-Río, J. (2023). How class cohesion and teachers’ relatedness supportive/thwarting style relate to students’ relatedness, motivation, and positive and negative outcomes in physical education. Psychology of Sport and Exercise, 65, 102360.

Leptokaridou, E. T., Vlachopoulos, S. P., & Papaioannou, A. G. (2016). Experimental longitudinal test of the influence of autonomy-supportive teaching on motivation for participation in elementary school physical education. Educational Psychology, 36(7), 1138-1159.

Levine, S. L., Milyavskaya, M., Powers, T. A., Holding, A. C., & Koestner, R. (2021). Autonomous motivation and support flourishes for individuals higher in collaborative personality factors: Agreeableness, assisted autonomy striving, and secure attachment. Journal of Personality.

Leyton-Román, M., Guíu-Carrera, M., Coto-Cañamero, A., & Jiménez-Castuera, R. (2020). Motivational variables to predict autotelic experience and enjoyment of students. Analysis in function of environment and sports practice. Sustainability, 12(6), 2352.

Leyton-Román, M., Núñez, J. L., & Jiménez-Castuera, R. (2020). The Importance of Supporting Student Autonomy in Physical Education Classes to Improve Intention to Be Physically Active. Sustainability, 12(10), 4251.

Leyton-Román, M., González-Vélez, J. J. L., Batista, M., & Jiménez-Castuera, R. (2020). Predictive Model for Amotivation and Discipline in Physical Education Students Based on Teaching–Learning Styles. Sustainability, 13(1), 187. doi:10.3390/su13010187

Leyva, D., Reese, E., Grolnick, W., & Price, C. (2009). Elaboration and autonomy support in low-income mothers' reminiscing: Links to children's autobiographical narratives. Journal of Cognition and Development, 9(4), 363-389.

Li, J., Deng, M., Wang, X., & Tang, Y. (2018). Teachers' and parents' autonomy support and psychological control perceived in junior-high school: Extending the dual-process model of self-determination theory. Learning and Individual Differences, 68, 20-29.

Li, C., Kee, Y. H., Kong, L. C., Zou, L., Ng, K. L., & Li, H. (2019). Autonomy-supportive teaching and basic psychological need satisfaction among school students: The role of mindfulness. International journal of environmental research and public health, 16(14), 2599.

Li, J., Yao, M., Liu, H., & Zhang, L. (2021). Influence of personality on work engagement and job satisfaction among young teachers: mediating role of teaching style. Current Psychology, 1-11.

Li, W., Gao, W., & Sha, J. (2020). Perceived Teacher Autonomy Support and School Engagement of Tibetan Students in Elementary and Middle Schools: Mediating Effect of Self-Efficacy and Academic Emotions. Frontiers in Psychology, 11, 50.

Lietaert, S., Roorda, D., Laevers, F., Verschueren, K., & De Fraine, B. (2015). The gender gap in student engagement: The role of teachers’ autonomy support, structure, and involvement. British Journal of Educational Psychology, 85(4), 498-518.

Liew, J., Kwok, O., Chang, Y. P., Chang, B. W., & Yeh, Y. C. (2014). Parental autonomy support predicts academic achievement through emotion-related self-regulation and adaptive skills in Chinese American adolescents. Asian American journal of psychology, 5(3), 214.

Lim, B. S. C., & Wang, C. K. J. (2009). Perceived autonomy support, behavioural regulations in physical education and physical activity intention. Psychology of Sport and Exercise, 10(1), 52-60. doi:10.1016/j.psychsport.2008.06.009

Lin, Y. Y. (2020). Support matters: Predictors of intrinsic motivation in older learners in Taiwan. Australian Journal of Adult Learning, 60(2), 190-212.

Litalien, D., & Guay, F. (2015). Dropout intentions in PhD studies: A comprehensive model based on interpersonal relationships and motivational resources. Contemporary Educational Psychology, 41, 218-231.

Liu, G., Zhang, S., Zhang, J., Lee, C., Wang, Y., & Brownell, M. (2013). Autonomous motivation and Chinese adolescents’ creative thinking: The moderating role of parental involvement. Creativity research journal, 25(4), 446-456.

Liu, J., Bartholomew, K., & Chung, P. K. (2017). Perceptions of teachers’ interpersonal styles and well-being and ill-being in secondary school physical education students: The role of need satisfaction and need frustration. School Mental Health, 9(4), 360-371.

Liu, S., Wang, M., & Fu, C. (2021). Maternal academic involvement and adolescents’ subjective well-being: The mediating role of adolescents’ academic adjustment in China. Children and Youth Services Review, 128, 106154.

Liu, H., Yao, M., Li, J., & Li, R. (2021). Multiple mediators in the relationship between perceived teacher autonomy support and student engagement in math and literacy learning. Educational Psychology, 41(2), 116-136.

Ljubin-Golub, T., Rijavec, M., & Olčar, D. (2020). Student flow and burnout: The role of teacher autonomy support and student autonomous motivation. Psychological Studies, 65(2), 145-156.

Lodewyk, K. R. (2019). Gender-Specific Associations Between Kinesiology Undergraduate Students’ Personality Traits and their Motivation in School Physical Education. International Journal of Kinesiology in Higher Education, 3(2), 35-46.

Lodewyk, K. R., & Pybus, C. M. (2013). Investigating factors in the retention of students in high school physical education. Journal of Teaching in Physical Education, 32(1), 61-77.

López-García, G. D., Carrasco-Poyatos, M., Burgueño, R., & Granero-Gallegos, A. (2022). Teaching style and academic engagement in pre-service teachers during the COVID-19 lockdown: Mediation of motivational climate. Frontiers in Psychology, 13.

Lozano-Jiménez, J. E., Huéscar, E., & Moreno-Murcia, J. A. (2021). From autonomy support and grit to satisfaction with life through self-determined motivation and group cohesion in higher education. Frontiers in Psychology, 11, 3734.

Lunkenheimer, E., Ram, N., Skowron, E. A., & Yin, P. (2017). Harsh parenting, child behavior problems, and the dynamic coupling of parents’ and children’s positive behaviors. Journal of Family Psychology, 31(6), 689.

Ma, C., Ma, Y., & Lan, X. (2020). A Structural Equation Model of Perceived Autonomy Support and Growth Mindset in Undergraduate Students: The Mediating Role of Sense of Coherence. Frontiers in Psychology, 11, 2055.

Mabbe, E., Soenens, B., Vansteenkiste, M., van der Kaap-Deeder, J., & Mouratidis, A. (2018). Day-to-day variation in autonomy-supportive and psychologically controlling parenting: The role of parents’ daily experiences of need satisfaction and need frustration. Parenting, 18(2), 86-109.

Madjar, N., Nave, A., & Hen, S. (2013). Are teachers’ psychological control, autonomy support and autonomy suppression associated with students’ goals?. Educational Studies, 39(1), 43-55.

Mageau, G. A., Ranger, F., Joussemet, M., Koestner, R., Moreau, E., & Forest, J. (2015). Validation of the Perceived Parental Autonomy Support Scale (P-PASS). Canadian Journal of Behavioural Science/Revue canadienne des sciences du comportement, 47(3), 251.

Mageau, G. A., Bureau, J. S., Ranger, F., Allen, M. P., & Soenens, B. (2016). The role of parental achievement goals in predicting autonomy-supportive and controlling parenting. Journal of Child and Family Studies, 25(5), 1702-1711.

Mageau, G. A., Sherman, A., Grusec, J. E., Koestner, R., & Bureau, J. S. (2017). Different ways of knowing a child and their relations to mother‐reported autonomy support. Social Development, 26(3), 630-644.

Maldonado, E., Zamarripa, J., Ruiz-Juan, F., Pacheco, R., & Delgado, M. (2019). Teacher Autonomy Support in Physical Education Classes as a Predictor of Motivation and Concentration in Mexican Students. Frontiers in Psychology, 10, 2834.

Mammadov, S., & Hertzog, N. B. (2021). Changes in students’ achievement goals in advanced learning environment: a multivariate multilevel model. Educational Psychology, 41(9), 1097-1116.

Manninen, M., Deng, Y., Hwang, Y., Waller, S., & Yli-Piipari, S. (2020). Psychological need-supportive instruction improves novel skill performance, intrinsic motivation, and enjoyment: a cluster-randomised study. International Journal of Sport and Exercise Psychology, 1-25.

Marbell, K. N., & Grolnick, W. S. (2013). Correlates of parental control and autonomy support in an interdependent culture: A look at Ghana. Motivation and Emotion, 37(1), 79-92.

Marbell‐Pierre, K. N., Grolnick, W. S., Stewart, A. L., & Raftery‐Helmer, J. N. (2019). Parental autonomy support in two cultures: The moderating effects of adolescents’ self‐construals. Child Development, 90(3), 825-845.

Martinek, D., Zumbach, J., & Carmignola, M. (2020). The impact of perceived autonomy support and autonomy orientation on orientations towards teaching and self-regulation at university. International Journal of Educational Research, 102, 101574.

Martinent, G., Ferrand, C., Humblot, E., Bauvineau, A., & Noisiez, M. (2019). Role of need-supportive family behaviours on purpose in life and depressive feelings of French older people: A self-determination theory perspective. Applied Sciences, 9(1), 115.

Marušić, I., Jugović, I., & Lončarić, D. (2017). Approaches to learning of first-year and fifth-year student teachers: are there any differences?. European journal of teacher education, 40(1), 62-75.

Matos, L., Reeve, J., Herrera, D., & Claux, M. (2018). Students' agentic engagement predicts longitudinal increases in perceived autonomy-supportive teaching: The squeaky wheel gets the grease. The Journal of Experimental Education, 86(4), 579-596.

Matte-Gagné, C., & Bernier, A. (2011). Prospective relations between maternal autonomy support and child executive functioning: Investigating the mediating role of child language ability. Journal of experimental child psychology, 110(4), 611-625.

Matte‐Gagné, C., Bernier, A., & Gagné, C. (2013). Stability of maternal autonomy support between infancy and preschool age. Social Development, 22(3), 427-443.

Maulana, R., Helms-Lorenz, M., Irnidayanti, Y., & van de Grift, W. (2016). Autonomous motivation in the Indonesian classroom: Relationship with teacher support through the lens of self-determination theory. The Asia-Pacific Education Researcher, 25(3), 441-451.

Mauras, C. P., Grolnick, W. S., & Friendly, R. W. (2013). Time for “The talk”... Now what? Autonomy support and structure in mother-daughter conversations about sex. The Journal of Early Adolescence, 33(4), 458-489

Mavropoulou, A., Barkoukis, V., Douka, S., Alexandris, K., & Hatzimanouil, D. (2019). The role of autonomy supportive activities on students’ motivation and beliefs toward out-of-school activities. The Journal of Educational Research, 112(2), 223-233.

Mazlum, F., Cheraghi, F., & Dasta, M. (2015). English teachers' self-efficacy beliefs and students learning approaches. International Journal of Educational Psychology: IJEP, 4(3), 305-328.

McCurdy, A. L. (2021). Relations Between Parental Autonomy Support and Child Anxiety Symptoms Across Elementary School in Two-Parent Families (Doctoral dissertation, The University of North Carolina at Greensboro).

McDavid, L., Cox, A. E., & Amorose, A. J. (2012). The relative roles of physical education teachers and parents in adolescents' leisure-time physical activity motivation and behavior. Psychology of Sport and Exercise, 13(2), 99-107. doi:10.1016/j.psychsport.2011.10.011

McDavid, L., McDonough, M. H., Blankenship, B. T., & LeBreton, J. M. (2017). A test of basic psychological needs theory in a physical-activity-based program for underserved youth. Journal of Sport and Exercise Psychology, 39(1), 29-42.

McEown, K., & Sugita-McEown, M. (2019). Individual, parental and teacher support factors of self-regulation in Japanese students. Innovation in Language Learning and Teaching, 13(4), 389-401.

McRae, A. (2012). Teacher competence support for reading in middle school. University of Maryland, College Park.

Meeus, A., Eggermont, S., & Beullens, K. (2019). Constantly connected: The role of parental mediation styles and self-regulation in pre-and early adolescents’ problematic mobile device use. Human Communication Research, 45(2), 119-174

García Mendoza, M. D. C., Sánchez Queija, I., & Parra Jiménez, Á. (2019). The role of parents in emerging adults’ psychological well‐being: A person‐oriented approach. Family process, 58(4), 954-971.

Meng, H. Y., & Keng, J. W. C. (2015). The effectiveness of an Autonomy-Supportive Teaching Structure in Physical Education.[Eficacia de la estructura de enseñanza con soporte de autonomía en educación física]. RICYDE. Revista Internacional de Ciencias del Deporte. doi: 10.5232/ricyde, 12(43), 5-28.

Merki, K. M., & Oerke, B. (2017). Long-term effects of the implementation of state-wide exit exams: a multilevel regression analysis of mediation effects of teaching practices on students’ motivational orientations. Educational Assessment, Evaluation and Accountability, 29(1), 23-54.

Meuwissen, A. S., & Carlson, S. M. (2018). The role of father parenting in children’s school readiness: A longitudinal follow-up. Journal of Family Psychology, 32(5), 588.

Michou, A., Altan, S., Mouratidis, A., Reeve, J., & Malmberg, L. E. (2021). Week-to-week interplay between teachers’ motivating style and students’ engagement. The Journal of Experimental Education, 1-20.

Miketinas, D., Cater, M., Bailey, A., Craft, B., & Tuuri, G. (2016). Exploratory and confirmatory factor analysis of the Adolescent Motivation to Cook Questionnaire: A Self-Determination Theory instrument. Appetite, 105, 527-533.

Moè, A., Katz, I., & Alesi, M. (2018). Scaffolding for motivation by parents, and child homework motivations and emotions: Effects of a training programme. British Journal of Educational Psychology, 88(2), 323-344.

Montero-Carretero, C., Barbado, D., & Cervelló, E. (2020). Predicting bullying through motivation and teaching styles in physical education. International journal of environmental research and public health, 17(1), 87.

Montero-Carretero, C., & Cervelló, E. (2019). Teaching Styles in Physical Education: A New Approach to Predicting Resilience and Bullying. International Journal of Environmental Research and Public Health, 17(1), 76. doi:10.3390/ijerph17010076

Moreno-Murcia, J., Huéscar Hernández, E., & Ruiz, L. (2018). Perceptions of Controlling Teaching Behaviors and the Effects on the Motivation and Behavior of High School Physical Education Students. International Journal of Environmental Research and Public Health, 15(10), 2288. doi:10.3390/ijerph15102288

Moreno-Murcia, J. A., & Sánchez-Latorre, F. (2015). The effects of autonomy support in physical education classes [Efectos del soporte de autonomía en clases de educación física]. RICYDE. Revista Internacional de Ciencias del Deporte. doi: 10.5232/ricyde, 12(43), 79-89.

Moreno-Murcia, J. A., Ruiz, M., & Vera, J. A. (2015). Prediction of autonomy support, psychological mediators and academic motivation on basic competences in adolescent students. Revista de Psicodidáctica, 20(2), 359-376.

Moreno-Murcia, J. A., Huéscar Hernández, E., Cid, L., Monteiro, D., Rodrigues, F., Teixeira, D., ... & Guedea Delgado, J. C. (2020). Assessing the Relationship between Autonomy Support and Student Group Cohesion across Ibero-American Countries. International Journal of Environmental Research and Public Health, 17(11), 3981.

Morrison, S. A., Dashiff, C. J., & Vance, D. E. (2013). Role of parental autonomy support on self-determination in influencing diet and exercise motivation in older adolescents. Nursing: Research and Reviews, 3, 77-85.

Mouratidis, A. A., Vansteenkiste, M., Sideridis, G., & Lens, W. (2011). Vitality and interest–enjoyment as a function of class-to-class variation in need-supportive teaching and pupils' autonomous motivation. Journal of Educational Psychology, 103(2), 353.

Mouratidis, A., Michou, A., Aelterman, N., Haerens, L., & Vansteenkiste, M. (2018). Begin-of-school-year perceived autonomy-support and structure as predictors of end-of-school-year study efforts and procrastination: the mediating role of autonomous and controlled motivation. Educational Psychology, 38(4), 435-450.

Mouratidis, A., Michou, A., Telli, S., Maulana, R., & Helms‐Lorenz, M. (2022). No aspect of structure should be left behind in relation to student autonomous motivation. British Journal of Educational Psychology, 92(3), 1086-1108.

Müftüler, M., & İnce, M. L. (2015). Use of trans-contextual model-based physical activity course in developing leisure-time physical activity behavior of university students. Perceptual and motor skills, 121(1), 31-55.

Murcia, J. A. M., Rojas, N. P., & Coll, D. G. C. (2008). Influencia del apoyo a la autonomía, las metas sociales y la relación con los demás sobre la desmotivación en educación física. Psicothema, 20(4), 636-641.

Myrold, R. L., & Ullrich-French, S. (2017). Expectancy-value theory outcomes of a school-based bicycling programme. International Journal of Sport and Exercise Psychology, 15(2), 207-220. doi:10.1080/1612197X.2015.1079923

Nadler, D. R., & Komarraju, M. (2016). Negating stereotype threat: Autonomy support and academic identification boost performance of African American college students. Journal of College Student Development, 57(6), 667-679.

Nerona, R. R. (2021). Parenting, Major Choice Motivation, and Academic Major Satisfaction Among Filipino College Students: A Self-Determination Theory Perspective. Journal of Career Assessment, 29(2), 205-220.

Neufeld, A., & Malin, G. (2020). How medical students’ perceptions of instructor autonomy-support mediate their motivation and psychological well-being. Medical Teacher, 1-7.

Ng, B. (2016). Towards Lifelong Learning: Identifying Learner Profiles on Procrastination and Self-Regulation. New Waves-Educational Research and Development Journal, 19(1), 41-54.

Fei‐Yin Ng, F., Kenney‐Benson, G. A., & Pomerantz, E. M. (2004). Children's achievement moderates the effects of mothers' use of control and autonomy support. Child Development, 75(3), 764-780.

Ng, B. L., Liu, W. C., & Wang, J. C. (2016). Student motivation and learning in mathematics and science: A cluster analysis. International Journal of Science and Mathematics Education, 14(7), 1359-1376.

Niemiec, C. P., Lynch, M. F., Vansteenkiste, M., Bernstein, J., Deci, E. L., & Ryan, R. M. (2006). The antecedents and consequences of autonomous self-regulation for college: A self-determination theory perspective on socialization. Journal of adolescence, 29(5), 761-775.

Nishimura, T., Bradshaw, E. L., Deci, E. L., & Ryan, R. M. (2021). Satisfaction of basic psychological needs in an interdependence model of fathers’ own aspirations and those of their adolescent children. Social Development, 30(1), 293-310.

Ntoumanis, N. (2005). A prospective study of participation in optional school physical education using a self-determination theory framework. Journal of Educational Psychology, 97(3), 444-453. doi:10.1037/0022-0663.97.3.458

Núñez, J. L., & León, J. (2016). The Mediating Effect of Intrinsic Motivation to Learn on the Relationship between Student´ s Autonomy Support and Vitality and Deep Learning. The Spanish journal of psychology, 19.

Núñez, J. L., León, J., Grijalvo, F., & Martín-Albo Lucas, J. (2012). Measuring autonomy support in university students: the Spanish version of the learning climate questionnaire. Spanish Journal of Psychology.

Olivier, E., Galand, B., Hospel, V., & Dellisse, S. (2020). Understanding behavioural engagement and achievement: The roles of teaching practices and student sense of competence and task value. British Journal of Educational Psychology.

Ommundsen, Y., & Kvalo, S. E. (2007). Autonomy-Mastery, Supportive or Performance Focused? Different teacher behaviours and pupils' outcomes in physical education. Scandinavian Journal of Educational Research, 51(4), 385-413. doi:10.1080/00313830701485551

O'Reilly, E. N. (2014). Correlations among perceived autonomy support, intrinsic motivation, and learning outcomes in an intensive foreign language program. Theory and Practice in Language Studies, 4(7), 1313.

Oriol-Granado, X., Mendoza-Lira, M., Covarrubias-Apablaza, C. G., & Molina-López, V. M. (2017). Positive emotions, autonomy support and academic performance of university students: The mediating role of academic engagement and self-efficacy. Revista de Psicodidáctica (English Ed.), 22(1), 45-53.

O'Sullivan, R. H., Chen, Y. C., & Fish, M. C. (2014). Parental Mathematics Homework Involvement of Low-Income Families with Middle School Students. School Community Journal, 24(2), 165-188.

Overall, N. C., Deane, K. L., & Peterson, E. R. (2011). Promoting doctoral students' research self-efficacy: Combining academic guidance with autonomy support. Higher Education Research & Development, 30(6), 791-805.

Painter, J. (2011). Autonomy, competence, and intrinsic motivation in science education: A self-determination theory perspective.

Parr, A., Gladstone, J., Rosenzweig, E., & Wang, M. T. (2021). Why do I teach? A mixed-methods study of in-service teachers’ motivations, autonomy-supportive instruction, and emotions. Teaching and Teacher Education, 98, 103228.

Parrisius, C., Gaspard, H., Zitzmann, S., Trautwein, U., & Nagengast, B. (2021). The “situative nature” of competence and value beliefs and the predictive power of autonomy support: A multilevel investigation of repeated observations. Journal of Educational Psychology.

Pasi, H., Lintunen, T., Leskinen, E., & Hagger, M. S. (2021). Predicting school students’ physical activity intentions in leisure-time and school recess contexts: Testing an integrated model based on self-determination theory and theory of planned behavior. PloS one, 16(3), e0249019.

Patall, E. A., Pituch, K. A., Steingut, R. R., Vasquez, A. C., Yates, N., & Kennedy, A. A. (2019). Agency and high school science students' motivation, engagement, and classroom support experiences. Journal of Applied Developmental Psychology, 62, 77-92.

Patall, E. A., Steingut, R. R., Vasquez, A. C., Trimble, S. S., Pituch, K. A., & Freeman, J. L. (2018). Daily autonomy supporting or thwarting and students’ motivation and engagement in the high school science classroom. Journal of Educational Psychology, 110(2), 269.

Patall, E. A., Steingut, R. R., Freeman, J. L., Pituch, K. A., & Vasquez, A. C. (2018). Gender disparities in students’ motivational experiences in high school science classrooms. Science Education, 102(5), 951-977.

Pedersen, D. E. (2017). Parental autonomy support and college student academic outcomes. Journal of Child and Family Studies, 26(9), 2589-2601.

Peng, S., Zhou, B., Wang, X., Zhang, H., & Hu, X. (2020). Does high teacher autonomy support reduce smartphone use disorder in Chinese adolescents? A moderated mediation model. Addictive Behaviors, 105, 106319.

Pesch, K. M., Larson, L. M., & Surapaneni, S. (2016). Parental autonomy support and career well-being: Mediating effects of perceived academic competence and volitional autonomy. Journal of Career Assessment, 24(3), 497-512.

Polet, J., Lintunen, T., Schneider, J., & Hagger, M. S. (2020). Predicting change in middle school students’ leisure‐time physical activity participation: A prospective test of the trans‐contextual model. Journal of Applied Social Psychology, 50(9), 512-523.

Puente-Díaz, R., & Cavazos-Arroyo, J. (2017). Creative self-efficacy: The influence of affective states and social persuasion as antecedents and imagination and divergent thinking as consequences. Creativity Research Journal, 29(3), 304-312.

Qin, X., Kaufman, T., Laninga-Wijnen, L., Ren, P., Zhang, Y., & Veenstra, R. (2021). The Impact of Academic Achievement and Parental Practices on Depressive Symptom Trajectories Among Chinese Adolescents. Research on Child and Adolescent Psychopathology, 1-13.

Quested, E., & Duda, J. L. (2010). Exploring the social-environmental determinants of well-and ill-being in dancers: A test of basic needs theory. Journal of Sport and Exercise Psychology, 32(1), 39-60.

Raftery-Helmer, J. N., & Grolnick, W. S. (2016). Children’s coping with academic failure: Relations with contextual and motivational resources supporting competence. The Journal of Early Adolescence, 36(8), 1017-1041.

Rahnama, F. K., & Zafarghandi, A. M. (2013). Teachers’ Instructional Behaviors and Students’ Self-Determination. International Journal of Applied Linguistics and English Literature, 2(3), 100-111.

Ratelle, C. F., Larose, S., Guay, F., & Senécal, C. (2005). Perceptions of parental involvement and support as predictors of college students' persistence in a science curriculum. Journal of family psychology, 19(2), 286.

Ratelle, C. F., Duchesne, S., & Guay, F. (2017). Predicting school adjustment from multiple perspectives on parental behaviors. Journal of adolescence, 54, 60-72.

Ratelle, C. F., Morin, A. J., Guay, F., & Duchesne, S. (2018). Sources of evaluation of parental behaviors as predictors of achievement outcomes. Motivation and Emotion, 42(4), 513-526.

Ratelle, C. F., Duchesne, S., Litalien, D., & Plamondon, A. (2020). The role of mothers in supporting adaptation in school: A psychological needs perspective. Journal of Educational Psychology.

Reeve, J., & Tseng, C. M. (2011). Cortisol reactivity to a teacher’s motivating style: The biology of being controlled versus supporting autonomy. Motivation and Emotion, 35(1), 63-74.

Reeve, J. (2013). How students create motivationally supportive learning environments for themselves: The concept of agentic engagement. Journal of educational psychology, 105(3), 579.

Reeve, J., Cheon, S. H., & Yu, T. H. (2020). An autonomy-supportive intervention to develop students’ resilience by boosting agentic engagement. International Journal of Behavioral Development, 0165025420911103.

Robichaud, J. M., Normandin, A. L., & Mageau, G. A. (2021). The socializing role of the problem-constraint link: A multimethod investigation. Journal of Applied Developmental Psychology, 73, 101260.

Rocchi, M., Pelletier, L., Cheung, S., Baxter, D., & Beaudry, S. (2017). Assessing need-supportive and need-thwarting interpersonal behaviours: The Interpersonal Behaviours Questionnaire (IBQ). Personality and Individual Differences, 104, 423–433. doi:10.1016/j.paid.2016.08.034

Rockafellow, B. D. (2006). The contribution of self-determination theory to an understanding of psychological distress among young adults: Mediation of practical involvement and autonomy support by autonomy, controlled, and impersonal orientations.

Rodríguez-Meirinhos, A., Antolín-Suárez, L., & Oliva, A. (2021). Psychological needs in parents and clinically referred adolescents: An integrative model via parenting and parental mindfulness. Journal of Family Psychology.

Roth, G. (2008). Perceived parental conditional regard and autonomy support as predictors of young adults' self‐versus other‐oriented prosocial tendencies. Journal of Personality, 76(3), 513-534.

Roth, G., Assor, A., Niemiec, C. P., Ryan, R. M., & Deci, E. L. (2009). The emotional and academic consequences of parental conditional regard: Comparing conditional positive regard, conditional negative regard, and autonomy support as parenting practices. Developmental psychology, 45(4), 1119.

Roth, G., & Assor, A. (2012). The costs of parental pressure to express emotions: Conditional regard and autonomy support as predictors of emotion regulation and intimacy. Journal of adolescence, 35(4), 799-808.

Roth, G., Kanat‐Maymon, Y., & Assor, A. (2016). The role of unconditional parental regard in autonomy‐supportive parenting. Journal of personality, 84(6), 716-725.

Roth, G., Assor, A., Kanat-Maymon, Y., & Kaplan, H. (2007). Autonomous motivation for teaching: how self-determined teaching may lead to self-determined learning. Journal of educational psychology, 99(4), 761.

Roth, G., Kanat‐Maymon, Y., & Bibi, U. (2011). Prevention of school bullying: The important role of autonomy‐supportive teaching and internalization of pro‐social values. British Journal of Educational Psychology, 81(4), 654-666.

Rutten, C., Boen, F., & Seghers, J. (2013). The relation between environmental factors and pedometer-determined physical activity in children: the mediating role of autonomous motivation. Pediatric exercise science, 25(2), 273-287.

Ruzek, E. A., & Schenke, K. (2019). The tenuous link between classroom perceptions and motivation: A within-person longitudinal study. Journal of Educational Psychology, 111(5), 903.

Sánchez-Oliva, D., Pulido-González, J. J., Leo, F. M., González-Ponce, I., & García-Calvo, T. (2017). Effects of an intervention with teachers in the physical education context: A Self-Determination Theory approach. PloS one, 12(12), e0189986.

Sánchez-Oliva, D., Kinnafick, F. E., Smith, N., & Stenling, A. (2018). Assessing perceived need support and need satisfaction in physical education: adaptation and validation with English students. Measurement in Physical Education and Exercise Science, 22(4), 332-342.

Schuitema, J., Peetsma, T., & van der Veen, I. (2016). Longitudinal relations between perceived autonomy and social support from teachers and students' self-regulated learning and achievement. Learning and Individual Differences, 49, 32-45.

Sevil, J., García-González, L., Abós, Á., Generelo Lanaspa, E., & Aibar Solana, A. (2018). Which school community agents influence adolescents’ motivational outcomes and physical activity? Are more autonomy-supportive relationships necessarily better?. International journal of environmental research and public health, 15(9), 1875.

Sheldon, K. M., & Krieger, L. S. (2007). Understanding the negative effects of legal education on law students: A longitudinal test of self-determination theory. Personality and Social Psychology Bulletin, 33(6), 883-897.

Sheldon, K. M., Abad, N., & Omoile, J. (2009). Testing self-determination theory via Nigerian and Indian adolescents. International Journal of Behavioral Development, 33(5), 451-459.

Shen, B. (2010). How can perceived autonomy support influence enrollment in elective physical education? A prospective study. Research Quarterly for Exercise and Sport, 81(4), 456-465. doi:10.1080/02701367.2010.10599706

Shen, B., McCaughtry, N., Martin, J., & Fahlman, M. (2009). Effects of teacher autonomy support and students' autonomous motivation on learning in physical education. Research Quarterly for Exercise and Sport, 80(1), 44-53. doi:10.1080/02701367.2009.10599528

Shen, B., McCaughtry, N., Martin, J., Garn, A., Kulik, N., & Fahlman, M. (2015). The relationship between teacher burnout and student motivation. British Journal of Educational Psychology, 85(4), 519-532.

Shih, S. S. (2008). The relation of self-determination and achievement goals to Taiwanese eighth graders' behavioral and emotional engagement in schoolwork. The Elementary School Journal, 108(4), 313-334.

Shih, S. S. (2009). An examination of factors related to Taiwanese adolescents' reports of avoidance strategies. The Journal of Educational Research, 102(5), 377-388.

Shih, S. S. (2013). Autonomy support versus psychological control, perfectionism, and Taiwanese adolescents’ achievement goals. The Journal of Educational Research, 106(4), 269-279.

Shih, S. S. (2013). The effects of autonomy support versus psychological control and work engagement versus academic burnout on adolescents’ use of avoidance strategies. School Psychology International, 34(3), 330-347.

Shih, S. S. (2015). An investigation into academic burnout among Taiwanese adolescents from the self-determination theory perspective. Social Psychology of Education, 18(1), 201-219.

Sicilia, Á., Sáenz‐Alvarez, P., González‐Cutre, D., & Ferriz, R. (2015). Analysing the influence of autonomous and controlling social factors within the theory of planned behaviour. Australian Psychologist, 50(1), 70-79.

Sicilia, A., Águila, C., Posse, M., & Alcaraz-Ibáñez, M. (2020). Parents’ and Peers’ Autonomy Support and Exercise Intention for Adolescents: Integrating Social Factors from the Self-Determination Theory and the Theory of Planned Behaviour. International Journal of Environmental Research and Public Health, 17(15), 5365.

Sierens, E., Vansteenkiste, M., Goossens, L., Soenens, B., & Dochy, F. (2009). The synergistic relationship of perceived autonomy support and structure in the prediction of self‐regulated learning. British Journal of Educational Psychology, 79(1), 57-68.

Simões, F., & Calheiros, M. M. (2021). Multiple autonomy support attunement connections with perceived competence in learning and school grades among rural adolescents. Current Psychology, 1-14.

Simões, F. A. M., de Amorim Calheiros, M. M., e Silva, M. M. A., Sousa, Á. S. T., & da Silva, O. D. L. (2018). Total and attuned multiple autonomy support and the social development of early adolescents. Journal of Child and Family Studies, 27(2), 374-386.

Simon, P. D. (2021). Parent autonomy support as moderator: Testing the expanded perfectionism social disconnection model. Personality and Individual Differences, 168, 110401.

Simon, P. D., & Salanga, M. G. C. (2021). Validation of the Five‐item Learning Climate Questionnaire as a measure of teacher autonomy support in the classroom. Psychology in the Schools.

Simonton, K. L., Solmon, M. A., & Garn, A. C. (2021). Exploring perceived autonomy support and emotions in university tennis courses. International Journal of Sport and Exercise Psychology, 19(1), 134-148.

Sirois, M. S., & Bernier, A. (2018). Mother–child relationships and children’s psychosocial functioning: The specific roles of attachment security and maternal behavior. Parenting, 18(3), 172-189.

Smits, I., Soenens, B., Vansteenkiste, M., Luyckx, K., & Goossens, L. (2010). Why do adolescents gather information or stick to parental norms? Examining autonomous and controlled motives behind adolescents’ identity style. Journal of Youth and Adolescence, 39(11), 1343-1356.

Soenens, B., & Vansteenkiste, M. (2005). Antecedents and outcomes of self-determination in 3 life domains: The role of parents' and teachers' autonomy support. Journal of youth and adolescence, 34(6), 589-604.

Soenens, B., Vansteenkiste, M., Lens, W., Luyckx, K., Goossens, L., Beyers, W., & Ryan, R. M. (2007). Conceptualizing parental autonomy support: Adolescent perceptions of promotion of independence versus promotion of volitional functioning. Developmental psychology, 43(3), 633.

Soenens, B., Vansteenkiste, M., & Sierens, E. (2009). How are parental psychological control and autonomy‐support related? A cluster‐analytic approach. Journal of Marriage and Family, 71(1), 187-202.

Soenens, B., Park, S. Y., Mabbe, E., Vansteenkiste, M., Chen, B., Van Petegem, S., & Brenning, K. (2018). The moderating role of vertical collectivism in South-Korean adolescents’ perceptions of and responses to autonomy-supportive and controlling parenting. Frontiers in psychology, 9, 1080.

Ling, J., Soos, I., Dizmatsek, I., Ojelabi, A., Simonek, J., Iulianna, B. B., ... & Hamar, P. (2019). Perceived autonomy support and motivation in young people: A comparative investigation of physical education and leisure-time in four countries. Europe's Journal of Psychology, 15(3).

Sorariutta, A., & Silvén, M. (2018). Maternal cognitive guidance and early education and care as precursors of mathematical development at preschool age and in ninth grade. Infant and Child Development, 27(2), e2069.

Sparks, C., Dimmock, J., Lonsdale, C., & Jackson, B. (2016). Modeling indicators and outcomes of students’ perceived teacher relatedness support in high school physical education. Psychology of Sport and Exercise, 26, 71-82.

Sproule, J., Martindale, R., Wang, J., Allison, P., Nash, C., & Gray, S. (2013). Investigating the experience of outdoor and adventurous project work in an educational setting using a self-determination framework. European Physical Education Review, 19(3), 315-328.

Standage, M., & Gillison, F. (2007). Students' motivational responses toward school physical education and their relationship to general self-esteem and health-related quality of life. Psychology of Sport and Exercise, 8(5), 704-721. doi:10.1016/j.psychsport.2006.12.007

Standage, M., Duda, J. L., & Ntoumanis, N. (2006). Students' Motivational Processes and Their Relationship to Teacher Ratings in School Physical Education: A Self-Determination Theory Approach. Research Quarterly for Exercise & Sport, 77(1), 100-110.

Steele, J. P., & Fullagar, C. J. (2009). Facilitators and outcomes of student engagement in a college setting. The Journal of psychology, 143(1), 5-27.

Stiller, J. D., & Ryan, R. M. (1992). Teachers, Parents, and Student Motivation: The Effects of Involvement and Autonomy Support.

Summers, J. J., Bergin, D. A., & Cole, J. S. (2009). Examining the relationships among collaborative learning, autonomy support, and student incivility in undergraduate classrooms. Learning and Individual Differences, 19(2), 293-298.

Su-Russell, C., & Russell, L. T. (2021). Maternal Autonomy Support and Children’s Social Competencies, Academic Skills, and Persistence: Social Determinants and Mediation. Journal of Child and Family Studies, 30(3), 757-770.

Suviste, R., Kiuru, N., Palu, A., & Kikas, E. (2014). Classroom management practices and their associations with children’s mathematics skills in two cultural groups. Educational Psychology, 36(2), 216–235. doi:10.1080/01443410.2014.993932

Tan, D., Diatta-Holgate, H. A., & Levesque-Bristol, C. (2021). Perceived autonomy supportive and culturally responsive environments contribute to international students’ participation and willingness to communicate. Current Psychology, 1-20.

Tan, S. A., Nainee, S., & Tan, C. S. (2021). The mediating role of reciprocal filial piety in the relationship between parental autonomy support and life satisfaction among adolescents in Malaysia. Current Psychology, 40(2), 804-812.

Taylor, I. M., & Lonsdale, C. (2010). Cultural differences in the relationships among autonomy support, psychological need satisfaction, subjective vitality, and effort in British and Chinese physical education. Journal of Sport and Exercise Psychology, 32(5), 655-673.

Taylor, I. M., & Ntoumanis, N. (2007). Teacher motivational strategies and student self-determination in physical education. Journal of educational psychology, 99(4), 747.

Taylor, G., Lekes, N., Gagnon, H., Kwan, L., & Koestner, R. (2012). Need satisfaction, work–school interference and school dropout: An application of self‐determination theory. British Journal of Educational Psychology, 82(4), 622-646.

Temple, A. C. (2012). A model of student engagement and academic achievement: The role of teacher-student relationships and teacher expectations.

Tessier, D., Sarrazin, P., & Ntoumanis, N. (2008). The effects of an experimental programme to support students’ autonomy on the overt behaviours of physical education teachers. European Journal of Psychology of Education, 23(3), 239-253.

Teuber, Z., Tang, X., Sielemann, L., Otterpohl, N., & Wild, E. (2022). Autonomy-related Parenting Profiles and their Effects on Adolescents’ Academic and Psychological Development: A Longitudinal Person-oriented Analysis. Journal of Youth and Adolescence, 51(7), 1333-1353.

Thomas, A. E., & Mueller, F. H. (2017). A magic dwells in each beginning? Contextual effects of autonomy support on students’ intrinsic motivation in unfamiliar situations. Social Psychology of Education, 20(4), 791-805.

Tiede, M. A. (2020). The Impact of Parental Meta-Emotion Philosophy and Helicopter Parenting on Millennials’ Emotion Regulation: A Gendered Analysis. University of Rochester.

Tilga, H., Hein, V., & Koka, A. (2017). Measuring the perception of the teachers’ autonomy-supportive behavior in physical education: Development and initial validation of a multi-dimensional instrument. Measurement in Physical Education and Exercise Science, 21(4), 244-255.

Tilga, H., Hein, V., Koka, A., & Hagger, M. S. (2020). How physical education teachers’ interpersonal behaviour is related to students’ health-related quality of life. Scandinavian Journal of Educational Research, 64(5), 661-676.

Tilga, H., Kalajas-Tilga, H., Hein, V., Raudsepp, L., & Koka, A. (2021). Effects of a web-based autonomy-supportive intervention on physical education teacher outcomes. Education Sciences, 11(7), 316.

Tilga, H., Hein, V., Koka, A., Hamilton, K., & Hagger, M. S. (2019). The role of teachers’ controlling behaviour in physical education on adolescents’ health-related quality of life: test of a conditional process model*. Educational Psychology, 1–19. doi:10.1080/01443410.2018.1546830

Tilga, H., Kalajas-Tilga, H., Hein, V., Raudsepp, L., & Koka, A. (2020). How does perceived autonomy-supportive and controlling behaviour in physical education relate to adolescents’ leisure-time physical activity participation?. Kinesiology, 52(2), 265-272.

Tóth-Király, I., Bőthe, B., Jánvári, M., Rigó, A., & Orosz, G. (2019). Longitudinal trajectories of passion and their individual and social determinants: A latent growth modeling approach. Journal of Happiness Studies, 20(8), 2431-2444.

Trigueros, R., Aguilar-Parra, J. M., Cangas, A. J., López-Liria, R., & Álvarez, J. F. (2019). Influence of physical education teachers on motivation, embarrassment and the intention of being physically active during adolescence. International journal of environmental research and public health, 16(13), 2295.

Trigueros, R., Mínguez, L. A., González-Bernal, J. J., Aguilar-Parra, J. M., Soto-Cámara, R., Álvarez, J. F., & Rocamora, P. (2020). Physical Education Classes as a Precursor to the Mediterranean Diet and the Practice of Physical Activity. Nutrients, 12(1), 239.

Trigueros, R., González-Bernal, S., González-Bernal, J. J., & Aguilar-Parra, J. M. (2021). Healthy and balanced nutrition for children through physical education classes. Life, 11(7), 678.

Trigueros, R., Mínguez, L. A., González-Bernal, J. J., Jahouh, M., Soto-Camara, R., & Aguilar-Parra, J. M. (2019). Influence of Teaching Style on Physical Education Adolescents’ Motivation and Health-Related Lifestyle. Nutrients, 11(11), 2594. doi:10.3390/nu11112594

Ramos, R. T., & Gómez, N. N. (2019). La influencia del docente sobre la motivación, las estrategias de aprendizaje, pensamiento crítico de los estudiantes y rendimiento académico en el área de Educación Física. Psychology, Society & Education, 11(1), 137-150

Trigueros, R., Aguilar-Parra, J. M., Álvarez, J. F., & Cangas, A. J. (2019). Adaptation and Validation of the Mind-Wandering Questionnaire (MWQ) in Physical Education Classes and Analysis of Its Role as Mediator between Teacher and Anxiety. Sustainability, 11(18), 5081. doi:10.3390/su11185081

Trigueros, R., Cangas, A. J., Aguilar-Parra, J. M., Álvarez, J. F., & García-Más, A. (2019). No More Bricks in the Wall: Adopting Healthy Lifestyles through Physical Education Classes. International Journal of Environmental Research and Public Health, 16(23), 4860. doi:10.3390/ijerph16234860

Trigueros, Aguilar-Parra, López-Liria, & Rocamora. (2019). The Dark Side of the Self-Determination Theory and Its Influence on the Emotional and Cognitive Processes of Students in Physical Education. International Journal of Environmental Research and Public Health, 16(22), 4444. doi:10.3390/ijerph16224444

Trigueros, R., Aguilar-Parra, J. M., Lopez-Liria, R., Cangas, A. J., González, J. J., & Álvarez, J. F. (2020). The role of perception of support in the classroom on the students’ motivation and emotions: The impact on metacognition strategies and academic performance in math and english classes. Frontiers in Psychology, 10, Article 2794. https://doi.org/10.3389/fpsyg.2019.02794

Trigueros, R., Padilla, A., Aguilar-Parra, J. M., Lirola, M. J., García-Luengo, A. V., Rocamora-Pérez, P., & López-Liria, R. (2020). The Influence of Teachers on Motivation and Academic Stress and Their Effect on the Learning Strategies of University Students. International Journal of Environmental Research and Public Health, 17(23), 9089. doi:10.3390/ijerph17239089

Trigueros-Ramos, R., Navarro Gómez, N., Aguilar-Parra, J. M., & León-Estrada, I. (2019). Influence of physical education teacher on confidence, fun, motivation and intention to be physically active in adolescence. Cuadernos de Psicología del Deporte, 19(1), 222-232.

Trouilloud, D., Sarrazin, P., Bressoux, P., & Bois, J. (2006). Relation between teachers' early expectations and students' later perceived competence in physical education classes: Autonomy-supportive climate as a moderator. Journal of educational psychology, 98(1), 75.

Tsai, K. M., Nguyen, H., Weiss, B., Ngo, V. K., & Lau, A. S. (2020). Effects of Family Obligation Values and Autonomy Support on Internalizing Symptoms of Vietnamese-American and European-American Adolescents. Journal of Child and Family Studies, 29(4), 1136-1146.

Tsai, Y. M., Kunter, M., Lüdtke, O., Trautwein, U., & Ryan, R. M. (2008). What makes lessons interesting? The role of situational and individual factors in three school subjects. Journal of Educational Psychology, 100(2), 460.

Tunkkari, M., Aunola, K., Hirvonen, R., Silinskas, G., & Kiuru, N. (2021). The Quality of Maternal Homework Involvement: The Role of Adolescent and Maternal Factors. Merrill-Palmer Quarterly, 67(1), 94-122.

Tunkkari, M., Aunola, K., Hirvonen, R., Silinskas, G., & Kiuru, N. (2021). The interplay between maternal homework involvement, task-avoidance, and achievement among adolescents. Journal of Family Psychology.

Ulstad, S. O., Halvari, H., Sørebø, Ø., & Deci, E. L. (2016). Motivation, Learning Strategies, and Performance in Physical Education at Secondary School. Advances in Physical Education, 06(01), 27-41. doi:10.4236/ape.2016.61010

Valdés-Cuervo, A. A., Grijalva-Quiñonez, C. S., & Parra-Pérez, L. G. (2020). Mothers’ motivational beliefs and children's learning purpose for doing homework: The mediate effects of autonomy support and control. Revista de Psicodidáctica (English ed.), 25(2), 100-108.

Van den Berghe, L., Soenens, B., Vansteenkiste, M., Aelterman, N., Cardon, G., Tallir, I. B., & Haerens, L. (2013). Observed need-supportive and need-thwarting teaching behavior in physical education: Do teachers' motivational orientations matter?. Psychology of Sport and Exercise, 14(5), 650-661.

Van den Berghe, L., Soenens, B., Aelterman, N., Cardon, G., Tallir, I. B., & Haerens, L. (2014). Within-person profiles of teachers' motivation to teach: Associations with need satisfaction at work, need-supportive teaching, and burnout. Psychology of Sport and Exercise, 15(4), 407-417.

van der Kaap‐Deeder, J. (2021). The role of parents’ autonomy support and psychological control in sibling relationship functioning through children’s need‐based experiences. British Journal of Developmental Psychology, 39(4), 653-668.

van der Kaap‐Deeder, J., Vansteenkiste, M., Soenens, B., Verstuyf, J., Boone, L., & Smets, J. (2014). Fostering self‐endorsed motivation to change in patients with an eating disorder: The role of perceived autonomy support and psychological need satisfaction. International Journal of Eating Disorders, 47(6), 585-600.

van der Kaap-Deeder, J., Vansteenkiste, M., Soenens, B., Loeys, T., Mabbe, E., & Gargurevich, R. (2015). Autonomy-supportive parenting and autonomy-supportive sibling interactions: The role of mothers’ and siblings’ psychological need satisfaction. Personality and Social Psychology Bulletin, 41(11), 1590-1604.

van der Kaap-Deeder, J., Vansteenkiste, M., Soenens, B., & Mabbe, E. (2017). Children’s daily well-being: The role of mothers’, teachers’, and siblings’ autonomy support and psychological control. Developmental psychology, 53(2), 237.

Van Der Kaap-Deeder, J., Soenens, B., Mabbe, E., Dieleman, L., Mouratidis, A., Campbell, R., & Vansteenkiste, M. (2019). From daily need experiences to autonomy-supportive and psychologically controlling parenting via psychological availability and stress. Parenting, 19(3), 177-202.

Van der Linden, N., Devos, C., Boudrenghien, G., Frenay, M., Azzi, A., Klein, O., & Galand, B. (2018). Gaining insight into doctoral persistence: Development and validation of Doctorate-related Need Support and Need Satisfaction short scales. Learning and Individual Differences, 65, 100-111.

Van Doren, N., De Cocker, K., De Clerck, T., Vangilbergen, A., Vanderlinde, R., & Haerens, L. (2021). The Relation between Physical Education Teachers’ (De-)Motivating Style, Students’ Motivation, and Students’ Physical Activity: A Multilevel Approach. International Journal of Environmental Research and Public Health, 18(14), 7457. doi:10.3390/ijerph18147457

Van Petegem, S., Brenning, K., Baudat, S., Beyers, W., & Zimmer-Gembeck, M. J. (2018). Intimacy development in late adolescence: Longitudinal associations with perceived parental autonomy support and adolescents' self-worth. Journal of adolescence, 65, 111-122.

Van Petegem, S., Zimmer-Gembeck, M. J., Soenens, B., Vansteenkiste, M., Brenning, K., Mabbe, E., ... & Zimmermann, G. (2017). Does general parenting context modify adolescents' appraisals and coping with a situation of parental regulation? The case of autonomy-supportive parenting. Journal of child and family studies, 26(9), 2623-2639.

Vansteenkiste, M., Zhou, M., Lens, W., & Soenens, B. (2005). Experiences of autonomy and control among Chinese learners: Vitalizing or immobilizing?. Journal of educational psychology, 97(3), 468.

Vansteenkiste, M., Soenens, B., Van Petegem, S., & Duriez, B. (2014). Longitudinal associations between adolescent perceived degree and style of parental prohibition and internalization and defiance. Developmental psychology, 50(1), 229.

Vansteenkiste, M., Sierens, E., Soenens, B., Luyckx, K., & Lens, W. (2009). Motivational profiles from a self-determination perspective: The quality of motivation matters. Journal of educational psychology, 101(3), 671.

Vansteenkiste, M., Sierens, E., Goossens, L., Soenens, B., Dochy, F., Mouratidis, A., ... & Beyers, W. (2012). Identifying configurations of perceived teacher autonomy support and structure: Associations with self-regulated learning, motivation and problem behavior. Learning and instruction, 22(6), 431-439.

Vermote, B., Aelterman, N., Beyers, W., Aper, L., Buysschaert, F., & Vansteenkiste, M. (2020). The role of teachers’ motivation and mindsets in predicting a (de) motivating teaching style in higher education: A circumplex approach. Motivation and Emotion, 44, 270-294.

Vierling, K. K., Standage, M., & Treasure, D. C. (2007). Predicting attitudes and physical activity in an “at-risk” minority youth sample: A test of self-determination theory. Psychology of Sport and Exercise, 8(5), 795-817.

Viksi, A., & Tilga, H. (2022). Perceived Physical Education Teachers’ Controlling Behaviour and Students’ Physical Activity during Leisure Time—The Dark Side of the Trans-Contextual Model of Motivation. Behavioral Sciences, 12(9), 342.

Vlachopoulos, S. P., Katartzi, E. S., Kontou, M. G., Moustaka, F. C., & Goudas, M. (2011). The revised perceived locus of causality in physical education scale: Psychometric evaluation among youth. Psychology of Sport and Exercise, 12(6), 583-592.

Vlachopoulos, S. P., Katartzi, E. S., & Kontou, M. G. (2013). Fitting multidimensional amotivation into the self-determination theory nomological network: Application in school physical education. Measurement in Physical Education and Exercise Science, 17(1), 40-61.

Vrolijk, P., Van Lissa, C. J., Branje, S. J., Meeus, W. H., & Keizer, R. (2020). Longitudinal Linkages Between Father and Mother Autonomy Support and Adolescent Problem Behaviors: Between-Family Differences and Within-Family Effects. Journal of youth and adolescence, 49(11), 2372-2387.

Waaler, R., Halvari, H., Skjesol, K., & Bagøien, T. E. (2013). Autonomy support and intrinsic goal progress expectancy and its links to longitudinal study effort and subjective wellbeing: The differential mediating effect of intrinsic and identified regulations and the moderator effects of effort and intrinsic goals. Scandinavian journal of educational research, 57(3), 325-341.

Wallhead, T. L., Hagger, M., & Smith, D. T. (2010). Sport education and extracurricular sport participation: An examination using the trans-contextual model of motivation. Research quarterly for exercise and sport, 81(4), 442-455.

Wang, Z., & Dong, S. (2019). Autonomy as Core of Creativity and Compliance: Moderated Moderation Model of Maternal Parenting Behaviors. Creativity Research Journal, 31(1), 74-82.

Wang, L. (2017). Using the self-determination theory to understand Chinese adolescent leisure-time physical activity. European journal of sport science, 17(4), 453-461.

Wang, Q., Pomerantz, E. M., & Chen, H. (2007). The role of parents’ control in early adolescents’ psychological functioning: A longitudinal investigation in the United States and China. Child development, 78(5), 1592-1610.

Wang, Q., Chan, H. W., & Lin, L. (2012). Antecedents of Chinese parents’ autonomy support and psychological control: The interplay between parents’ self-development socialization goals and adolescents’ school performance. Journal of youth and adolescence, 41(11), 1442-1454.

Wang, J. C., Morin, A. J., Ryan, R. M., & Liu, W. C. (2016). Students’ motivational profiles in the physical education context. Journal of Sport and Exercise Psychology, 38(6), 612-630.

Wang, J. C. K., Ng, B. L., Liu, W. C., & Ryan, R. M. (2016). Can being autonomy-supportive in teaching improve students’ self-regulation and performance?. In Building autonomous learners (pp. 227-243). Springer, Singapore.

Wang, Y., Qiao, D., & Chui, E. (2018). Student engagement matters: A self-determination perspective on Chinese MSW students’ perceived competence after practice learning. British Journal of Social Work, 48(3), 787-807.

Wang, J., Lai, R., Yang, A., Yang, M., & Guo, Y. (2021). Helicopter parenting and depressive level among non-clinical Chinese college students: A moderated mediation model. Journal of Affective Disorders, 295, 522-529.

Wang, Y., King, R. B., Wang, F., & Leung, S. O. (2021). Need-supportive teaching is positively associated with students' well-being: A cross-cultural study. Learning and Individual Differences, 92, 102051.

Wang, J., Liu, R. D., Ding, Y., Xu, L., Liu, Y., & Zhen, R. (2017). Teacher’s autonomy support and engagement in math: multiple mediating roles of self-efficacy, intrinsic value, and boredom. Frontiers in psychology, 8, 1006.

Wei, D., Zhang, D., He, J., & Bobis, J. (2020). The impact of perceived teachers’ autonomy support on students’ mathematics achievement: evidences based on latent growth curve modelling. European Journal of Psychology of Education, 35(3), 703-725.

Weinstein, N., Chubb, J. A., Haddock, G., & Wilsdon, J. R. (2021). A conducive environment? The role of need support in the higher education workplace and its effect on academics' experiences of research assessment in the UK. Higher Education Quarterly, 75(1), 146-160.

Whipple, N., Bernier, A., & Mageau, G. A. (2011). A dimensional approach to maternal attachment state of mind: Relations to maternal sensitivity and maternal autonomy support. Developmental Psychology, 47(2), 396.

Wijnia, L., Loyens, S. M., Derous, E., & Schmidt, H. G. (2014). Do students’ topic interest and tutors’ instructional style matter in problem-based learning?. Journal of Educational Psychology, 106(4), 919.

Williams, G. C., & Deci, E. L. (1996). Internalization of biopsychosocial values by medical students: a test of self-determination theory. Journal of personality and social psychology, 70(4), 767.

Williams, G. C., Wiener, M. W., Markakis, K. M., Reeve, J., & Deci, E. L. (1994). Medical students’ motivation for internal medicine. Journal of general internal medicine, 9(6), 327-333.

Williams, G. C., Saizow, R., Ross, L., & Deci, E. L. (1997). Motivation underlying career choice for internal medicine and surgery. Social science & medicine, 45(11), 1705-1713.

Won, S., & Shirley, L. Y. (2018). Relations of perceived parental autonomy support and control with adolescents' academic time management and procrastination. Learning and Individual Differences, 61, 205-215.

Wuttke, A. (2020). Political engagement’s non-political roots: examining the role of basic psychological needs in the political domain. Motivation and Emotion, 44(1), 135-150.

Xie, X. L., & Xue, W. X. (2021, June). A Study of Effects of Teaching Styles on Student Engagement: Evidence from E-learning. In 2021 IEEE 4th Advanced Information Management, Communicates, Electronic and Automation Control Conference (IMCEC) (Vol. 4, pp. 1786-1794). IEEE.

Xu, J. (2016). A study of the validity and reliability of the teacher homework involvement scale: a psychometric evaluation. Measurement, 93, 102-107.

Xu, J., Du, J., Cunha, J., & Rosário, P. (2021). Student perceptions of homework quality, autonomy support, effort, and math achievement: Testing models of reciprocal effects. Teaching and Teacher Education, 108, 103508.

Xu, X., Dai, D. Y., Liu, M., & Deng, C. (2019). Parental psychological control and academic functioning in Chinese high school students: A short‐term longitudinal study. British Journal of Developmental Psychology. doi:10.1111/bjdp.12308

Yaban, E. H., & Sayil, M. (2021). The intergenerational similarity of social value orientations in adolescents and emerging adults: Variable-centered and person-centered approaches. Journal of Social and Personal Relationships, 02654075211018271.

Yang, F., & Xu, J. (2019). A psychometric evaluation of teacher homework involvement scale in online learning environments. Current Psychology, 38(6), 1713-1720.

Yarahmadi, Y. (2012). The Explanation and Prediction of the Student's School Performance on the Basis of Explanation of Internal Motivational Factors with Structured Functional Model at Rural in Sanandaj City High Schools. Procedia-Social and Behavioral Sciences, 47, 643-650.

Yew Meng, H., Whipp, P., Dimmock, J., & Jackson, B. (2013). The Effects of Choice on Autonomous Motivation, Perceived Autonomy Support, and Physical Activity Levels in High School Physical Education. Journal of Teaching in Physical Education, 32(2), 131-148.

Yli-Piipari, S., Layne, T., Hinson, J., & Irwin, C. (2018). Motivational pathways to leisure-time physical activity participation in urban physical education: A cluster-randomized trial. Journal of Teaching in Physical Education, 37(2), 123-132.

Yoo, J. (2015). Perceived autonomy support and behavioral engagement in physical education: A conditional process model of positive emotion and autonomous motivation. Perceptual and Motor Skills, 120(3), 731-746. doi:10.2466/06.PMS.120v20x8

Yoon, S., Kim, S., & Kang, M. (2020). Predictive power of grit, professor support for autonomy and learning engagement on perceived achievement within the context of a flipped classroom. Active Learning in Higher Education, 21(3), 233-247.

Young-Jones, A., Levesque, C., Fursa, S., & McCain, J. (2019). Autonomy-supportive language in the syllabus: supporting students from the first day. Teaching in Higher Education, 1-16.

Yu, S., & Levesque-Bristol, C. (2020). A Cross-Classified Path Analysis of the Self-Determination Theory Model on the Situational, Individual and Classroom Levels in College Education. Contemporary Educational Psychology, 101857.

Yu, C., Li, X., & Zhang, W. (2015). Predicting adolescent problematic online game use from teacher autonomy support, basic psychological needs satisfaction, and school engagement: A 2-year longitudinal study. Cyberpsychology, Behavior, and Social Networking, 18(4), 228-233.

Yu, C., Li, X., Wang, S., & Zhang, W. (2016). Teacher autonomy support reduces adolescent anxiety and depression: An 18-month longitudinal study. Journal of adolescence, 49, 115-123.

Zarrinabadi, N., Lou, N. M., & Shirzad, M. (2021). Autonomy support predicts language mindsets: Implications for developing communicative competence and willingness to communicate in EFL classrooms. Learning and Individual Differences, 86, 101981.

Zhang, H., & Whitebread, D. (2019). Identifying characteristics of parental autonomy support and control in parent–child interactions. Early Child Development and Care, 1-14.

Zhang, T., Solmon, M. A., Kosma, M., Carson, R. L., & Gu, X. (2011). Need Support, Need Satisfaction, Intrinsic Motivation, and Physical Activity Participation among Middle School Students. Journal of Teaching in Physical Education, 30(1), 51-68.

Zhang, T., Solmon, M. A., & Gu, X. (2012). The role of teachers’ support in predicting students’ motivation and achievement outcomes in physical education. Journal of Teaching in Physical Education, 31(4), 329-343.

Zhang, D., Jin, B., & Cui, Y. (2021). Do Teacher Autonomy Support and Teacher–Student Relationships Influence Students’ Depression? A 3-Year Longitudinal Study. School Mental Health, 1-15.

Zhao, J., & Qin, Y. (2021). Perceived Teacher Autonomy Support and Students' Deep Learning: The Mediating Role of Self-Efficacy and the Moderating Role of Perceived Peer Support. Frontiers in Psychology, 12, 2177.

Zhou, M., Ma, W. J., & Deci, E. L. (2009). The importance of autonomy for rural Chinese children's motivation for learning. Learning and individual differences, 19(4), 492-498.

Zhou, L. H., Ntoumanis, N., & Thøgersen-Ntoumani, C. (2019). Effects of perceived autonomy support from social agents on motivation and engagement of Chinese primary school students: Psychological need satisfaction as mediator. Contemporary Educational Psychology, 58, 323-330.

Zimmer-Gembeck, M. J., Webb, H. J., Thomas, R., & Klag, S. (2015). A new measure of toddler parenting practices and associations with attachment and mothers' sensitivity, competence, and enjoyment of parenting. Early Child Development and Care, 185(9), 1422-1436.

Zimmer-Gembeck, M. J., Joyce, J., Kerin, J., Webb, H., Morrissey, S., & McKay, A. (2019). Self-determination theory and food-related parenting: The Parent Socioemotional Context of Feeding Questionnaire. Journal of Family Psychology, 33(4), 476.

Zimmermann, J., Tilga, H., Bachner, J., & Demetriou, Y. (2021). The Effect of Teacher Autonomy Support on Leisure-Time Physical Activity via Cognitive Appraisals and Achievement Emotions: A Mediation Analysis Based on the Control-Value Theory. International journal of environmental research and public health, 18(8), 3987.

Zong, X., Zhang, L., & Yao, M. (2018). Parental involvement and Chinese elementary students’ achievement goals: the moderating role of parenting style. Educational Studies, 44(3), 341-356.
